# Tracking the Elusive Function of *Bacillus subtilis* Hfq

**DOI:** 10.1371/journal.pone.0124977

**Published:** 2015-04-27

**Authors:** Tatiana Rochat, Olivier Delumeau, Nara Figueroa-Bossi, Philippe Noirot, Lionello Bossi, Etienne Dervyn, Philippe Bouloc

**Affiliations:** 1 Institute for Integrative Biology of the Cell (I2BC), CEA, CNRS, Université Paris-Sud, F-91405, Orsay, France; 2 INRA, UR892, Virologie et Immunologie Moléculaires, F-78352, Jouy-en-Josas, France; 3 INRA, UMR1319 Micalis, F-78350, Jouy-en-Josas, France; 4 AgroParisTech, UMR Micalis, F-78350, Jouy-en-Josas, France; 5 Institute for Integrative Biology of the Cell (I2BC), CEA, CNRS, Université Paris-Sud, F-91190, Gif-sur-Yvette, France; Max-Planck-Institute for Terrestrial Microbiology, GERMANY

## Abstract

RNA-binding protein Hfq is a key component of the adaptive responses of many proteobacterial species including *Escherichia coli*, *Salmonella enterica* and *Vibrio cholera*. In these organisms, the importance of Hfq largely stems from its participation to regulatory mechanisms involving small non-coding RNAs. In contrast, the function of Hfq in Gram-positive bacteria has remained elusive and somewhat controversial. In the present study, we have further addressed this point by comparing growth phenotypes and transcription profiles between wild-type and an *hfq* deletion mutant of the model Gram-positive bacterium, *Bacillus subtilis*. The absence of Hfq had no significant consequences on growth rates under nearly two thousand metabolic conditions and chemical treatments. The only phenotypic difference was a survival defect of *B*. *subtilis hfq* mutant in rich medium in stationary phase. Transcriptomic analysis correlated this phenotype with a change in the levels of nearly one hundred transcripts. Albeit a significant fraction of these RNAs (36%) encoded sporulation-related functions, analyses in a strain unable to sporulate ruled out sporulation *per se* as the basis of the *hfq* mutant’s stationary phase fitness defect. When expressed in *Salmonella*, *B*. *subtilis hfq* complemented the sharp loss of viability of a *degP hfq* double mutant, attenuating the chronic σ^E^-activated phenotype of this strain. However, *B*. *subtilis hfq* did not complement other regulatory deficiencies resulting from loss of Hfq-dependent small RNA activity in *Salmonella* indicating a limited functional overlap between *Salmonella* and *B*. *subtilis* Hfqs. Overall, this study confirmed that, despite structural similarities with other Hfq proteins, *B*. *subtilis* Hfq does not play a central role in post-transcriptional regulation but might have a more specialized function connected with stationary phase physiology. This would account for the high degree of conservation of Hfq proteins in all 17 *B*. *subtilis* strains whose genomes have been sequenced.

## Introduction

Hfq is a RNA-binding protein that plays a crucial role in post-transcriptional regulation in many bacteria (reviewed in refs [1–3]). *Escherichia coli* Hfq (Hfq_Ec_) was first shown to be required for RNA phage Qß replication [4], but its function in uninfected host cells remained unknown for a long time. In 1994, the discovery of phenotypes associated with *hfq*_*Ec*_ insertion mutants revealed that Hfq_Ec_ was important for bacterial physiology [5], but the origin of these phenotypes was only progressively uncovered through the discovery of Hfq-dependent regulatory RNAs.

Base-pairing association of regulatory RNAs with RNA-partner molecules is a conserved mechanism to regulate gene expression. In bacteria, regulatory RNAs, usually small and noncoding, affect mRNA translation and stability to modulate numerous processes, including plasmid replication, envelope homeostasis, iron homeostasis, virulence and central metabolism (reviewed in ref. [6]). Small RNAs (sRNAs) that are expressed from genetic regions unlinked to their targets are referred to as *trans*-encoded RNAs; in these instances, base-pairing is often imperfect with a limited nucleotide complementarity.

In many bacteria including *E*. *coli*, *Salmonella* and *Vibrio*, Hfq is required for the activity of most, if not all, *trans*-encoded RNAs. Hfq protects sRNAs against degradation by ribonucleases and is thought to stimulate pairing with their targets [7]. As a result, Hfq alterations in these organisms typically produce highly pleiotropic effects. While most of these affects can be ascribed to the loss of sRNA-mediated regulation, some evidence suggests that Hfq can also affect gene expression directly, via sRNA-independent pathways [8].

Orthologs of the *hfq* gene are found in about half of the bacterial genomes [9]; however, their involvement in RNA regulatory mechanisms is sometimes unclear, in particular within the *Bacilli* class (*i*.*e*., *Staphylococcus aureus*, *Listeria monocytogenes* and *Bacillus subtilis*). While the Staphylococcal Hfq (Hfq_Sa_) structure has been known for more than ten years [10], there is little information about its activity. In several pathogenic isolates, Hfq_Sa_ is either poorly or not expressed [11–13] and consistently, deletion of its gene does not impact the physiology of these isolates [11]. On the other hand, in strains where Hfq_Sa_ has been detected, deletion of *hfq*_*Sa*_ reportedly affected strain toxicity and virulence [13]. Although Hfq_Sa_ was shown to associate with RNAs *in vitro*, no discernible effect on sRNAs mediated translational repression has been demonstrated *in vivo* [12,14–16], therefore questioning the role of Hfq_Sa_ in this type of regulation. In addition, Hfq_Sa_ cannot complement for the absence of its homolog in *Salmonella*, Hfq_STM_ [17], indicating that the two proteins are not functionally equivalent.

Deletion of the *L*. *monocytogenes hfq* gene (*hfq*_*Lm*_) did not affect growth, except upon salt, ethanol or Triton X-100 exposure [18]. Three RNAs binding Hfq_Lm_ were identified [19]. One of them, LhrA, down-regulates expression of three genes and its stability is affected in an Hfq-dependent manner [20,21]. However, Hfq_Lm_-dependent sRNA stability does not seem to be a general feature, since the abundance of other twelve sRNAs was not affected by Hfq_Lm_ [22], and a comparative transcriptome analysis of the *hfq*_*Lm*_ mutant with its isogenic parental strain also indicated that none of the identified sRNAs were affected by the absence of Hfq [23]. Recent structural studies indicate that Hfq_Lm_-RNA interactions differ from those established by the Hfq proteins of other Gram-positive bacteria [24,25].

Several recent studies point to a minor role of Hfq_Bs_ in *B*. *subtilis* physiology. The growth rates of *hfq*_*Bs*_ mutants in glucose-supplemented minimal media are identical to those of the corresponding wild-type strains [26,27]. Hfq_Bs_ associates *in vivo* with sRNAs and the 5’ leader regions of some mRNAs and its absence affect the abundance of few sRNAs [28]. In at least one case, Hfq_Bs_ was implicated in the translation of an mRNA (*ahrC*) [29,30]. However, the regulatory activities of several sRNAs were found to be Hfq_Bs_-independent [26,29–32]. Thus, despite the extensive knowledge on the structure of the Hfq protein from *B*. *subtilis* and other Gram-positive bacteria, the physiological role(s) of these proteins remain(s) poorly understood.

In this study, we have carried out a systematic and comprehensive analysis of the physiology of an Hfq_Bs_ deletion mutant as compared to the wild type strain. We discovered that the main consequence of the *hfq*_*Bs*_ deletion is a decreased fitness in stationary phase. This defect correlated with a change in the levels of approximately 100 transcripts including transcripts related to sporulation and to Type-I toxin-antitoxin (TA) systems. However, we found the fitness defect to be independent of sporulation process and of the presence of *txpA*-RatA and *bsrE*-as-BsrE Type-I toxin-antitoxin (TA) systems. While this work was under way, an article describing a similar analysis of a *B*. *subtilis hfq* mutant appeared in the press [27]. The results of latter study concord with ours in some aspects (stationary phase fitness phenotype) but differ in others (transcriptional profiling). We discuss possible sources of these discrepancies. Overall, however, the results from the two studies point to the conclusion that *hfq*_*Bs*_ is not a major player of sRNA-mediated regulation but its integrity is essential to ensure the bacterial survival under starvation conditions.

## Results

### Patterns of *hfq*_*Bs*_ expression

**Fig 1:**
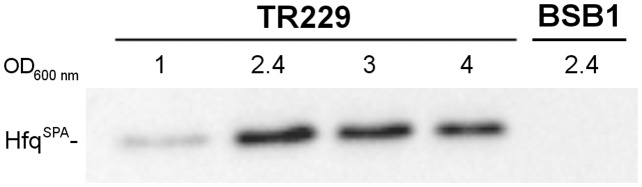
Expression of Hfq_Bs_ during growth. Cultures of strain carrying *hfq*_*Bs*_ *-spa* translational fusion (TR229) or wild-type strain (BSB1) were performed in LB medium at 37°C under vigorous agitation. Harvested cells from exponential, early and late transition and stationary phase cultures (OD_600nm_ 1, 2.4, 3 and 4, respectively) were lysed by sonication and 2.75 μg of total protein extracts of each sample were separated by electrophoresis in 12.5% SDS-PAGE. Hfq_Bs_^SPA^ was detected by immunoblotting using anti-FLAG M2 antibodies (Sigma).

In the course of a large scale transcriptome study, we observed that *hfq*_*Bs*_ is transcribed under all conditions tested, with initiation occurring at three distinct promoters: *i*) a σ^A^-dependent promoter located upstream *miaA*, *ii*) a σ^H^-dependent promoter upstream *hfq*_*Bs*_ active in stationary phase (see also [33]) and *iii*) an early stage sporulation promoter (σ^EF^) leading to *ymaF-miaA-hfq*_*Bs*_ transcript (S1 File and ref. [34]). Two recent studies used translational gene fusions to the chromosomal *hfq*_*Bs*_ locus to measure Hfq_Bs_ expression levels as a function of the growth phase in synthetic media. While the first study concluded that Hfq levels increase in cells in stationary phase [28], the second study reported no significant difference between all growth phases [27]. We also independently constructed a strain with the *hfq*_*Bs*_ gene terminally fused to the peptide affinity (SPA) tag (containing three FLAG epitope repeats). The resulting strain, BSB1 *hfq*_*Bs*_::SPA (TR229), expresses the Hfq_Bs_::SPA protein under the control of the *hfq*_*Bs*_ native promoter as a unique source of Hfq. When TR229 is grown in rich medium (LB), Hfq_*Bs*_::SPA significantly accumulates during the transition from exponential to stationary phase as revealed by Western blotting (Fig 1).

### High throughput phenotypic analysis of the Δ*hfq*_*Bs*_ mutant

In many bacteria, defective phenotypes resulting from Hfq inactivation can be revealed by exposing cells to stress conditions (*e*.*g*., oxidative stress, iron starvation, high temperature). Therefore, we sought to identify possible phenotypic alterations by comparing the growth of wild-type *B*. *subtilis* (BSB1, ref. [34]) and its isogenic Δ*hfq* derivative (TR223, *cf*. Material and Methods) under numerous conditions. Nearly two thousand conditions were tested by performing a phenotype microarray analysis (*cf*. Material and Methods) [35]. No growth differences were observed between the two strains when tested for the utilization of various carbon, nitrogen, phosphorus and sulfur sources, or when cells were subjected to nutrient upshifts or conditions affecting osmolarity or pH (S2 File). Beside a resistance to amphenicol of Δ*hfq*_*Bs*_ strain associated with presence of a chloramphenicol cassette in the mutant strain, very few effects related to chemicals were detected. The phenotype microarray analysis suggested that the Δ*hfq* strain could be more resistant to compound 48/80 and more sensitive to the 2,4-Diamino-6,7-diisopropylpteridine and cetylpyridinium as compared to the wild-type strain. However, further tests showed these observations to be artifacts of the microarray analysis since no growth differences exist between the wild-type strain and its Δ*hfq* derivative grown in the presence of these three compounds (S2 File). We concluded that Hfq_Bs_ does not affect the growth rate or growth yield of *B*. *subtilis* under any of the tested conditions. Thus, Hfq_Bs_ does not appear to have the same impact on adaptation responses as its homologs in enteric bacteria.

### Hfq_Bs_ improves survival in stationary phase

**Fig 2:**
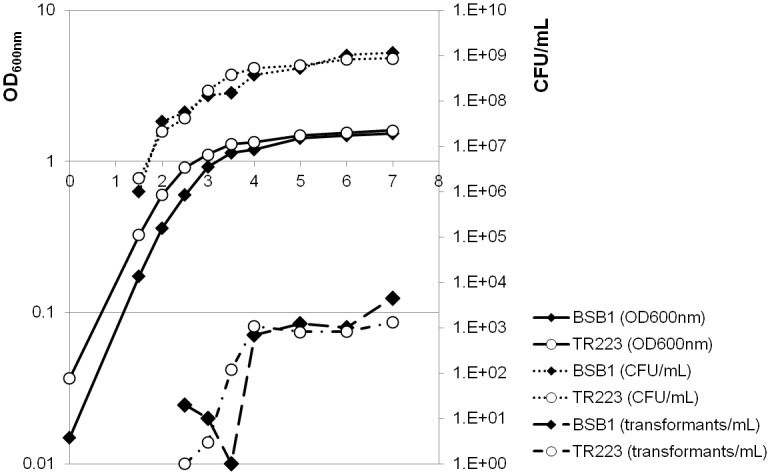
Competence efficiency. The apparition and the proportion of competent cells in cultures of wild-type (BSB1) and Δ*hfq* mutant (TR223) strains were monitoring by calculating the number of cells able to integrate an antibiotic resistance gene in their chromosomal DNA during the competence process development.

One of the three promoters transcribing the *hfq*_*Bs*_ gene is under the control of σ^H^ (S1 File, ref. [33]). This alternative sigma factor directs the transcription of genes required for cellular adaptation during transition from exponential to stationary phase, including induction of sporulation, genetic competence or biofilm development [36,37]. The observed increase in the amount of Hfq_Bs_ in transition and stationary phases (Fig 1 and ref [28]) likely reflects activation of *hfq*_*Bs*_ transcription from the σ^H^ promoter. We wondered whether Hfq_Bs_ activity could be linked to σ^H^-dependent phenotypes. The ability of wild-type and Δ*hfq*_*Bs*_ strains to form biofilms or to uptake exogenous DNA was monitored, but no significant difference was observed (S3 File and Fig 2).

**Fig 3:**
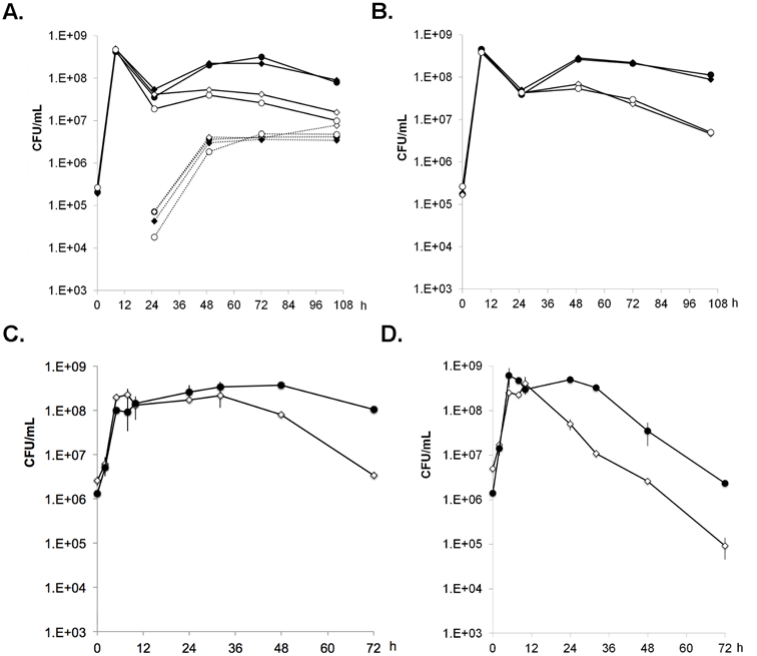
Survival of *B*. *subtilis* Δ*hfq*_*Bs*_ in competition with *hfq*-expressing strain. A) Co-cultures were performed with *hfq*_*Bs*_-expressing strain and Δ*hfq*_*Bs*_ mutant in LB medium and incubated at 37°C during 5 days. An antibiotic resistance gene (*cat* or *spc*) was inserted in the intergenic region *hfq-ymzE* (control, black) or in replacement of the *hfq*_*Bs*_ coding sequence (mutant, white). Each population was numbered on LB plates supplemented with spectinomycin or chloramphenicol. To number spores, samples were incubated 15 min at 80°C before plating (dot lines). Two combinations of co-cultures were performed using two sets of isogenic strains (●) TR247 (*hfq*_*Bs*_^+^ *igr*::*cat*) and (◊) TR232 (Δ*hfq*_*Bs*_::*spc igr*+) or strains (○) TR223 (Δ*hfq*_*Bs*_::*cat igr*+) and (♦) TR241 (*hfq*_*Bs*_^+^ *igr*::*spc*). B) The same experiments were performed with sporulation-deficient derivative strains (*sigE*::*erm*). The two co-cultures were performed using strains (●) TR243 and (◊) TR237 or strains (○) TR235 and (♦) TR245. C) Competition experiments were performed with TR259 and TR255 strains which are deleted of the *bsrE*-asBsrE type 1 TA and express (●) or not (◊) Hfq_*Bs*_. D) Competition experiments were performed with the TF8A (*txpA*-RatA deleted) derivative strains using Δ*hfq*_*Bs*_ TR248 (◊) and control TR252 (●) strains.

We also considered that Hfq_Bs_ could contribute to *B*. *subtilis* survival in stationary phase. To assess this possibility, we compared the viability of *hfq*_*Bs*_^+^ and Δ*hfq*_*Bs*_ strains in co-cultures in rich medium. Each strain had a specific antibiotic resistance gene—either *cat* (chloramphenicol) or *spc* (spectinomycin)—inserted either in the *hfq*_*Bs*_ gene (alleles Δ*hfq*_*Bs*_::*cat* and Δ*hfq*_*Bs*_::*spc*) or in the intergenic region (*igr*) between *hfq*_*Bs*_ and *ymzE* (alleles *igr*::*cat* and *igr*::*spc*). The permutation of resistance markers allowed correcting for possible loss of fitness arising from the antibiotic resistance gene. Samples from the co-cultures were plated on selective media and survival was assayed counting the corresponding colony forming units (CFU). This analysis revealed that the loss of *hfq*_*Bs*_ results in a decreased survival in aging cultures (Fig 3A). This is in agreement with data recently reported and shows that *hfq*_*Bs*_ positively affects the survival of bacterial cells independently of the growth medium (*i*.*e*. minimal CS-glucose [27] *vs* LB [this study]).

### Transcriptome analysis of the *hfq*_*Bs*_ mutant: RNA changes in stationary phase

In *Enterobacteria*, loss of Hfq results in a reduced stability of many sRNAs and the concomitant deregulation of the corresponding mRNA targets under unperturbed conditions (reviewed in refs. [1–3]). Differences in RNA patterns between the wild-type and Δ*hfq* strains of *B*. *subtilis* could reveal potential Hfq_Bs_-targets and possibly provide insight into the molecular basis for the observed stationary phase phenotype. Therefore, the transcriptomes of these two strains were analyzed by comparing tiling array hybridization profiles of RNAs extracted from cells cultured in rich medium (LB) at two different growth stage: *i*) exponential phase (OD_600nm_ = 0.5) and *ii*) 5 hours after the onset of stationary phase. Transcripts were positioned on our *B*. *subtilis* 168 structural annotation map [34] and changes were investigated by differential expression analysis (*cf*. Material and Methods).

Somewhat surprisingly, the RNA profiles from exponentially growing cells were identical in the wild-type and Δ*hfq*_*Bs*_ strains, except for the obvious absence of the *hfq*_*Bs*_ RNA in the Δ*hfq*_*Bs*_ mutant (S4 and S5 Files). Many regulatory sRNAs were detected, including FsrA [31] (S512), RatA [26] (S976), RoxS (Ncr22 or S415) [32,38,39] (alias RsaE in *S*. *aureus* [15,40]), as-*bsrE* (Ncr1019, S718) as well as 16 other sRNAs of unknown function. None of these sRNAs, nor their known mRNA targets were affected by the absence of Hfq_Bs_ (S5 File and S1 Table). Thus, Hfq_Bs_ does not appear to influence *B*. *subtilis* RNA patterns during the exponential phase to any significant extent, at least in cells grown in rich medium. The situation might be different in minimal CS-glucose-grown cells where 68 mRNAs and one sRNA (ncr1670; S357) were reported to be affected by Hfq_Bs_ inactivation [27].

Unlike the results from the exponential samples, 97 transcription units (representing 134 genes) were found significantly different between the wild-type and the Δ*hfq*_*Bs*_ strains in the stationary cultures (S6 File). Fold-change values and functional information are available in S1 Table. Many of the affected RNAs are from genes under the control of stationary phase-specific transcriptional regulators suggesting that Spo0A was activated in Δ*hfq*_*Bs*_ strain as exemplified by the deregulation of 18 genes of its regulon [41]. Overall, 48 out of the 134 genes are linked to sporulation. Portions of the AbrB, SigH and SigD regulons also show their expression changed in the absence of Hfq. Some genes are related to respiration and anaerobiosis: the *rex*-*ndh* operon appears upregulated in the Δ*hfq*_*Bs*_ strain whereas the *ctaDEF* operon together with the *cccA* and *qoxB* loci are down-regulated. The envelope stress response controlled by SigM is also slightly activated in the absence of Hfq (S1 Table). Besides the modifications observed for protein-coding genes, the absence of Hfq affected the amount of *i*) two sRNAs: S1022, a SigD-induced RNA [42], and S1495 (unknown function); *ii*) four riboswitches (upstream of *thiC*, *trpS*, *ileS* and *thrZ*); and *iii*) two type-I toxin-antitoxin systems (namely the antisense RNAs as-*bsrE* and *ratA* as well as the toxin *txpA*). We considered that the changes in RNA profiles might arise from the upregulation of Hfq_Bs_ in stationary phase (Fig 1) and might help explaining the mechanism responsible for Hfq_Bs_ contribution to survival under these conditions (Fig 3A).

### The survival advantage conferred by Hfq_Bs_ is independent of sporulation

Sporulation is an energy and time consuming process that is used by *B*. *subtilis* as a last resource for survival when other adaptation programs like exogenous DNA-uptake or cannibalism have failed [43]. Based on the transcriptomic profiles, we hypothesized that under nutrient deprivation, cells lacking *hfq* may enter sporulation earlier than wild-type cells, thus losing the chance to use the residual nutrients available. To test if the survival defect observed in the *hfq* mutant was dependent of sporulation, the competition experiment (see above) was repeated in cells carrying the combinations of *hfq* and *igr* alleles in the background of a sporulation-deficient (Δ*sigE*::*erm*) mutant. Results showed the Δ*hfq*_*Bs*_ deletion to still confer a survival defect in this background (Fig 3B) suggesting that the defect is maintained in vegetative cells and thus unlikely to relate to sporulation.

### The survival advantage conferred by Hfq is independent of BsrE or TxpA type 1 Toxin-Antitoxin systems

Among the RNAs affected by the *hfq*_*Bs*_ deletion in stationary phase are antitoxin *as-bsrE* and RatA antisense RNAs (S1 Table). A third antitoxin RNA, as-*bsrG* (ncr1932), was found in reduced amounts in a separate study [28]. Altogether these findings raised the possibility that alteration in the regulation of toxin/antitoxin systems might be responsible for the decreased fitness of the *hfq*_*Bs*_ mutant. However, further analyses ruled out this possibility as well. Deleting the entire *bsrE*/as-BsrE type-1 TA system did not relieve the survival defect of the *hfq*_*Bs*_ mutant (Fig 3C). Furthermore, the survival defect associated with the Δ*hfq*_*Bs*_ deletion was still observed in a *B*. *subtilis* strain cured for the SKIN and SP-β, prophages, which, combined, contribute four different TA systems, namely TxpA and BsrH (SKIN) and BsrG and YonT (SP-β) (Fig 3D).

### Complementation tests in *Salmonella*

**Fig 4:**
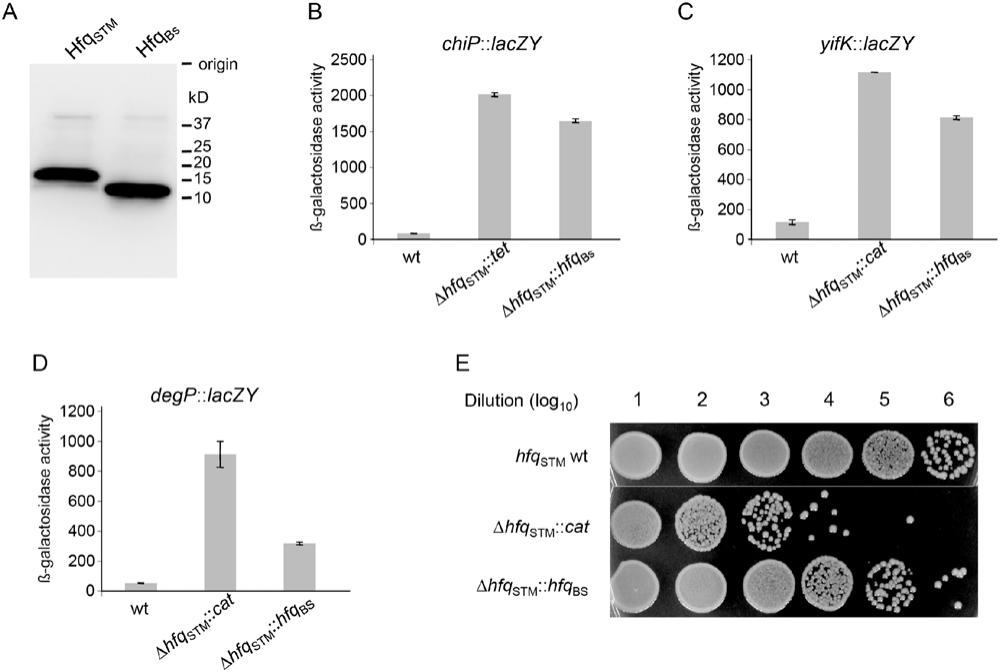
Expression of translational *lacZ* fusions to chromosomal genes sensitive to Hfq function in *Salmonella enterica* and growth phenotype of *degP*::*lacZ* strains carrying different *hfq* alleles. (A). *S*. *enterica* strains carrying *hfq*_*ST*_ *-flag* (MA11054) or *hfq*_*Bs*_ *-flag* (MA12275) translational fusion were grown in LB medium at 37°C under vigorous agitation. Harvested cells were lysed and crude extracts were used for western blot analysis using anti-FLAG M2 antibodies. ß-galactosidase activity was measured in exponentially growing LB cultures (OD_600_ ≈ 0.3) (B and C) or in early stationary phase cultures (OD_600_ ≈1.5) (D). Strains used were: B. MA9132, MA10744 and MA11214; C. MA8020, MA8021, and MA11215; D. MA9591, MA9603, and MA11216 (see Table 1 for full genotypes). (E) Cultures from strains in D were incubated 24 hours in stationary phase, serially diluted, and spotted on LB agar. *hfq*_*STM*_ (top row); Δ*hfq*_*STM*_ (middle row); Δ*hfq*_*STM*_:: *hfq*_*Bs*_ (bottom row).

In *Salmonella* and *E*. *coli*, Hfq-regulated genes provide simple, yet sensitive assays for monitoring Hfq function *in vivo*. To take advantage of this system, we replaced the coding portion of the *Salmonella hfq* gene (*hfq*_*STM*_) with the corresponding segment of the *B*. *subtilis* (*hfq*_*Bs*_). Using a strategy previously reported [17], we demonstrated that *hfq*_Bs_ and *hfq*_STM_ were both expressed in a similar amount (Fig 4A). We then tested the effects of the gene exchange on the regulation of *chiP* and *yifK* genes, regulated by Hfq-dependent ChiX and GcvB sRNAs, respectively [44,45]. Deleting the *hfq*_*STM*_ gene in *Salmonella* (by replacement with an antibiotic resistance cassette) leads to a marked derepression of *lacZ* gene fusions to the two genes. Replacing the endogenous *hfq*_*STM*_ gene with *B*. *subtilis hfq*_*Bs*_ does not correct the regulatory defect of *chiP* and *yifK* fusions to a significant extent (Fig 4B and 4C), indicating that Hfq_Bs_ cannot substitute for the endogenous protein in ChiX- and GcvB-mediated regulation.

In enteric bacteria, the σ^E^–driven envelope stress response is activated in *hfq* mutants due to the over-accumulation of several outer membrane proteins (OMP) [46–48]. The σ^E^-controlled, Hfq-dependent MicA and RybB sRNAs are thought to be mainly responsible and ensure a negative feedback control [17,47,49]. We also tested the effects of the *hfq*_*STM*_ / *hfq*_*Bs*_ gene exchange on the regulation of *degP*, a member of the σ^E^ regulon that is chronically upregulated in an *hfq* defective background. The σ^E^ alteration is significantly alleviated in cells expressing Hfq_Bs_ (activation ratio dropping from more than 20-fold to less than 8-fold; Fig 4D). In the course of these experiments, it became apparent that the Δ*hfq* Δ*degP* double mutant suffers a dramatic loss of viability in stationary phase, presumably reflecting the lack of DegP-mediated processing of the toxic products that are responsible for σ^E^ activation. We thus tested whether the strain with the replaced *hfq* showed a similar survival defect. Expression of *hfq*_*Bs*_ suppresses most of the stationary phase lethality, resulting in a 100-fold increase of viability in the double mutant (Fig 4E). Thus, it appears that Hfq_Bs_ can, at least partially, perform the function(s) that avoid(s) the gratuitous induction of the σ^E^-dependent envelope stress response (see Discussion).

## Discussion

The absence of *hfq*_*Bs*_ i) did not affect the strain growth rate and yield in nearly two thousand tested conditions and, ii) had no effect on the transcriptome of *B*. *subtilis* growing exponentially in a rich medium despite the fact that numerous small non-coding RNAs were expressed during the conditions of these experiments. Similar studies performed in close species led to the same results, namely no growth defect for *S*. *aureus* in more than a thousand tested conditions [11] and no transcriptome variations for *L*. *monocytogenes* [23]. Nevertheless, Hfq is required in sustaining *B*. *subtilis* optimal survival upon starvation. To try dissecting this phenotype at the molecular level, we initially focused on the 171 RNA fragments reported to co-immunoprecipitate (CoIP) with Hfq [28]. Surprisingly, only seven mRNAs and two antisense RNAs associated with Hfq were found affected by the *hfq* deletion in our study. Additional transcriptomic data from a *B*. *subtilis hfq* deleted strain became available while our study was under way [27]. In this case only five mRNAs found altered in the mutant [27] were in common with the RNAs identified by CoIP [28]. Note that only two of these RNAs (*ctaD* and *yebD*) correspond to those identified in our study (S1 Table). While the latter discrepancy could be ascribed to differences in the growth conditions, the lack of correlation between the Hfq_Bs_ CoIP and the two independent Hfq_Bs_ transcriptomic data suggests that Hfq_Bs_ associates with RNA molecules but has little effect on their stability. Hfq may have only a limited number of specific partners or may influence regulatory RNA network by translational regulation.

The comparison between the transcriptomic data from cells grown either in rich medium (this study) or minimal CS-glucose medium [27] shows extensive differences. On the one hand, no sporulation-linked RNAs were affected by the Δ*hfq* deletion in minimal medium. On the other hand, ResD/Rex (adaptation of anaerobiosis), GerE (germination) and ComA (competence) regulons, reportedly activated in the *hfq* mutant in minimal medium [27], were unaffected in our study. Only seven protein-coding genes were found in common but all of them, except one being affected in the opposite ways. Despite these discrepancies, both studies showed a decreased fitness of the *hfq* mutant in stationary phase regardless the medium used. We tested by competition experiments the possible involvement of *yebD* or *ctaD* (as their corresponding mRNAs were also found associated with Hfq [28]) and concluded these two genes are not associated with the *hfq* survival phenotype (S7 File). To date, the molecular mechanism responsible for this phenotype remains unidentified.

Hammerle *et al* proposed that the *hfq*-dependent phenotype was related to competence or sporulation [27]. However, we showed that the *hfq* deletion did not affect competence efficiency and that the survival advantage conferred by Hfq remained in a sporulation deficient strain (Figs 2 and 3). As several Type-I TA systems had been reported to have their expression level modified in the absence of Hfq or were coIP with Hfq (this study and refs. [27,28]), one can speculate the existence of a functional link that could explain the survival advantage phenotype. We showed here that the fitness conferred by Hfq was independent of the presence of several Type-I TA systems (Fig 3). Altogether, these data suggest that as observed for Hfq_Lm_ and Hfq_Sa_, Hfq_Bs_ has a minor or no role on RNA stability in almost all conditions and are likely required only for starvation adaptation of vegetative cells.

*hfq*-ortholog genes are found in most sequenced proteobacteria and their role is well established in some of them. The Firmicutes division includes the Bacilli and Clostridia classes and within the last class, *Clostridium difficile* has an Hfq protein recently shown to be involved in sRNA mediated-regulations and sporulation gene expression [50,51]. In contrast, within the Bacilli, members of the Bacillales class (*e*.*g*., *B*. *subtilis*, *L*. *monocytogenes*, *S*. *aureus*) have a conserved *hfq* gene with an unclear function, while the Lactobacillales do not contain an *hfq* homolog. The selective pressure for *hfq* maintenance within the Bacillales is not strict, possibly because Hfq had a limited number of targets. In agreement with this proposal, the sRNA-dependent regulations reported within the Bacilli class are usually Hfq-independent, with the noticeable exception of LhrA in *L*. *monocytogenes* [19].

The only evidence for a molecular phenotype associated with Hfq_Bs_ is its requirement for the mRNA *ahrC* translation [29,30]. Despite that the mRNA *ahrC* is the target of SR1 sRNA, the Hfq effect is SR1 independent. Hfq_Bs_ acts as a specific post-transcriptional regulator, possibly by refolding the mRNA to promote its translation. As AhrC controls arginine metabolism, activating the catabolism pathway when arginine is available, we hypothesized that a lack of translation could explain the survival defect of the *hfq* mutant upon starvation. A translational fusion *ahrC*::*spa* was constructed at the locus in wild-type and Δ*hfq* strains and the quantity of AhrC was measured by Western-blot in cells grown in LB rich medium. No difference of AhrC protein level was observed in exponential phase and we failed to detect the protein in stationary phase (S8 File). A strong accumulation of SR1, which represses *ahrC* expression, has been reported in stationary phase [30] and it probably explains the observed strong repression of *ahrC*. These data do not support a major role of AhrC in the survival defect observed in the absence of Hfq upon starvation.

As an alternative way for probing the biological activity of *B*. *subtilis* Hfq, we examined whether, and to what extent, it corrected some of the phenotypes resulting from Hfq inactivation in *Salmonella enterica*. We found Hfq_Bs_ not to complement the regulatory defect of *chiPQ* and *yifK* loci to a relevant extent, suggesting that *B*. *subtilis* Hfq cannot interact with, or support the activity of, neither ChiX nor GcvB sRNAs. A third *hfq* mutant phenotype tested was the chronic activation of the *Salmonella* σ^E^ response. Interestingly, the expression of *hfq*_*Bs*_ significantly reduced the levels of *Salmonella* σ^E^ induction. At the same time, Hfq_Bs_ suppressed the acute loss of viability suffered by a *degP* mutant lacking endogenous Hfq in stationary phase. Hfq_Bs_ may affect Hfq-dependent sRNAs responsible for a negative feedback regulation, or additional uncharacterized components involved in the regulation [8]. Alternatively Hfq could down-regulate OMP production directly through a sRNA-independent mechanism. The latter possibility might be relevant in the framework of the present study, as it would account for the observation that *B*. *subtilis* Hfq lacks the capacity of mediating sRNA activity, yet partially complements the σ^E^-related phenotype of a Salmonella *hfq* mutant.

The *hfq*_*Bs*_ gene is found in the 49 sequenced genomes of the Bacillus genus (S9 File) indicating a strong requirement for its maintenance. Hfq_Bs_ contribution to stationary phase survival gives a rational for its conservation within the specie. Interestingly, Hfq_Bs_ accumulates in stationary phase and the only *hfq*_*Bs*_-dependent previously reported phenotype was also observed in stationary phase [29]. Hfq_Bs_ clearly does not play the wide role attributed to its enteric bacteria counterparts. However, adaptation of *B*. *subtilis* to stationary phase represents a crucial process for this soil bacterium which encounters important and repeated variations in nutrient availability altering feast and starvation. In a competitive natural environment, Hfq_Bs_ might play an essential role in promoting survival, possibly via stationary phase-dependent translational regulation.

## Material and Methods

### Strain constructions

Strains and primers used in this study are listed in Table 1 and S2 Table, respectively.

**Table 1:** *S*trains used in this work.

*Strain* ^a^	*Genotype*	*Source or reference*
BSB1	prototroph	[34]
TR223	Δ*hfq*_*Bs*_::*cat*	This work
TR229	pMUTIN-*hfq*_*Bs*_::*SPA*, *Em* ^*r*^	This work
TR232	Δ*hfq*_*Bs*_::*spc*	This work
TR234	Δ*sigE*::*erm*	This work
TR235	Δ*sigE*::*erm* Δ*hfq*_*Bs*_::*cat*	This work
TR237	Δ*sigE*::*erm* Δ*hfq*_*Bs*_::*spc*	This work
TR241	*igr*::*spc*	This work
TR247	*igr*::*cat*	This work
TR243	Δ*sigE*::*erm* igr::*cat*	This work
TR245	Δ*sigE*::*erm* igr::*spc*	This work
Bas028	P_wt_ *ahrC-SPA*::*erm*	This work
Bas101	Δ*hfq*_*Bs*_::*cat*, P_wt_ *ahrC-SPA*::*erm*	This work
Bas103	Δ*hfq*_*Bs*_::*spc*, P_wt_ *ahrC-SPA*::*erm*	This work
TF8A	*trpC2*; ΔSPβ; *skin*; ΔPBSX	[55]
TR248	*trpC2*; ΔSPβ; *skin*; ΔPBSX; Δ*hfq*_*Bs*_::*spc*	This work
TR252	*trpC2*; ΔSPβ; *skin*; ΔPBSX; *igr*::*cat*	This work
JJS-Din048	*trpC2*; ΔSPβ; *skin*; ΔPBSX; *pycA-ctaG*::*phleo*	[56]
EDJ1125	*trpC2*; ΔSPβ; *skin*; ΔPBSX; *pycA-ctaG*::*phleo;* Δ*hfq*_*Bs*_::*spc*	This work
EDJ1129	*trpC2*; ΔSPβ; *skin*; ΔPBSX; *pycA-ctaG*::*phleo; igr*::*cat*	This work
TR259	*igr*::*cat;* Δ*bsrE*::*erm*	This work
TR255	Δ*hfq*_*Bs*_::*spc;* Δ*bsrE*::*erm*	This work
TR257	*igr*::*cat;* Δ*yebD*::*erm*	This work
TR253	Δ*hfq*_*Bs*_::*spc;* Δ*yebD*::*erm*	This work
MA8020	*yifK87*::MudK	[47]
MA8021	*yifK87*::MudK Δ*hfq67*::*cat*	[47]
MA9132	*chiP91*::pCE40(*lacZY*)	[44]
MA9591	*degP1*::pCE40 (l*acZY*)	[8]
MA9603	*degP1*::pCE40 (*lacZY*) Δ*hfq67*::*cat*	[8]
MA10740	Δ*hfq116*::*tetAR*/pKD46	[17]
MA10744	*chiP91*::pCE40(*lacZY*) Δ*hfq116*::*tetAR*	
MA11054	*Hfq*_*STM*_ *-*3xFLAG*-aph* (KnR)	[17]
A11214	*yifK87*::MudK Δ*hfq*_*STM*_::*hfq*_*Bs*_	This work
MA11215	*chiP91*::pCE40(*lacZY*) Δ*hfq*_*STM*_::*hfq*_*Bs*_	This work
MA11216	*degP1*::pCE40 (l*acZY*) Δ*hfq*_*STM*_::*hfq*_*Bs*_	This work
MA12275	*hfq*_*Bs*_ *-*3xFLAG*-aph* (KnR)	This work

^a^ All *B*. *subtilis* strains (name starting by BSB and TR) are derived from BSB1 which is a tryptophan-prototrophic (trp+) of *B*. *subtilis* 168 [34] excepted TF8A derivative strains. All *Salmonella* strains (name starting with MA) are derived from *Salmonella enterica* serovar Typhimurium strain MA3409 which is a derivative of strain LT2 cured for the Gifsy-1 prophage [60].

The *B*. *subtilis* strains BSB1 Δ*hfq*_*Bs*_::*cat* (TR223) and Δ*hfq*_*B*s_::*spc* (TR232) were constructed by homologous replacement of the Hfq_Bs_ coding sequence with a chloramphenicol (*cat*) or spectinomycin (*spc*) resistance gene, respectively, using a joining PCR technique [52]. The *cat* and *spc* genes were amplified using 1151/1152 primers with pHV1610IR(-) and pIC156 as templates, respectively [53,54]. DNA fragments corresponding to the upstream and downstream *hfq* sequences were PCR amplified using 1147/1148 and 1149/1150 primers, respectively, with BSB1 chromosomal DNA as a template. DNA fragments were purified using NucleoSpin Gel clean-up (Macherey Nagel) and then joined by a second PCR using 1147 and 1150 primers. The *hfq* deletion mutant was obtained by transformation of the BSB1 strain with the joining PCR fragment and selection for chloramphenicol (5 μg/ml) or spectinomycin (100 μg/ml) resistance. The same procedure was used to construct a control strain carrying the *cat* or *spc* gene inserted into the intergenic region between *hfq* and *ymzE* genes. Briefly, DNA fragments upstream and downstream the insertion position were amplified using 1186/1187 and 1188/1154 primers, respectively, and then were joined with the *cat* or *spc* genes by a second PCR using 1186/1154 primers. Transformation of the BSB1 strain resulted in TR247 (Cm^r^) and TR241 (Spc^r^) strains.

The BSB1 sporulation-deficient strain was obtained by replacement of the sig*E* coding sequence by an erythromycin (*erm*) resistance gene amplified using 1151/1152 primers with pMUTIN as a template [52]. Upstream and downstream DNA fragments were amplified using 1182/1183 and 1184/1185 primers, respectively. The joining PCR fragment was amplified using 1182/1185 primers and transformed into BSB1 strain resulting in BSB1 Δ*sigE*::*erm* (TR234). TR234 strain was transformed with chromosomal extracts of Δ*hfq*_*Bs*_::*cat*, Δ*hfq*_*Bs*_::*spc*, *igr*::*spc* or *igr*::*cat* resulting in the corresponding sporulation-deficient derivative strains TR235, TR237, TR243 and TR245, respectively. The Δ*hfq*_*Bs*_::*spc* or *igr*::*cat* were also introduced by transformation with chromosomal extracts in four other genetic backgrounds: i) the TF8A strain (deleted of skin, PBSX and SP-β prophages, [55], resulting in TR248 and TR252 strains respectively; ii) BSB1-derivative strain deleted of the *bsrE*-asBsrE type 1 TA system, resulting in TR255 and TR259 strains; iii) the JJS-Din048 strain, a TF8A-derivative strain deleted of the genomic region from *pycA* to *ctaG* genes [56] resulting in EDJ1125 and EDJ1129 strains, and iv) a BSB1-derivative strain deleted of *yebD* gene, resulting in TR253 and TR257 strains (see Table 1). The *yebD* and *bsrE*-asBsrE mutants were obtained by gene replacement with *erm* gene amplified using TRO84/TRO85 primers. Upstream and downstream DNA fragments were amplified using TRO80/TRO81 and TRO82/TRO83 primers, respectively for *yebD* deletion, or using TRO86/TRO87 and TRO88/TRO89 primers, respectively for *bsrE* deletion. The joining PCR fragments were amplified using TRO80/TRO83 or TRO86/TRO89 primers for *yebD*::*erm* and *bsrE*::*erm* fragments respectively, and transformed into BSB1 strain.

A BSB1 strain expressing a C-terminal SPA-tagged Hfq_Bs_ protein was constructed as previously described [57] by chromosomal integration of a translational fusion between the *hfq* coding sequence and the sequential peptide affinity (SPA) tag sequence [58] resulting in the TR229 strain expressing Hfq_Bs_^SPA^ under the control of its native promoter. The pMUTIN-SPA plasmid was used as in ref. [59]. A PCR amplification encompassing almost the entire *hfq* gene—except the ATG start codon—was obtained using the ODfw/ODrev primers. After digestion by Acc65I and NcoI the DNA fragment was ligated to the pMUTIN-SPA that was linearized by the same restriction enzymes. After establishment in *E*.*coli*, the plasmid was recovered by using a Plasmid purification kit (Qiagen) and used to transform competent *B*. *subtilis* BSB1 cells. Integration of the plasmid in the chromosome at the *hfq* locus was selected by plating on LB supplemented with 30 μg erythromycin and 0.5 mM IPTG. Correct insertion was checked by sequencing. The same procedure was used to construct *ahrC*::*spa* translational fusion at the locus resulting in strain Bas028 (Table 1). Bas028 was then transformed by genomic DNA from TRO223 (Δ*hfq*::*cat*) and from TRO232 (Δ*hfq*::*spc*) to produce strains Bas101 and Bas102 respectively.

*Salmonella enterica* serovar Typhimurium strains used in this study are listed on Table 1. They are all derived from MA3409, a derivative of strain LT2 cured for the Gifsy-1 prophage [60]. Salmonella strains carrying the structural portion of the *hfq* gene from *B*. *subtilis* were constructed with a two-step recombineering procedure as described [61]. Strain MA10740, carrying a *tetAR* module inserted in the *hfq* gene of the *Salmonella* chromosome was used with a DNA fragment amplified from chromosomal DNA of *B*. *subtilis* BSB1 with primer pairs 1174/1175, the entire *hfq*::*tetAR* was crossed out selecting for the loss of tetracycline resistance.

Introduction of the 3xFLAG epitope at the 3’ ends of the coding sequence of *hfq*_*Bs*_ was carried out using DNA fragments amplified from plasmid pSUB11, as described [62].

Generalized transduction was carried out using the high frequency transducing mutant of phage P22, HT 105/1 *int-201* [63]. “λ Red”-mediated chromosomal recombineering was carried out as described [64]. Constructs were verified by PCR and DNA sequence.

### Bacterial growth conditions

Bacteria were cultured at 37°C under vigorous agitation in liquid media or in media solidified by the addition of 1.5% (w/v) Difco agar. LB broth [1% bacto tryptone (w/v), 0.5% Difco yeast extract (w/v), 0.5% NaCl (w/v)] was used as complex medium. When needed, LB medium was supplemented with 0.2% (w/v) arabinose and 0.5 μg/ml IPTG. When needed, Antibiotics were included at the following final concentrations: chloramphenicol, 10 μg/ml for *S*. *Typhimurium* and 5 μg/ml for *B*. *subtilis*; kanamycin monosulphate, 50 μg/ml; sodium ampicillin 100 μg/ml; tetracycline hydrochloride, 25 μg/ml; erythromycin, 0.6 μg/ml; spectinomycin, and 100 μg/ml.

Competition experiments were performed as follow: overnight cultures of *hfq*_*Bs*_^+^ and Δ*hfq*_*Bs*_ strains were diluted in same flask containing 50 mL of medium without antibiotic with pairwise assembled as follows: TR241 (igr::*spc*) with TR223 (Δ*hfq*_*Bs*_::*cat*), TR247 (igr::*cat*) with TR232 (Δ*hfq*_*Bs*_::*spc*), TR245 (Δ*sigE*::*erm* igr::*spc*) with TR235 (Δ*sigE*::*erm* Δ*hfq*_*Bs*_::*cat*), TR243 (Δ*sigE*::*erm igr*::*cat*) with TR237 (Δ*sigE*::*erm* Δ*hfq*_*Bs*_::*spc*), TR252 with TR248, EDJ1125 with EDJ1129, TR259 with TR255, and TR257 with TR253. For each co-culture, viable cells of the two populations were numbered by plating serial dilutions on plates supplemented with either chloramphenicol or spectinomycin. For spore numbering, samples were heated at 80°C during 15 min before plating.

Biofilm formation was analyzed as previously described [65]. Cells were grown overnight in LB medium and biofilm formation was tested in MSgg medium (5 mM potassium phosphate [pH 7], 100 mM morpholinepropane sulfonic acid [pH 7], 2 mM MgCl_2_, 700 μM CaCl_2_, 50 μM MnCl_2_, 50 μM FeCl_3_, 1 μM ZnCl_2_, 2 μM thiamine, 0.5% glycerol, 0.5% glutamate, 50 μg of tryptophan/ml, 50 μg of phenylalanine/ml). For that purpose, each overnight culture was diluted 200 times to inoculate either 1 ml of MSgg within a well of a 48-wells microtiter plate or 20 ml of MSgg in 100ml glass bottles. Cultures were incubated without shaking at 30°C, and their pellicles were analyzed by visual inspection during 72 h.

Competence development time course was analyzed as follows: overnight cultures in LB medium supplemented with 50 μg of tryptophan/ml and 3 mM MgSO_4_ were diluted in the same medium and grown at 37°C under vigorous agitation until O.D. 600nm around 1. These cultures were diluted in 10 ml of MD medium (10.7 g/l K_2_HPO_4_, 6 g/l KH_2_PO_4_, 1g/l trisodium citrate, 2% glucose, 0.1% tryptophan, 11 ug/ml ferric ammonium citrate, 3 mM MgSO_4_, 0.25 mg/ml potassium aspartate) to begin the time course competence assay. At different time, 1 ug of chromosomal DNA *amyE*::*KnR* (around 2 10^8^ molecules) were added to 0.2 ml of cells, incubated 20 minutes at 37°C and then spread for transformants selection on LB plates supplemented by kanamycine 6 ug/ml.

### Western-Blot analysis

Hfq_Bs_-3xFlag and Hfq_STM_-3xFlag were detected in *Salmonella enterica* strains as previously described [17]. Hfq^SPA^ and AhrC^SPA^ were detected as following: Cells grown in LB were taken at the end of the exponential phase and in the early stationary phase of growth for AhrC^SPA^, and in exponential, early and late transition and stationary phase (OD_600nm_ 1, 2.4, 3 and 4, respectively) for Hfq^SPA^. After disruption by sonication in 500 μL of 10 mM Tris-Cl pH 7.5, 150 mM NaCl and 1 mM EDTA, samples were centrifuged at 18000g for 30 min at 4°C. The protein concentrations of the samples were measured by the Bradford method and used to load equal quantities of sample proteins on a SDS-PAGE. Proteins were then transferred to a Amersham Hybond-P membrane. Hfq^SPA^ and AhrC^SPA^ were detected by western blot using anti-FLAG M2 antibodies as primary antibody and an anti-mouse IgG-peroxidase antibody (SIGMA A2304) as the second antibody. ECL kit was used to enable the immunodetection and the signal was recorded by using Chemidoc from BioRad.

### RNA preparation and transcriptome analysis

Total RNA was isolated as previously described [34] except for cells lysis. Briefly, overnight cultures were diluted 2000 times in preheated LB medium and incubated at 37°C under shaking agitation (180 rpm) until OD_600nm_ reached 0.5 (exponential growth phase transcriptome) or 5 hours after the onset of stationary phase (stationary phase transcriptome). Cultures were centrifuged; the pellets were frozen in liquid nitrogen and stored at -80°C until RNA extraction. Cells pellets were resuspended into 400 μL of Lysis buffer (4 M guanidine thiocyanate, 25 mM sodium acetate pH 5.2, 5 g/L N-laurylsarcosinate), transferred into FastPrep tubes containing 0.6 g of glass beads (G4649, Sigma) and 400 μL of acid phenol:chloroform:IAA (25:24:1). Bacteria were mechanically lysed by using the Fastprep apparatus (MP Biomedicals) with 3 cycles of 45 s at speed 6.0 separated by incubation on ice during 5 min. After lysis, tubes were centrifuged 15 min at 17,900 g at 4°C. The aqueous phase was acid phenol extracted and isopropanol precipitated as previously described [34]. 40 μg RNA were treated using the Turbo DNase I (Ambion) and purified using the RNA Clean-Up and Concentration Micro Kit (Norgen). The quality of the RNA preparations was assessed using RNA Nano Chip with an Agilent 2100 Bioanalyzer (Agilent Technologies). Synthesis of Cy3-labeled DNA from the RNA samples with random priming, one-color hybridization on tiling arrays and signal acquisition were carried out as previously described [38]. An aggregated expression value was computed for all transcripts according to our recently published *B*. *subtilis* 168 structural annotation which contains 1583 defined transcribed regions in addition to the 4292 previously annotated coding sequences [34]. Expression values were quantile-normalized between experiments and a differential analysis was performed using the Limma R package [66]. P-values were corrected for multiple testing using the Bonferroni-Holm method. Genes with adjusted p-value <0.05 were considered differentially expressed in the Δ*hfq* mutant relative to wild-type.

The data discussed in this publication have been deposited in NCBI's Gene Expression Omnibus [67] and are accessible through GEO Series accession number GSE66893 (http://www.ncbi.nlm.nih.gov/geo/query/acc.cgi?acc=GSE66893).

### Phenotype microarray analysis

A full array phenotype microarray analysis was performed by the Biolog Company (Hayward, CA, USA) according to their standard procedure. The principle is to compare isogenic pairs of strains (here BSB1 and TR223) for their growth on wells of microtiter plates (panels), each well containing a different growth medium. This high-throughput technology allows the testing of a large number of phenotypes (eight metabolic-array panels and twelve sensitivity-array panels). The experiment was run in duplicate (strains/conditions) and pairwise comparison was created to analyze the results. All putative phenotypes were independently verified using disk diffusion assays as follows: exponentially growing cultures of wild-type and Δ*hfq*_*Bs*_ strains were diluted 50 times in 10 ml of LB and used to inoculate three LB plates for each strain. The diameters of growth inhibition around disks containing vibriostatic agent 0.129 (Biorad #53872 0.5 mg), 5 μl of cetylpyridinium chloride 10% (Acros organics) or 5 μl of 48/80 compound (5 mg/ml, Sigma) were measured after 24 h of incubation at 37°C.

### β-galactosidase assays

Activity of ß-galactosidase was measured in toluene-permeabilized cells as described [68] and is expressed in Miller units. Reported values were the average of at least two independent determinations, each involving duplicate or triplicate samples.

## Supporting Information

S1 File *hfq*_Bs_ expression. (PDF)

S2 File BSB1 and BSB1 Δ*hfq*_*Bs*_ phenotype MicroArrays analysis. (PDF)

S3 File Biofilm formation of BSB1 and BSB1 Δ*hfq*_*Bs*_. (PDF)

S4 File BSB1 and BSB1 Δ*hfq*_*Bs*_ transcriptome in exponential growth phase: Scatter plot and selected windows (*hfq*_*Bs*_ mRNA and sRNAs). (PDF)

S5 File BSB1 and BSB1 Δ*hfq*_*Bs*_ transcriptome in exponential growth phase. Genome-wide representation of transcriptome profiles of wild-type and Δ*hfq* strains in exponential phase of growth (Figure legend in S4 File).
(PDF)

S6 File BSB1 and BSB1 Δ*hfq*_*Bs*_ transcriptome in stationary phase. Genome-wide representation of transcriptome profiles of wild-type and Δ*hfq* strains in stationary phase of growth (Figure legend in S4 File).
(PDF)

S7 File BSB1 Δ*hfq*_*Bs*_ survival in competition with *hfq*-expressing cells in the absence of *yebD* or *ctaD*. (PDF)

S8 File *ahrC* expression in *hfq*_*Bs*_-expressing strain and Δ*hfq*_*Bs*_ mutant. (PDF)

S9 File *In silico* analysis of the *hfq*_*Bs*_ gene conservation and synteny among the *Bacillus* genus. (PDF)

S1 Table Transcriptome quantile-normalized expression values of BSB1 and BSB1 Δ*hfq*_*Bs*_ and list of genes differentially expressed in stationary phase. (XLSX)

S2 Table DNA oligonucleotides for PCRs. (PDF)
