# Supplementary material for: Tracking the Elusive Function of Bacillus subtilis Hfq
Source: PLoS One. 2015 Apr 27;10(4):e0124977. doi: 10.1371/journal.pone.0124977 (PMC4410918; doi:10.1371/journal.pone.0124977)
Supplement: S1 File — (PDF) [file pone.0124977.s001.pdf]

*hfq*Bs expression

The expression of *hfq*Bs gene (formerly *ymaH*) in various biological conditions can be visualized using the BaSysBio Transcriptome Browser at http://genome.jouy.inra.fr/cgi-bin/seb/viewdetail.py?id=ymaH_1867373_1867594_1.[1]

Three promoters (U1382, U1384, U1385) were previously identified upstream of *hfq*Bs. The corresponding sequences (boxed text) were extracted from Supplementary Table S4 of reference 1 (http://genome.jouy.inra.fr/basysbio/bsubtranscriptome/).

ymaF-*miaA*-*ymaH*

```
        -35   SigEF (U1382)         -10
taactcgttcatgttttctttcctcctgtaataaagtgtagatgatgcagtgtattcaccctgcccggatttgat
tgggcgaatcgaggaaaaggaggatgtgaacacattgggcctgtatcagtctgactggtcaaaagcaccgccccacgc
acatgcttatcatgtcaaaacaacgagagaacagggccattatcatctcatcgaagggtttactcagccggcaaacgg
atcaaataccgatcagcatacacattactatacagggatcacttcatttgaaaacggccattttcatcggtattacgg
aatctcaggaccggcgattcccttagcagatggcacacattatcatgaaatcgaagaaacgacgtatctggcctataa
cgagccgattgagatccagtacggagggttgtgtacgatcccggagatgacagaagaaaaacgcatcgtcataccct
gaaaggaaagacaagggaaattgtcggcaatgagccgctcggctggtagaaggatgttta
                              SigA (U1384)             -10
ccgatgcaaaaaaagggcaaaatggataggtggttgtccatgttgaatgctataatgggggagatttataaaag
agagtgatacatattgaataatacgaagcagcccgttgtcattttagtcggaccgacggcagtggggaaaaccaattt
aagtattcagctagccaaatccttaaacgcggaaattatcagcggagattcgatgcagatttataaaggatggatat
tggaacagctaaaattaccgaacaggagatggagggagtgccccatcatctgattgacattttagatccccaagactc
tttctctactgccgattatcaaagcttagtaagaaataaaatcagcgagattgcaaatagaggaaagcttccgatgat
tgtcggcggtacagggctttatatacaatctgtgctttacgattatacatttacggaagaggcaaatgatcccgtgtt
tcgagagagcatgcaaatggctgctgagcgggaaggcgctgactttcttcatgccaaacttgctgcagcagatcccga
ggcagcagctgcgattcatccgaataatacaagaagagtcattcgcgcactggaaattttacatacgtccggaaaaac
gatgtcccagcatttgaaggaacaaaaacgagaacttctgtacaatgcagtgttaattggcctgacaatggatagaga
cacgctttacgaaagaattaatcagcgggtcgatttgatgatgcagtcaggccttcttccggaagtgaaacgcttata
cgacaagaacgtgagagactgtcaatcaatacaggcgataggctataaagagctgtatgcatattttgacggttttgt
gacactttccgatgctgtcgaacagctaaagcagaactcgaggcggtatgcgaaacgccagctgacgtggtttcgcaa
caaaatgcaggtcacatggttcgatatgacaccgcctgttga
                                         -35     SigH (U1385)      -10
tatggagctgaaaaaaaaggaaattttcacacatatagcaggaaaactcgaactttaatcgaaactgtatgatat
```

agagaatcaaggaggacgaaacatgaaaccgattaatattcaggatcagtttttgaatcaaatccggaaagaaaatac
gtatgtcactgtttttttgctgaacggctttcagttgcggggccaggtgaaaggctttgataactttaccgtattgtt
ggaatcggaaggtaagcagcagcttatatataaacatgcgatctcaacgtttgcgccgcaaaaaaacgtccagcttga
actcgaatagatcaaaaaatgccatgtcaagacatgaggaaaggctgtcgggggttcccggcggcc

Reference

1. Nicolas P, Mader U, Dervyn E, Rochat T, Leduc A, et al. (2012) Condition-dependent transcriptome reveals high-level regulatory architecture in *Bacillus subtilis*. Science 335: 1103-1106.
