# Supplementary material for: Tracking the Elusive Function of Bacillus subtilis Hfq
Source: PLoS One. 2015 Apr 27;10(4):e0124977. doi: 10.1371/journal.pone.0124977 (PMC4410918; doi:10.1371/journal.pone.0124977)
Supplement: S2 File — (PDF) [file pone.0124977.s002.pdf]

BSB1 and BSB1 Δ*hfq*$_{Bs}$ phenotype MicroArrays analysis

A Phenotype MicroArray^{TM} (PM) analysis was performed in duplicate with the wild-type (BSB1) and the Δ*hfq*$_{Bs}$ mutant (TR223) at 37°C. All plates passed reproducibility analysis (see below the consensus plate from duplicates).

Consensus profiles plate (TR223 in green *versus* BSB1 in red)

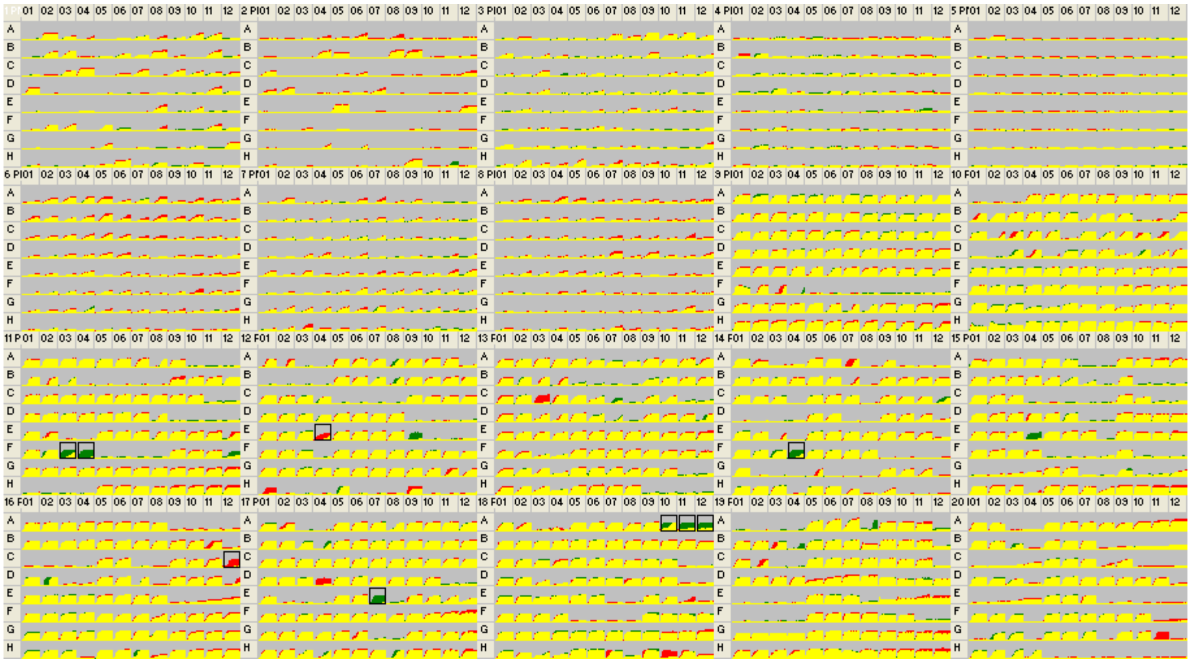

Pairwise comparisons were created. Positive differences may be gained phenotypes or resistance of the Δ*hfq* mutant relative to the wild-type strain. Negative differences may be lost phenotypes or sensitivity of the Δ*hfq* mutant strain relative to the wild-type strain. Detailed PM maps (PM1 to 20) used in this study are available at the Biolog website (http://www.biolog.com/pdf/pm_lit/PM1-PM10.pdf and http://www.biolog.com/pdf/pm_lit/PM11-PM20.pdf).

1. Carbon utilization (PM1,2): No differences
2. Nitrogen utilization (PM3,6,7,8): No differences
3. Phosphorus and Sulfur (PM4A): No differences
4. Nutrient Stimulation (PM5): No differences
5. Osmolarity and pH (PM9, 10): No differences

6. Chemical sensitivity (PM11-20): a relative resistance was observed to compound 48/80 (PM17, E7), and amphenicols (PM18C, A10-A11-A12; PM11C, F03-F04, PM14A, F04) and a relative sensitivity was seen to 2,4-Diamino-6,7-diisopropylpteridine (O.129 vibriostatic agent, PM12, E4), and cetylpyridinium chloride (PM16, C12).

Chemical sensitivity phenotypes were tested independently by diffusion disk assays (see Material and Methods). Differences observed for the three chemical produces by the high throughput Biolog assay were no confirmed (see table below).

	BSB1	TR223
2,4-Diamino-6,7-diisopropylpteridine	14 mm	13 mm
Cetylpyridinium chloride	10 mm	10 mm
48/80 compound	11 mm	12 mm
