# Supplementary material for: Tracking the Elusive Function of Bacillus subtilis Hfq
Source: PLoS One. 2015 Apr 27;10(4):e0124977. doi: 10.1371/journal.pone.0124977 (PMC4410918; doi:10.1371/journal.pone.0124977)

BSB1 and BSB1 Δ*hfq*$_{Bs}$ biofilm formation

Biofilms formations were followed during time in two conditions small or large diameter either on plastic or glass supports. By visual inspection, no difference could be observed for the biofilm formation in both cases. After 72 hours, biofilms showed the same shape and the same color indicating a similar biofilm aging for both strains. The microtiter plates were then slowly tilted and in both cases the biofilm could stay organized indicating similar biofilm strength and attachment to plastic wells. The *hfq* deletion did not significantly affect biofilm formation or its organization.

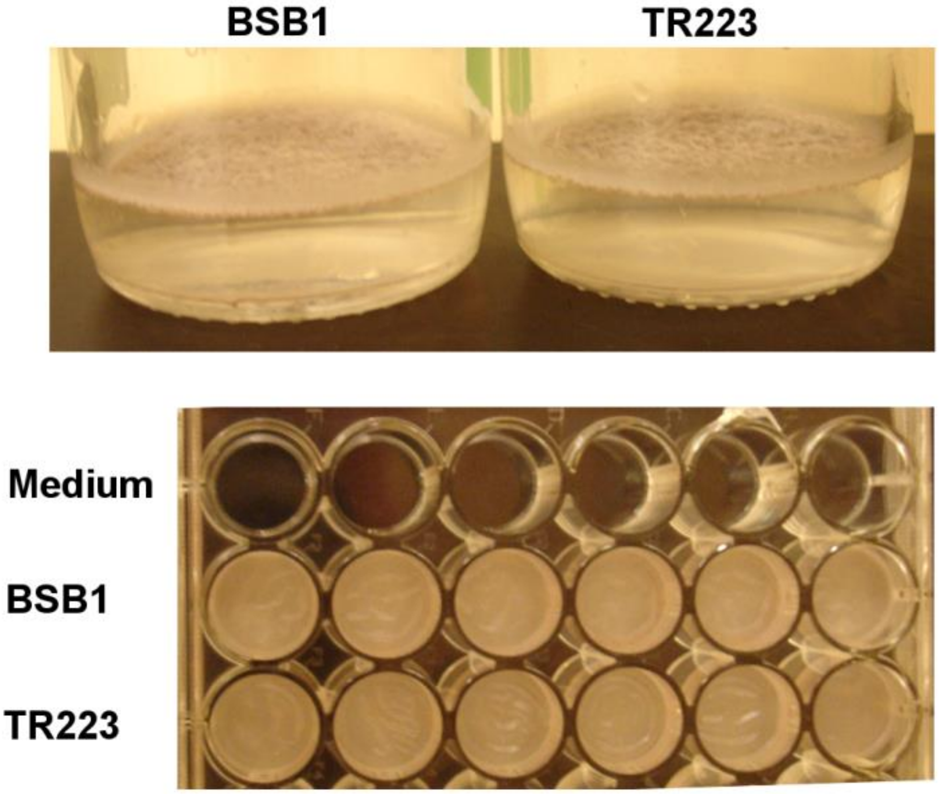

Supplement: S3 File — (PDF) [file pone.0124977.s003.pdf]
