# Supplementary material for: Tracking the Elusive Function of Bacillus subtilis Hfq
Source: PLoS One. 2015 Apr 27;10(4):e0124977. doi: 10.1371/journal.pone.0124977 (PMC4410918; doi:10.1371/journal.pone.0124977)
Supplement: S4 File — (PDF) [file pone.0124977.s004.pdf]

BSB1 and BSB1 Δ*hfq*$_{Bs}$ transcriptome in exponential growth phase:

Scatter plot and selected windows (*hfq*$_{Bs}$ mRNA and sRNAs)

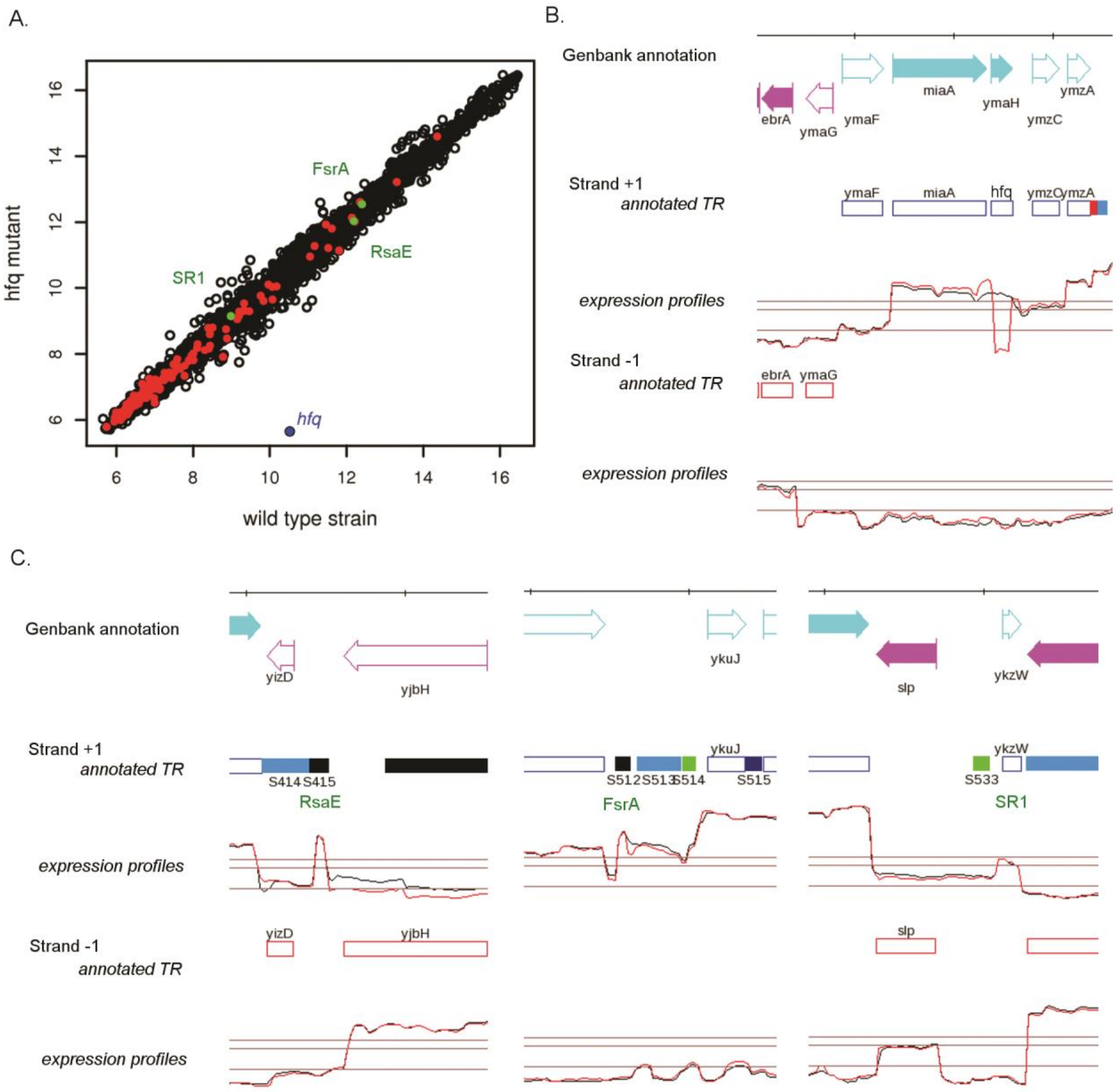

A. Log2-scale scatter plots of transcriptional intensities of each annotated transcribed region (TR) in *Δhfq* cells (*y*-axis) compared to those of wild-type cells (*x*-axis). The annotated TR consist of all genes reported in Genbank AL009126.3 and transcription segments (named S1 to

S1583) defined previously (ref. 1). Red dots correspond to 138 TRs previously classified as 'Indep' (independent segments transcribed from their own promoter), a category which includes known regulatory RNAs as SR1, FsrA (S512) or RsaE (S415), marked as green dots. B-C. Each of the 4 panels represents a chromosome region with annotations of CDSs and TRs and expression profiles on forward strand (+1) and backward strand (-1) for the wild-type strain (black line, BSB1) or the Δ*hfq* mutant (*red line, TR223).* Cut-off levels are marked with horizontal lines indicating 1-, 5- and 10-fold above background level. Panel B shows expression profiles for hfq_{Bs} *gene; Panel C shows chromosomal regions encoding for known regulatory RNAs* (RsaE, FsrA and SR1). The dataset for the complete genome is available in Supporting information S5.
