# Supplementary figures and images for: Tracking the Elusive Function of Bacillus subtilis Hfq
Source: PLoS One. 2015 Apr 27;10(4):e0124977. doi: 10.1371/journal.pone.0124977 (PMC4410918; doi:10.1371/journal.pone.0124977)

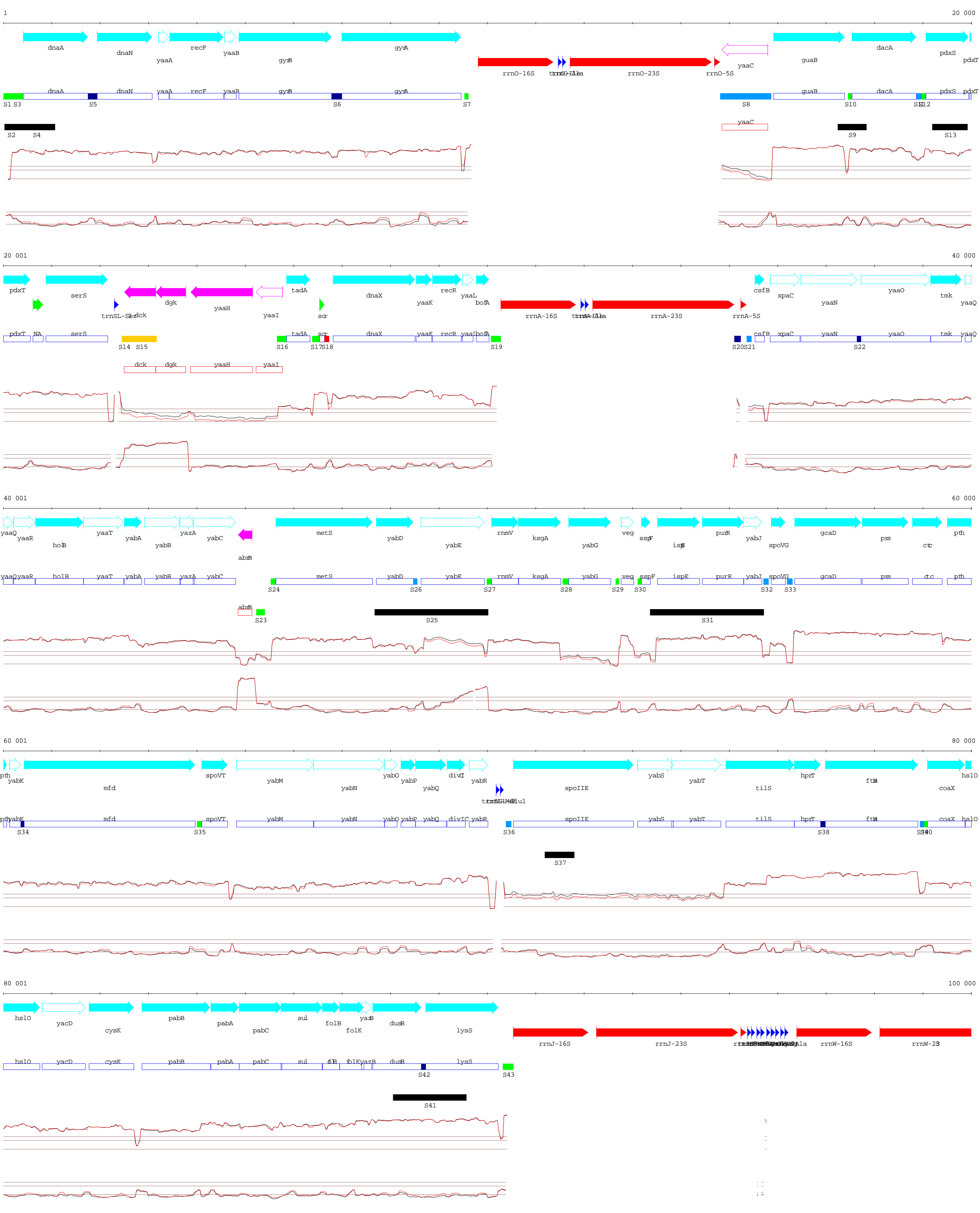

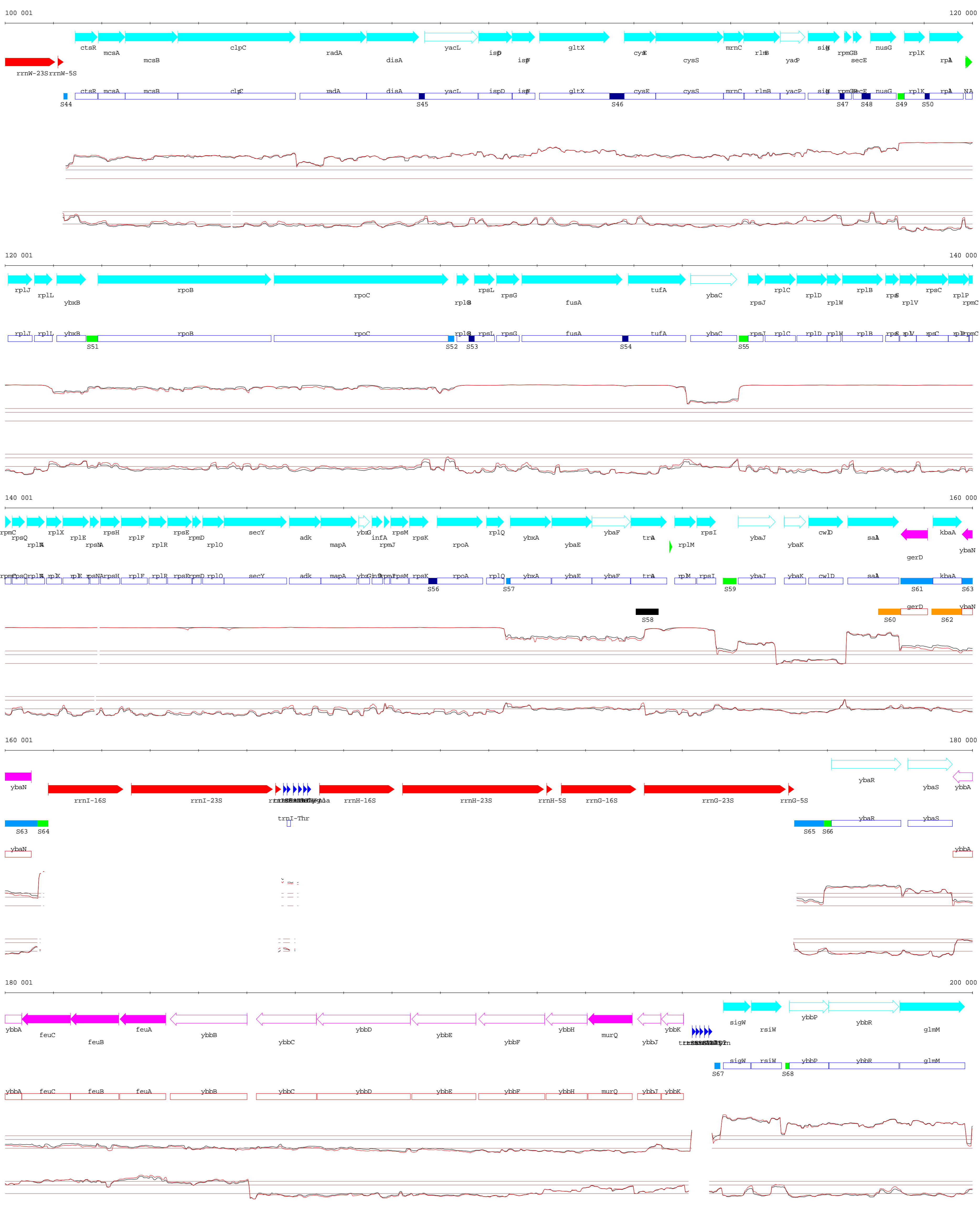

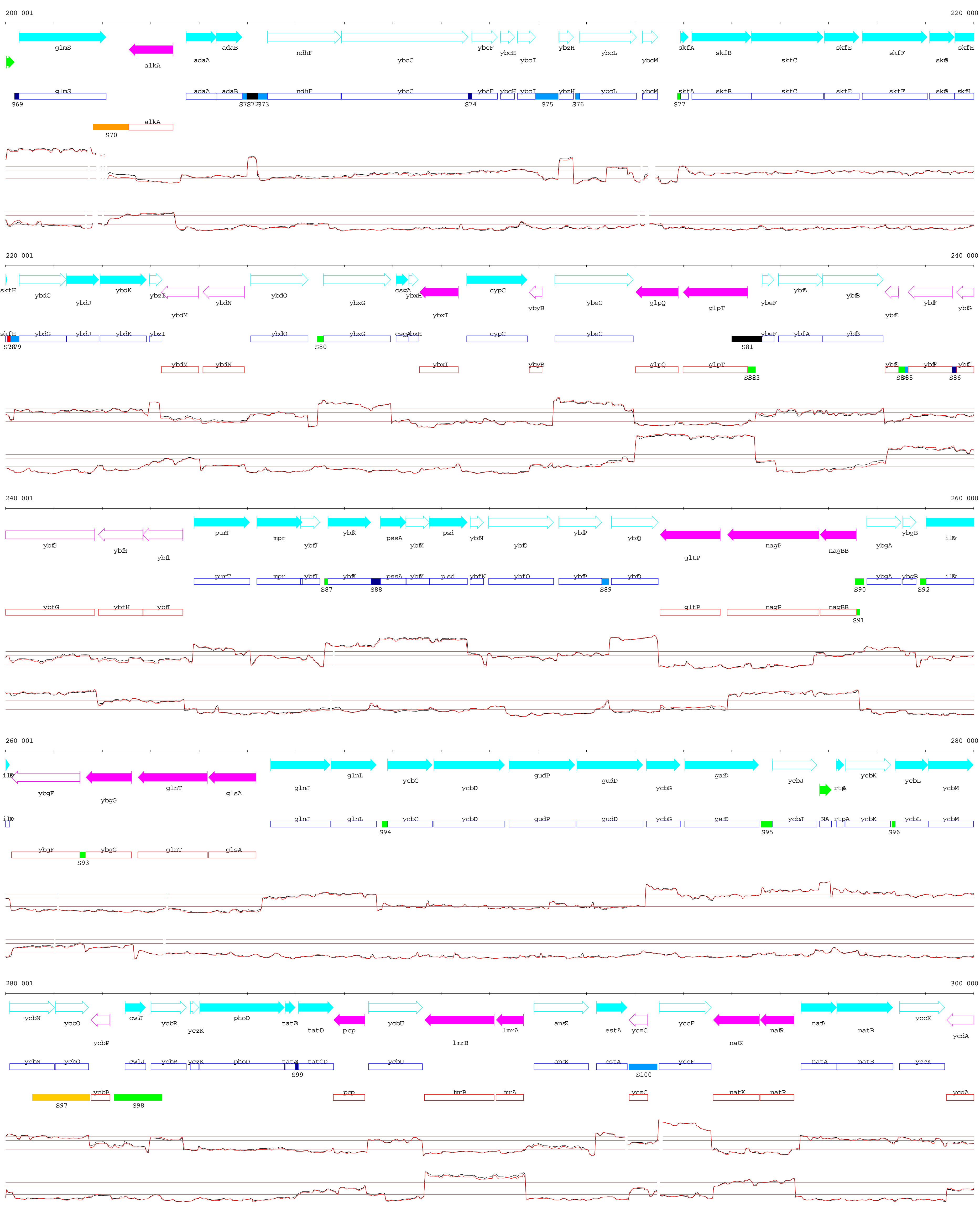

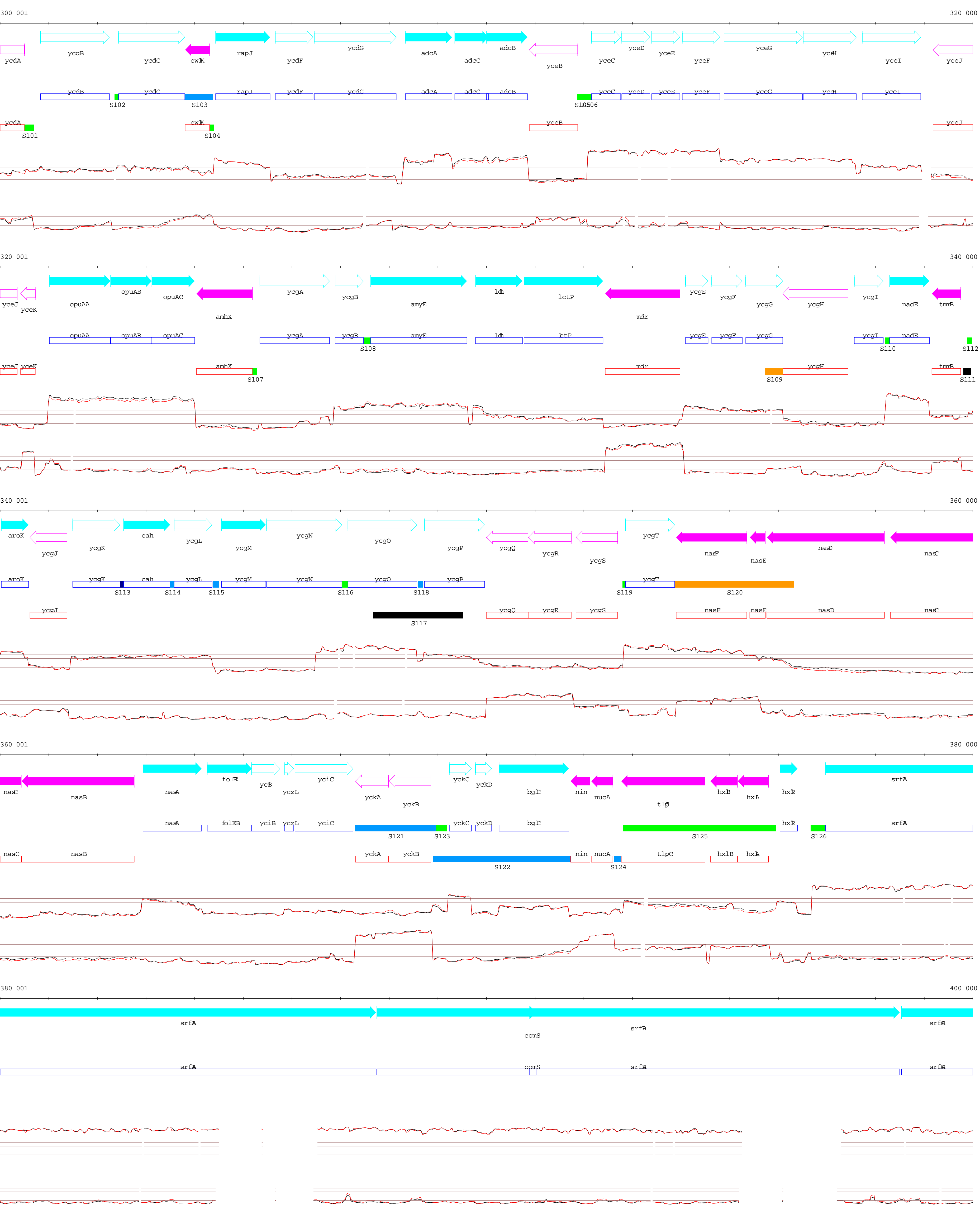

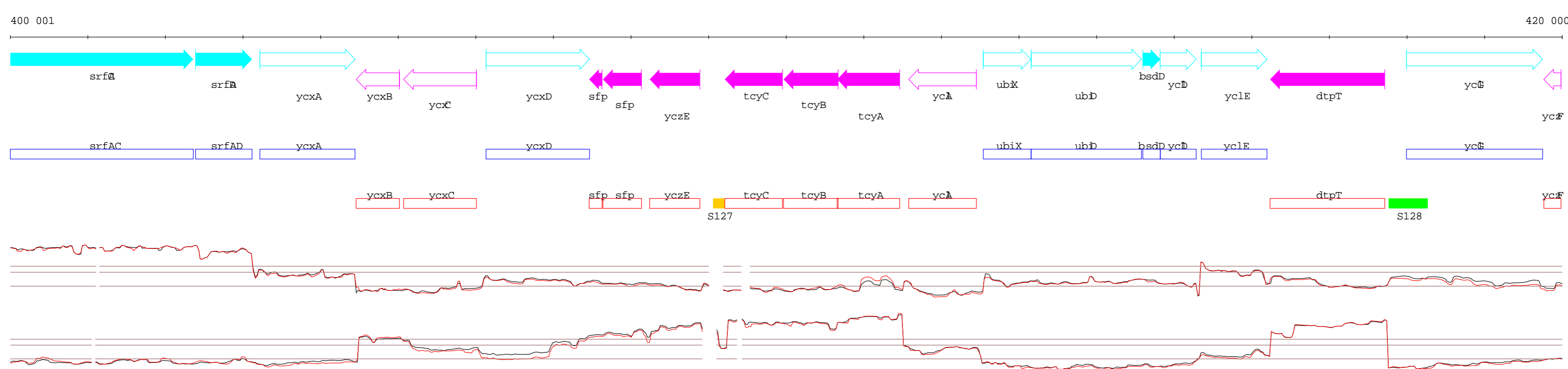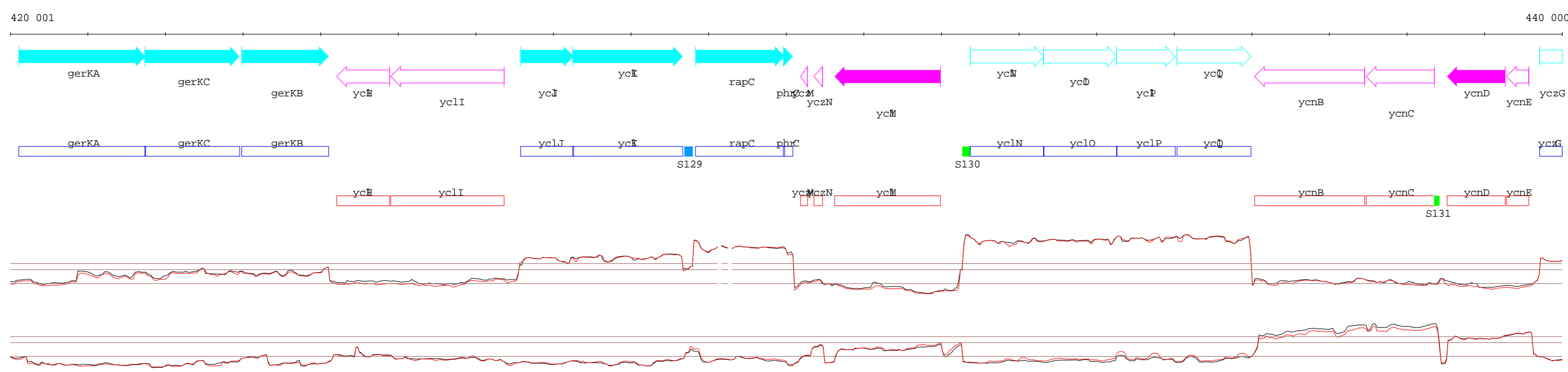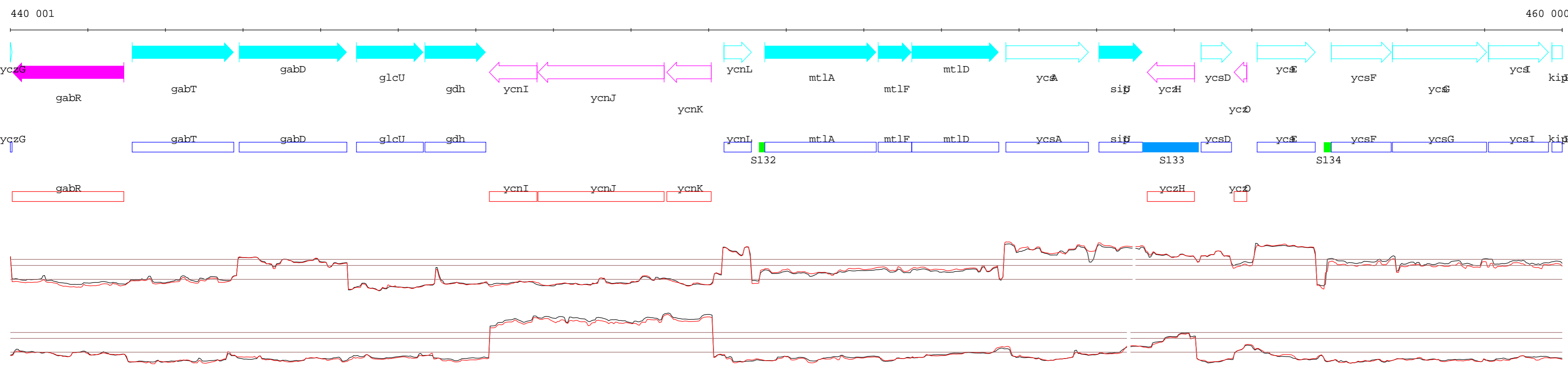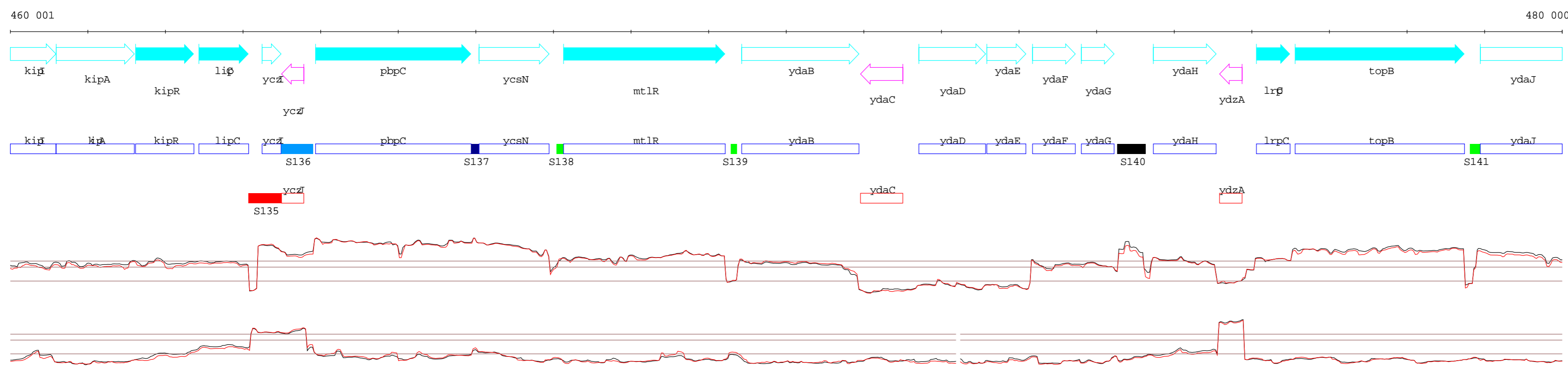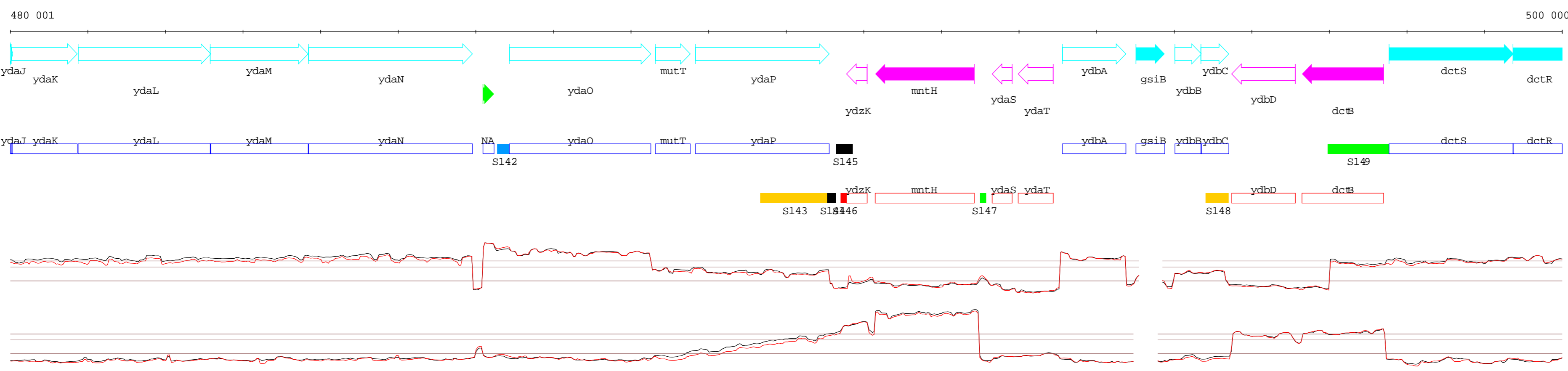

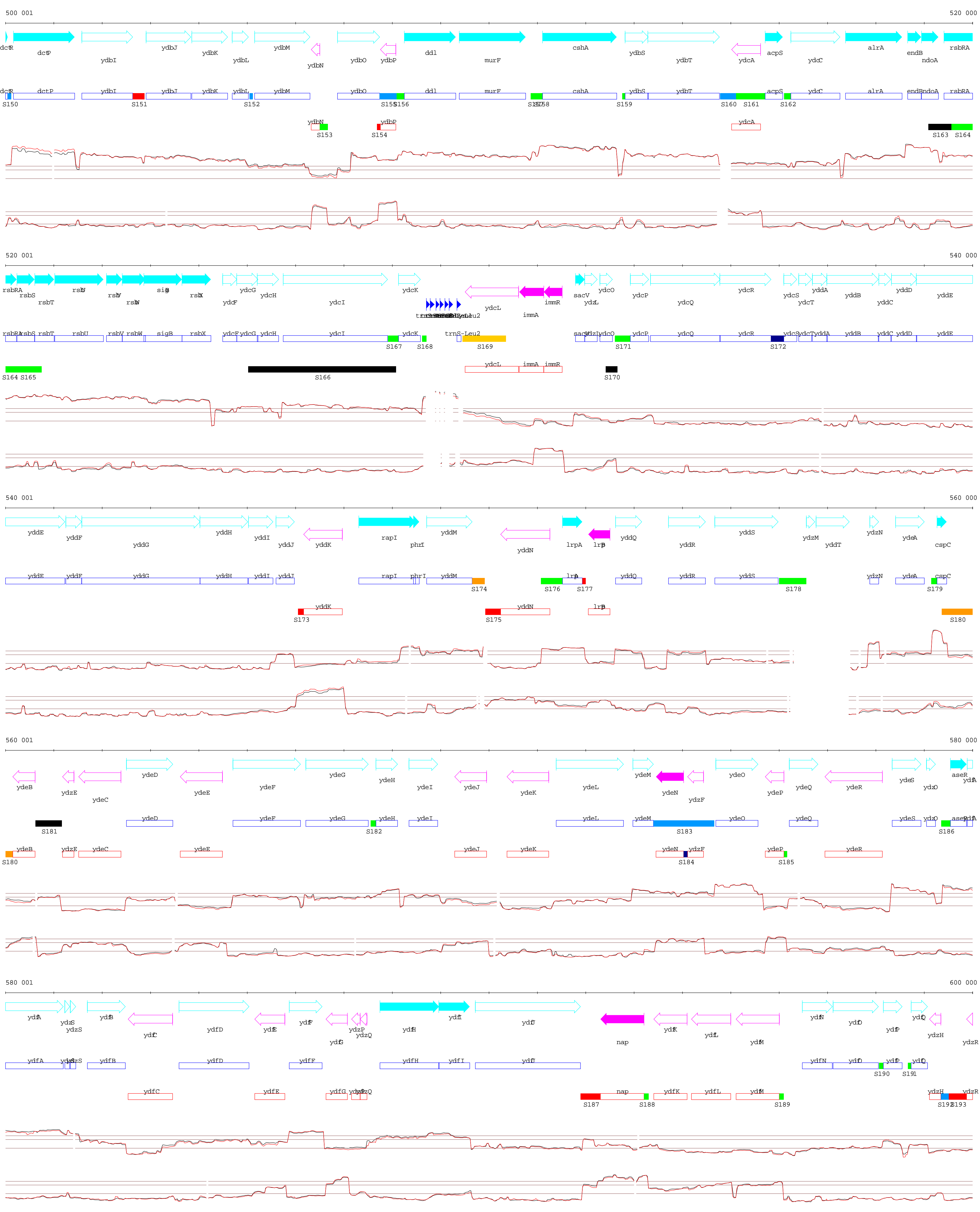



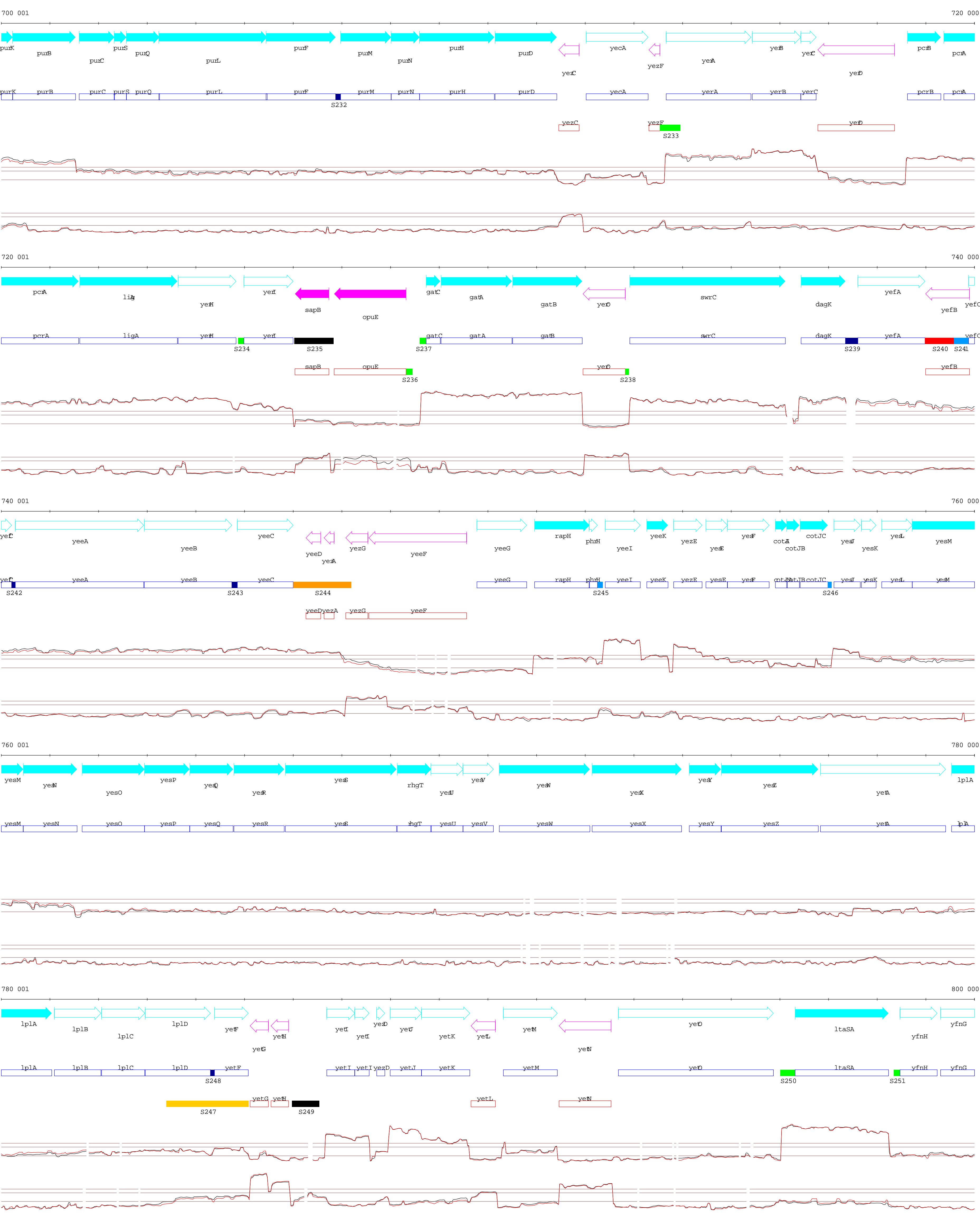

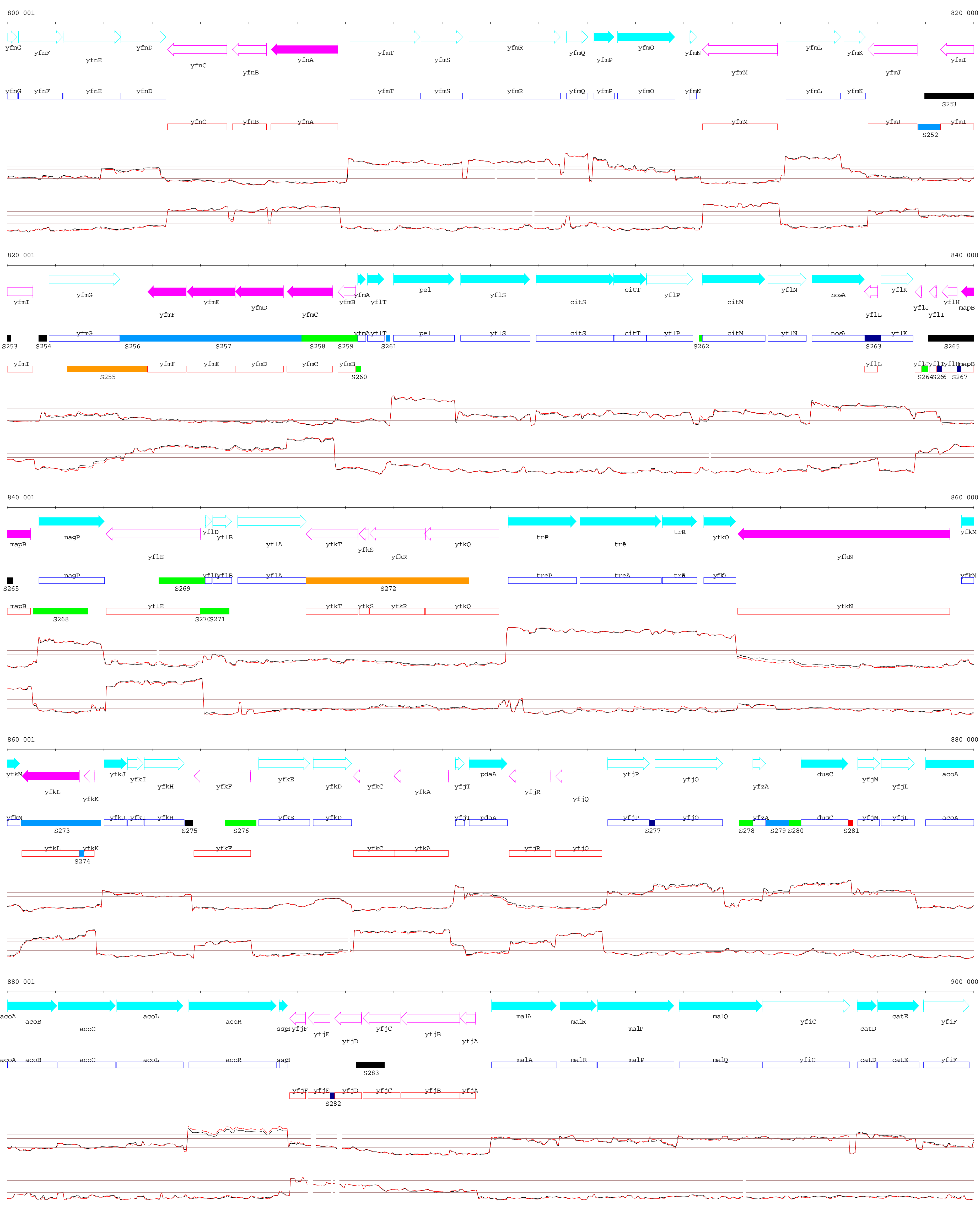

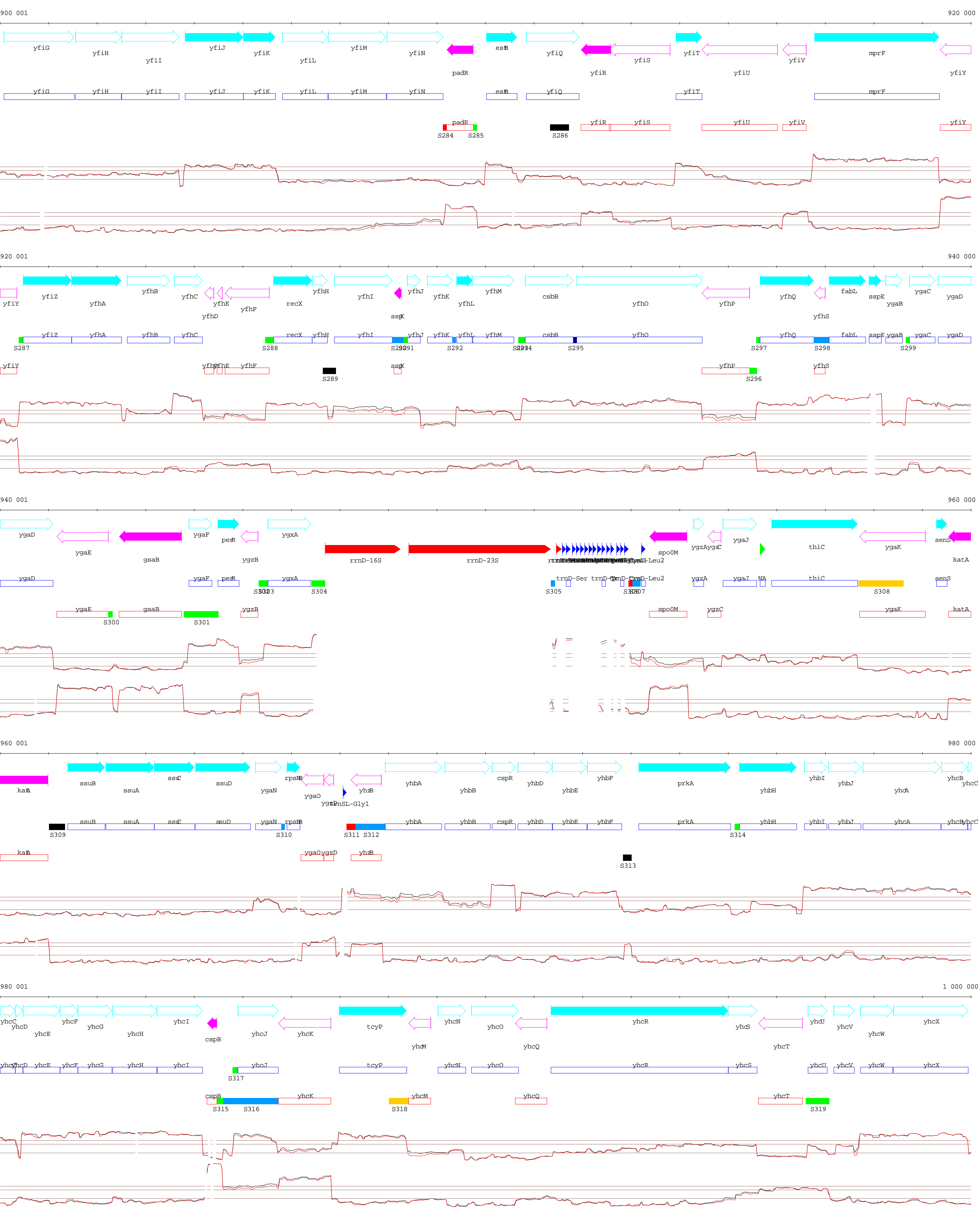

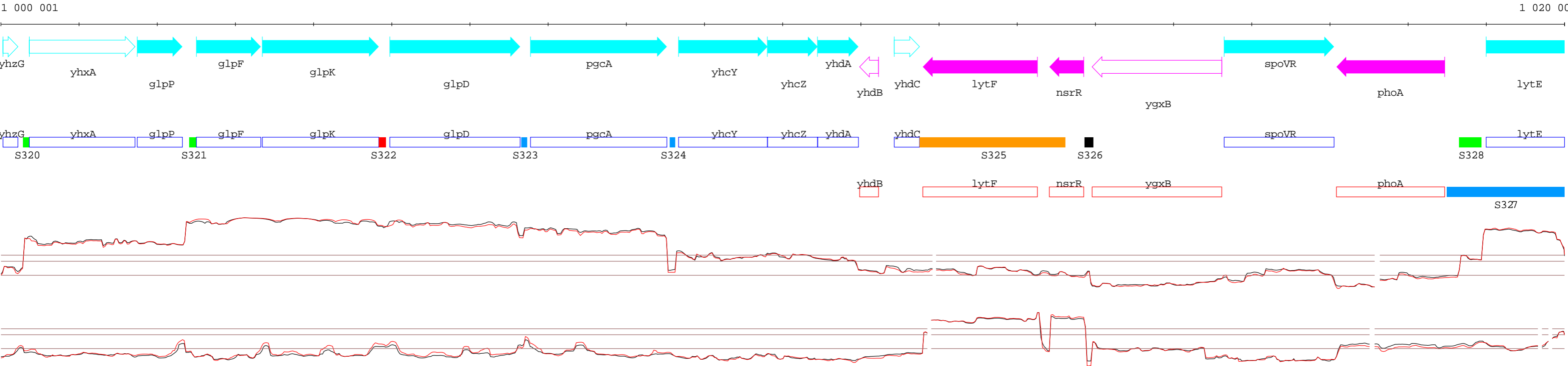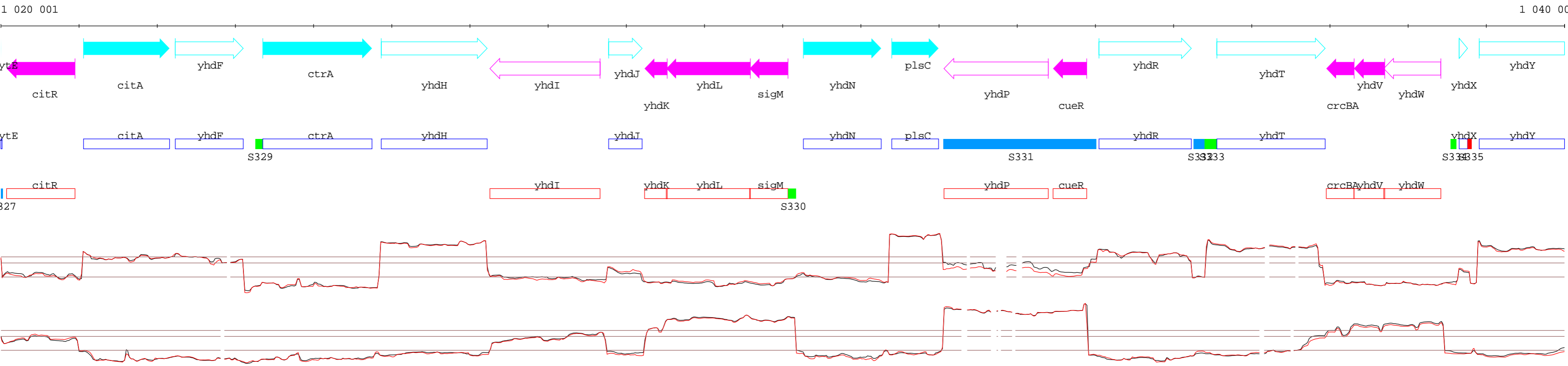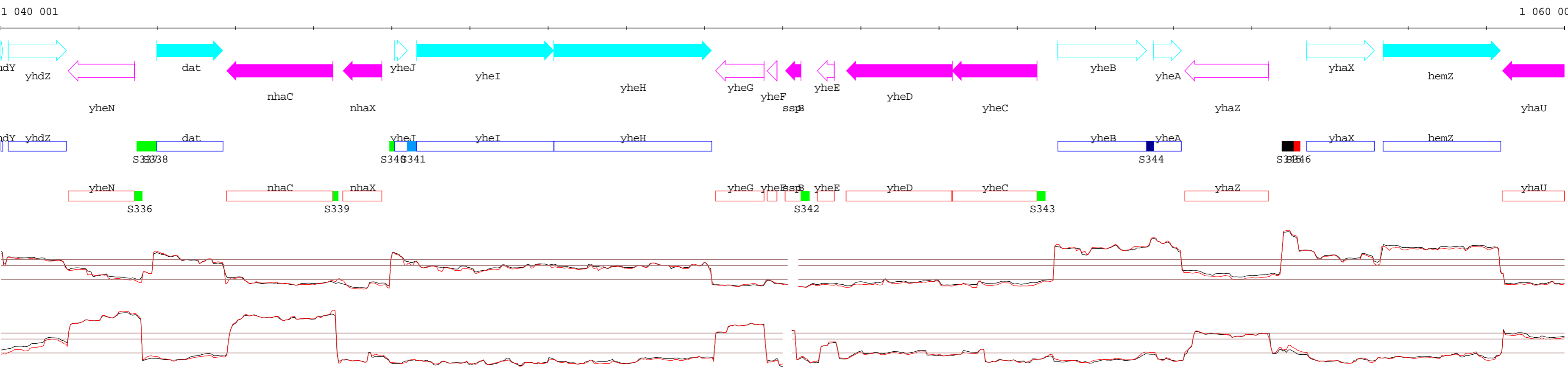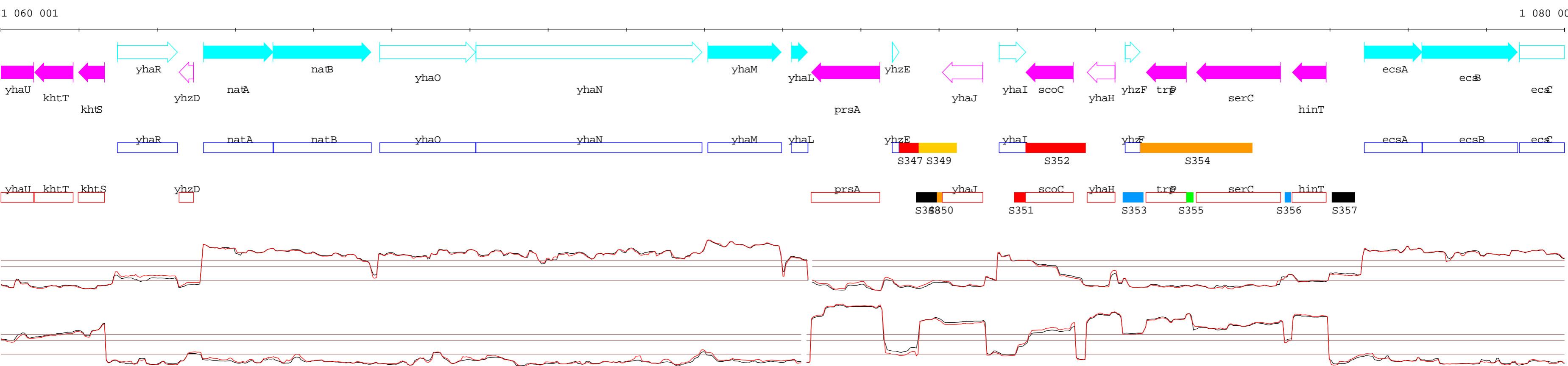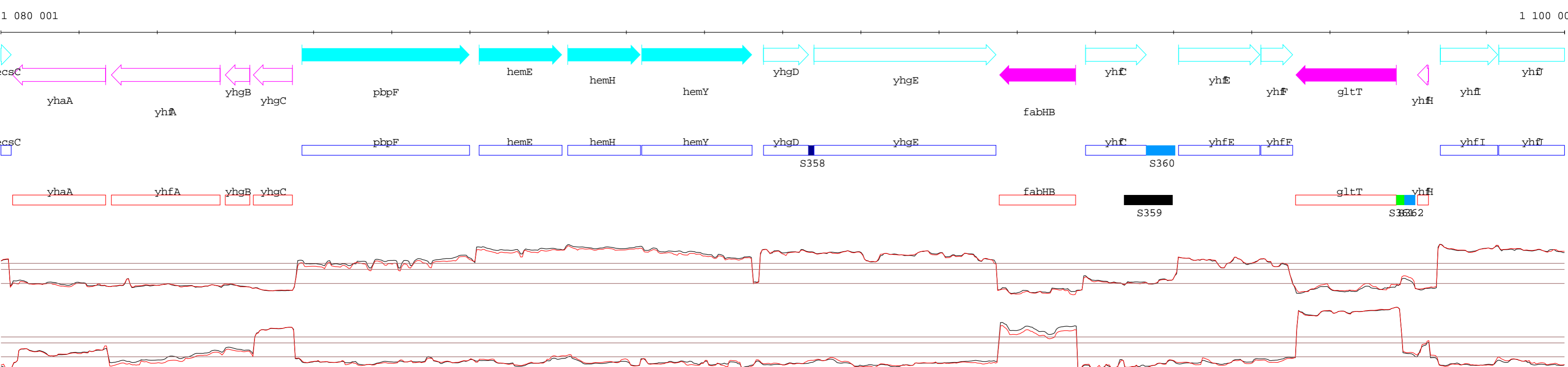

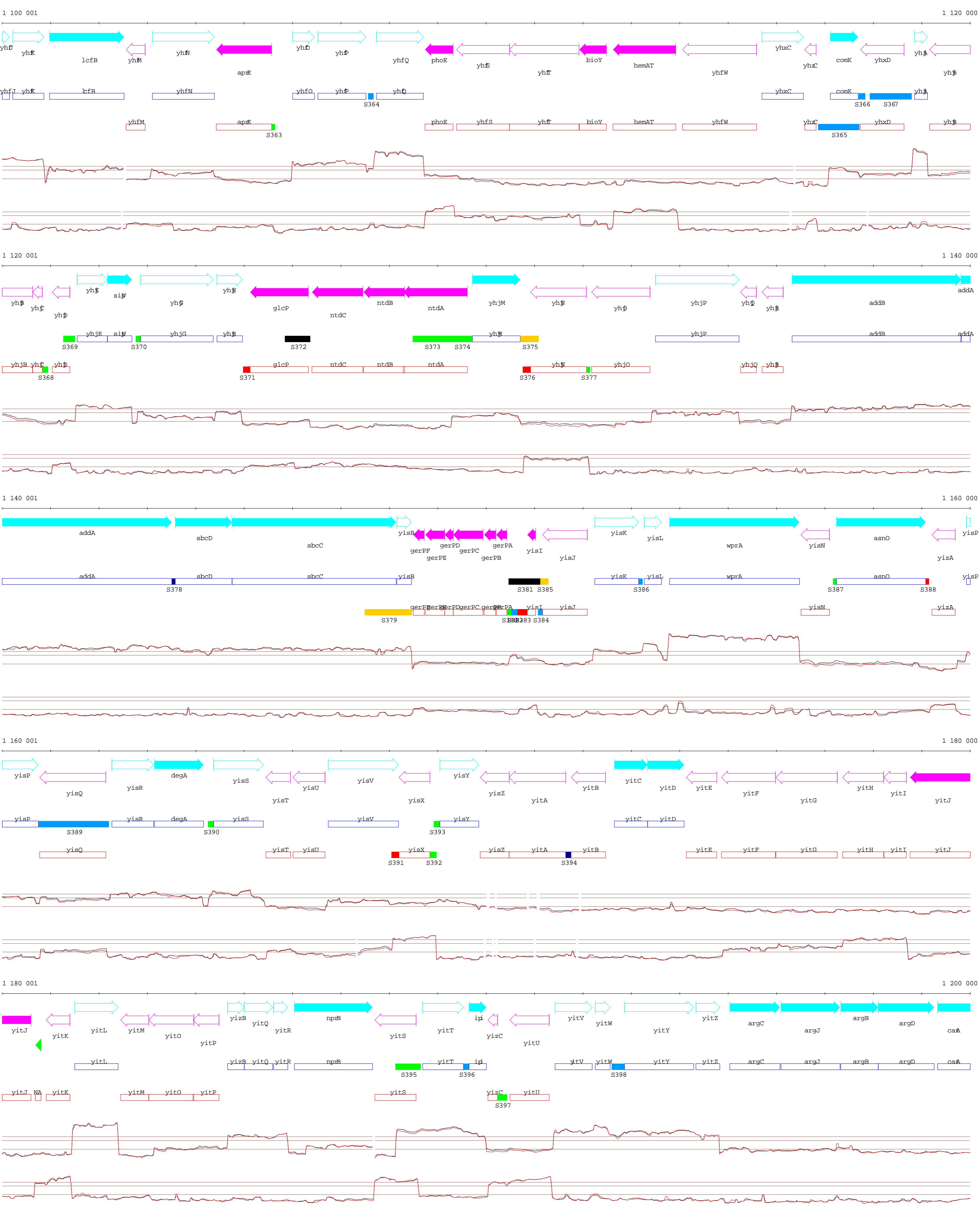

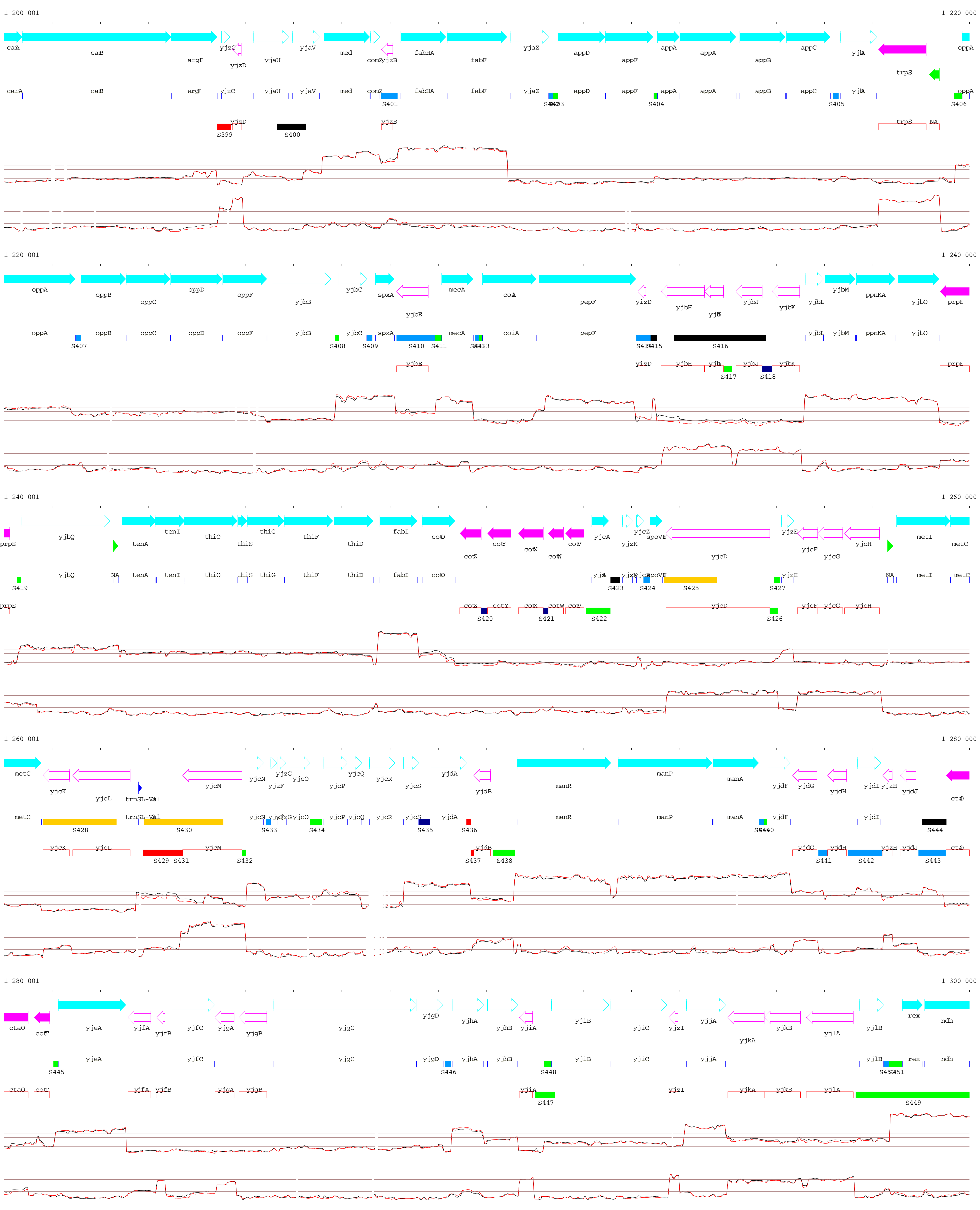

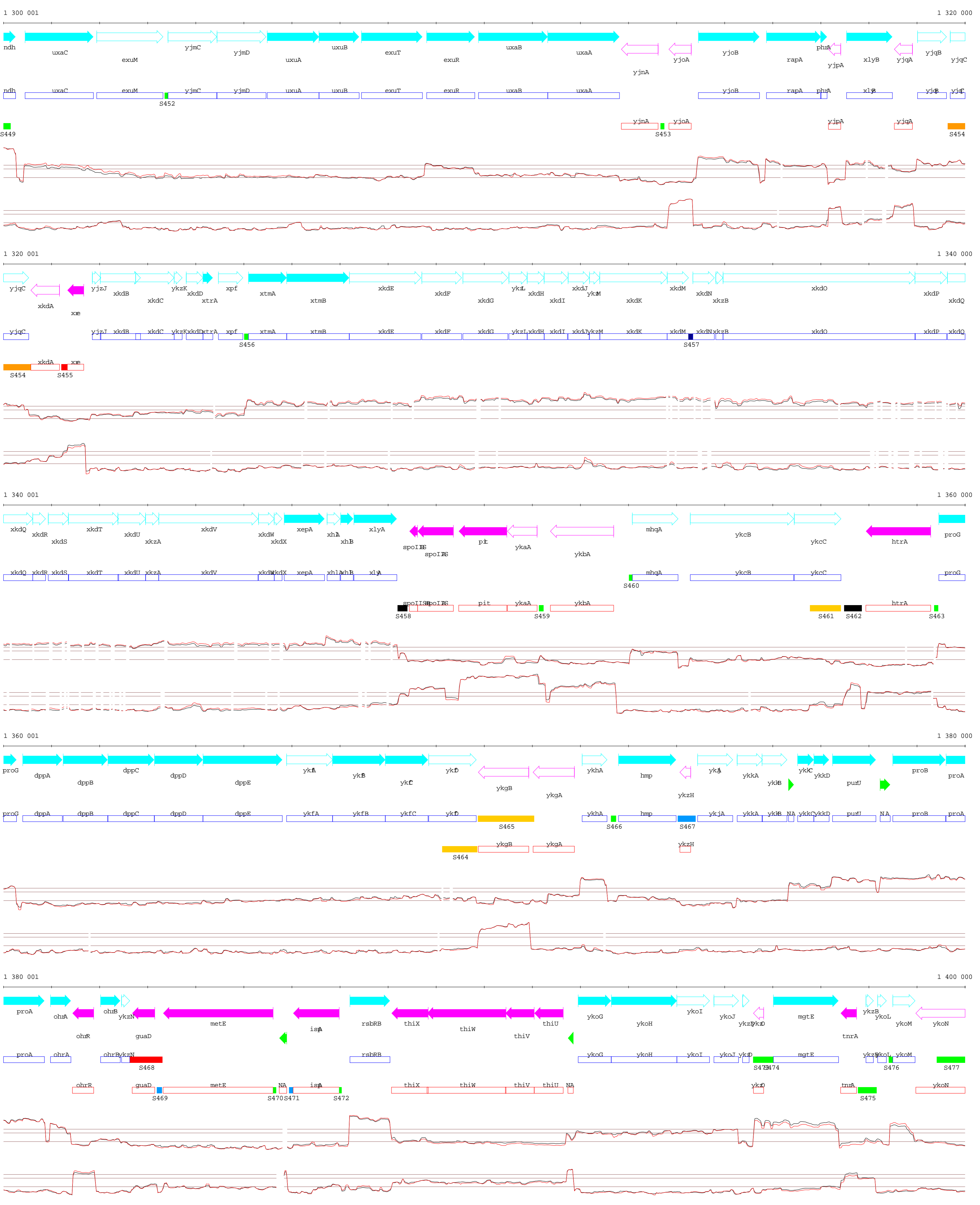

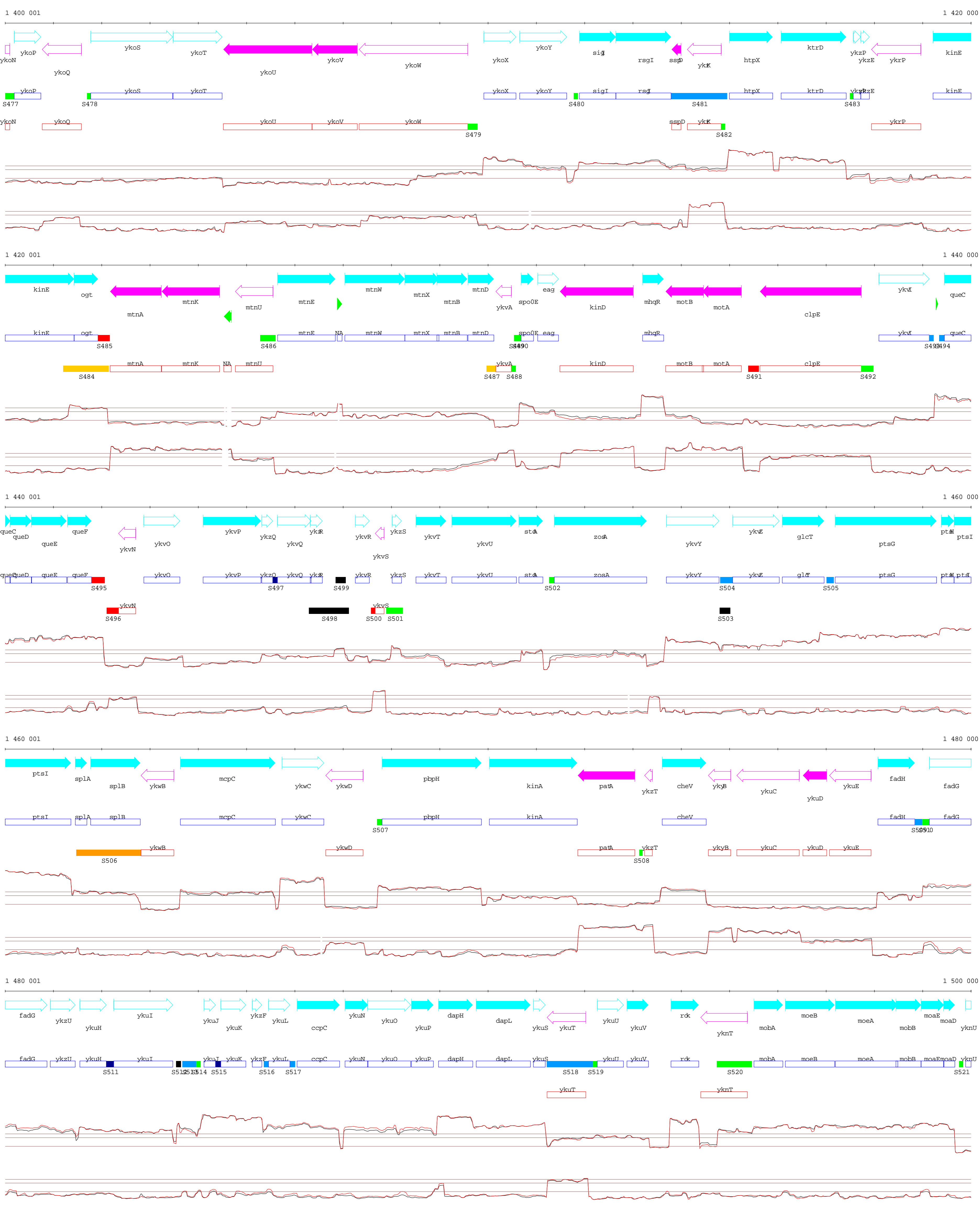

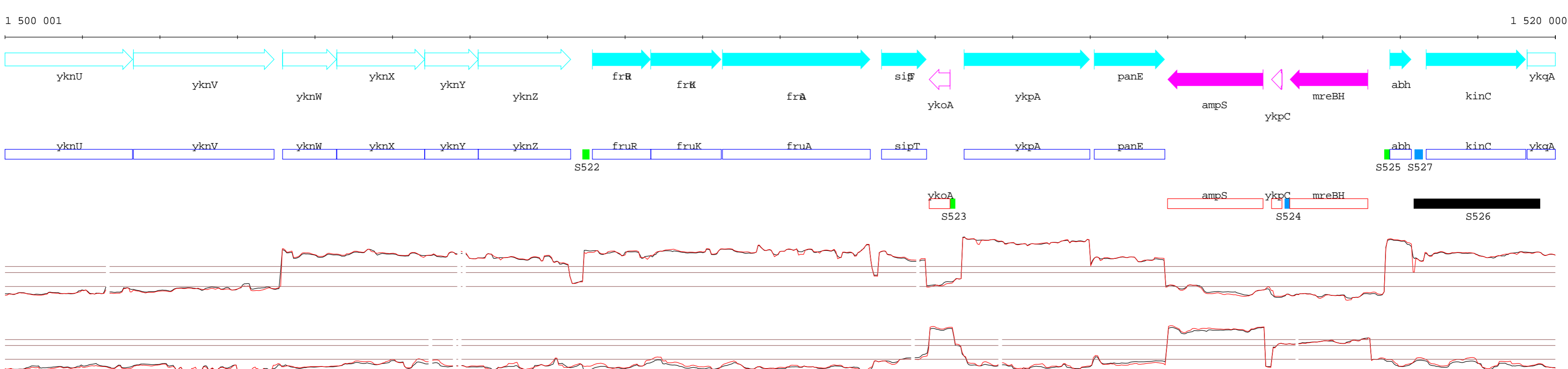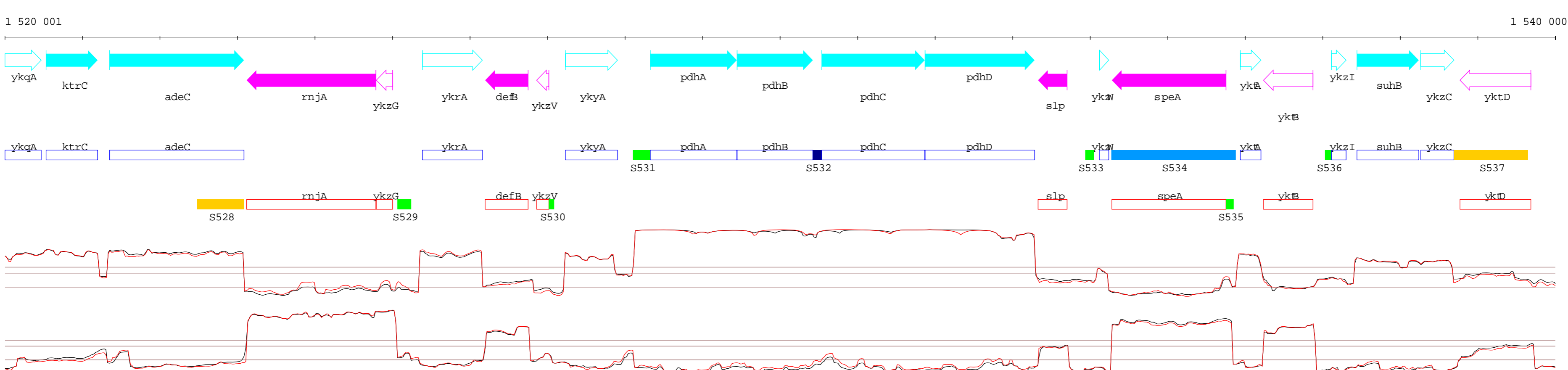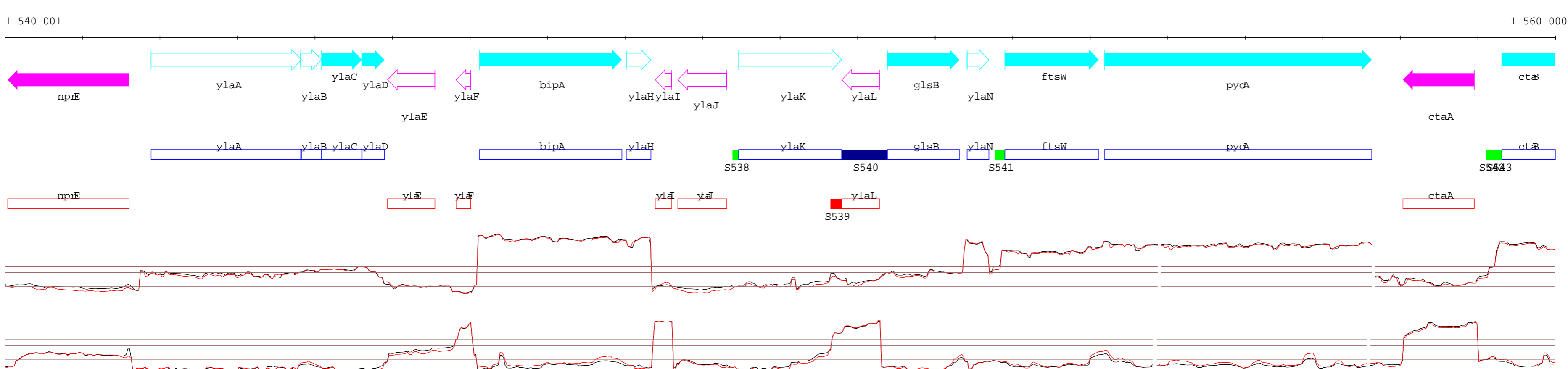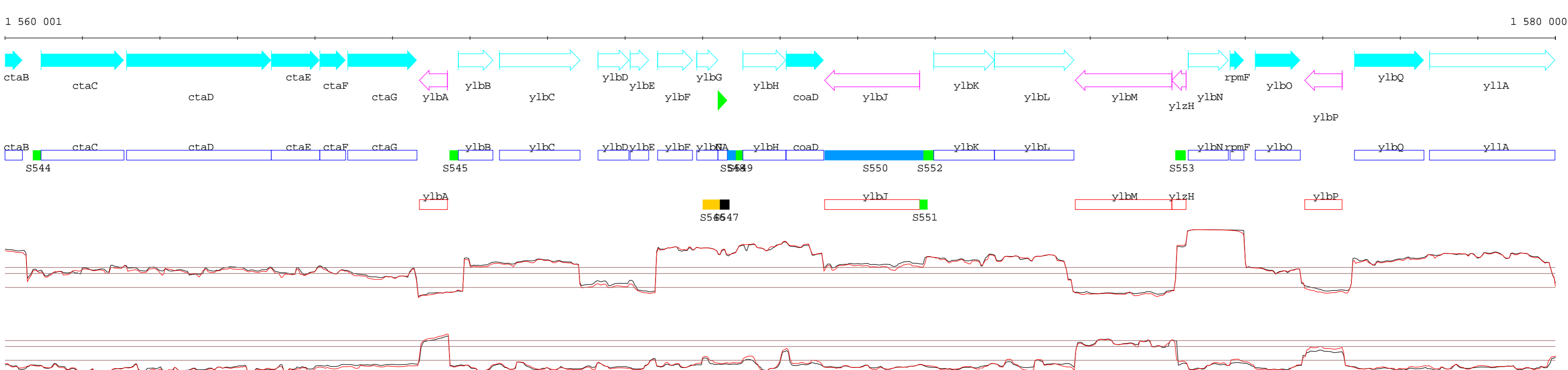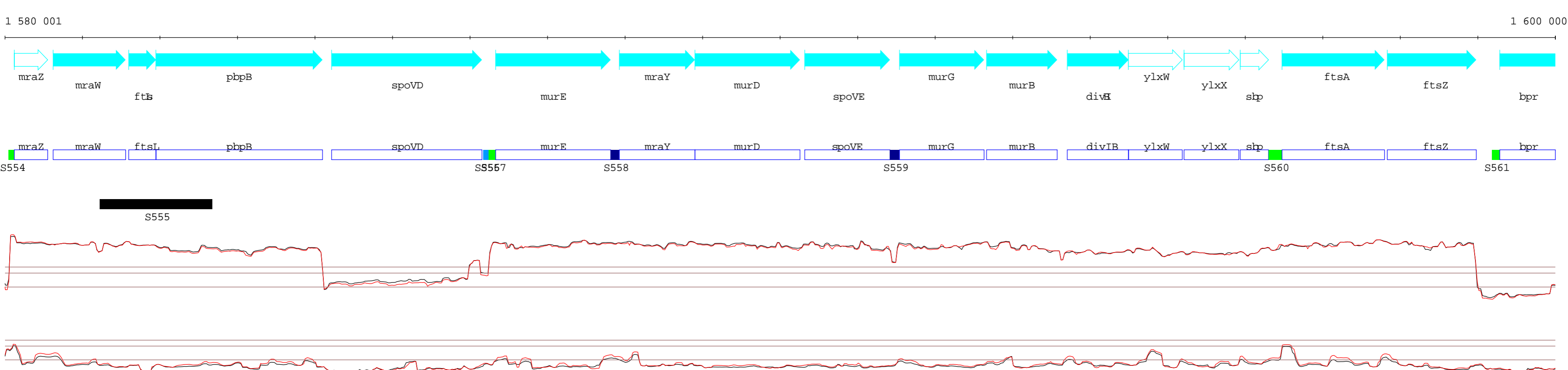

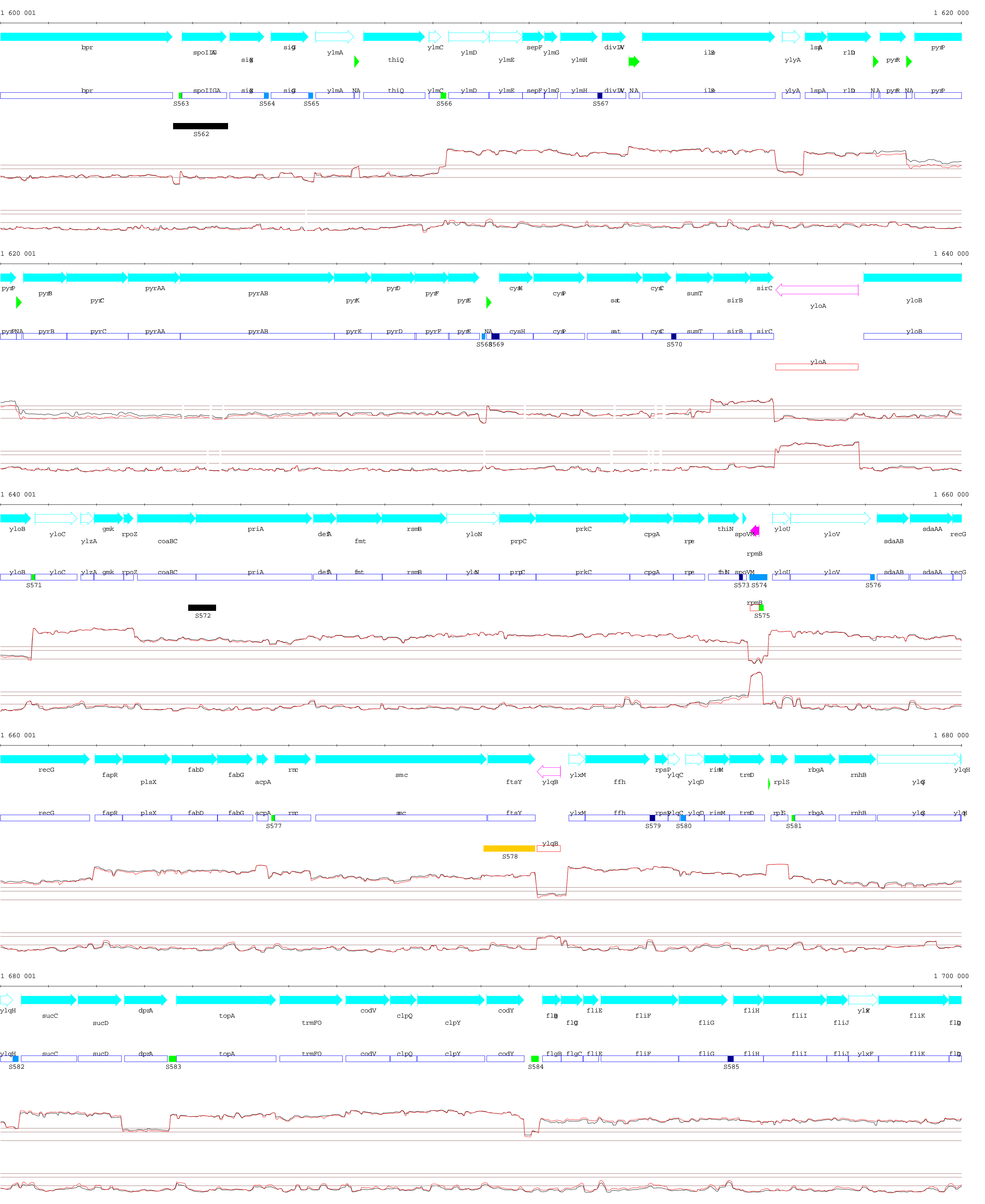

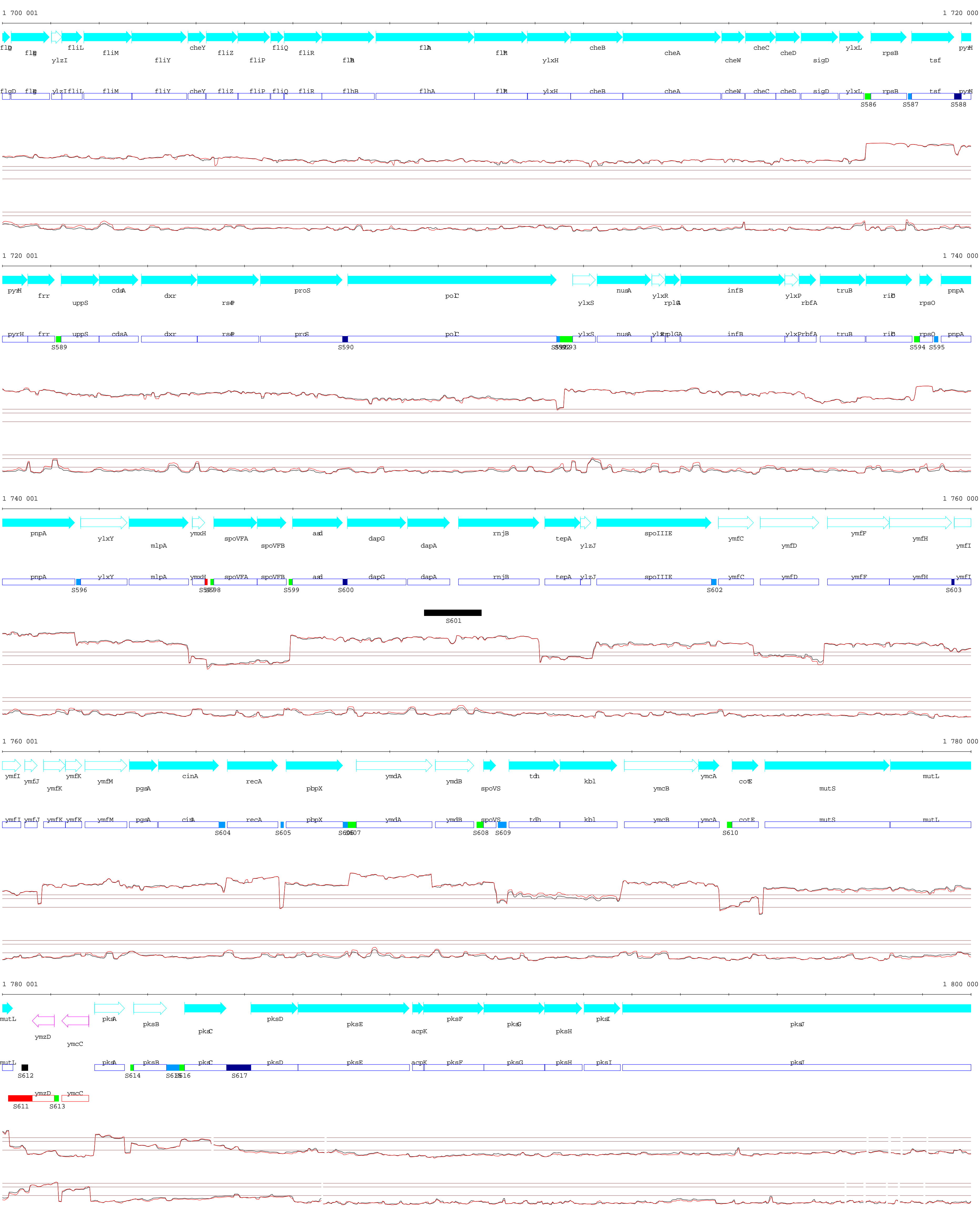

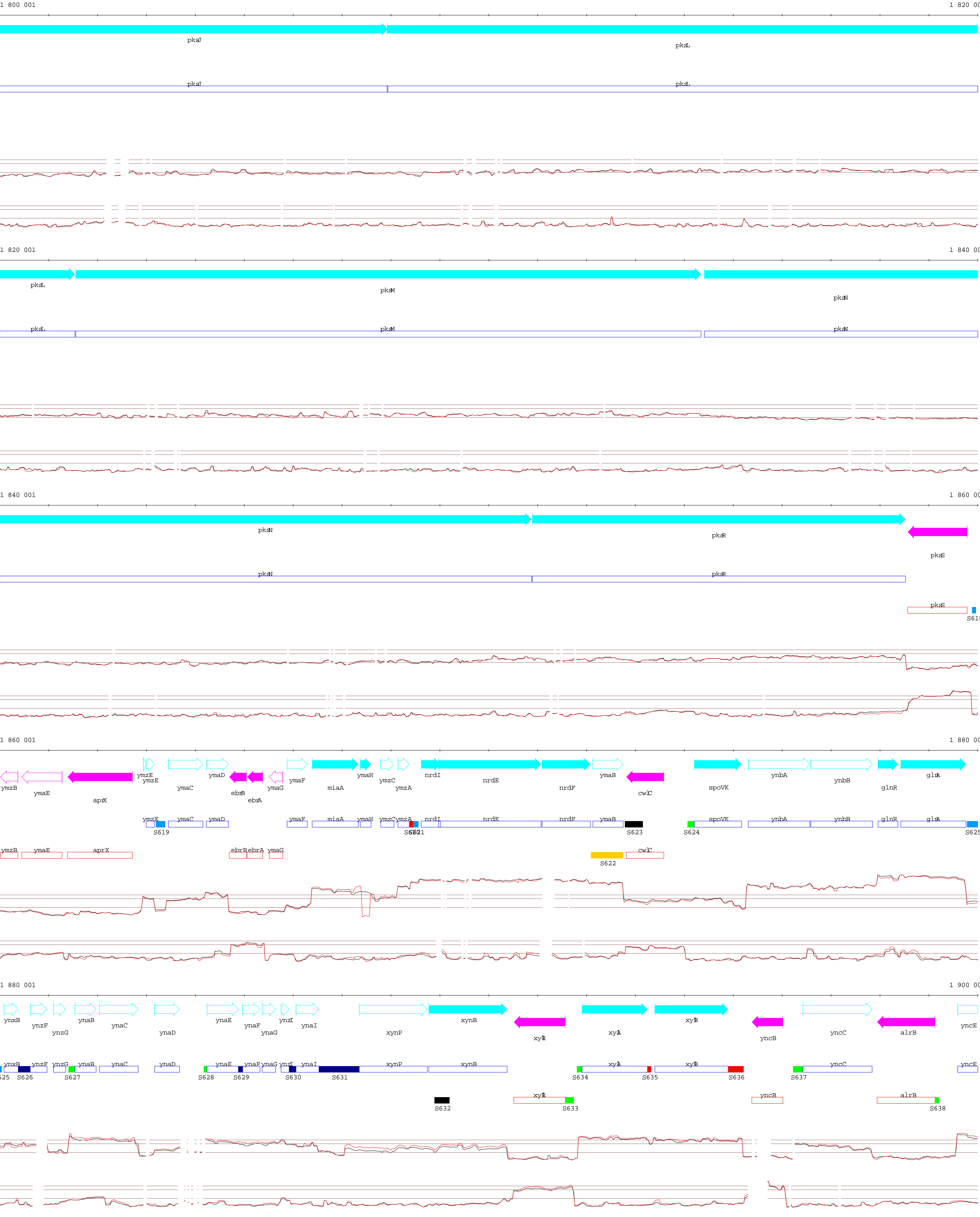

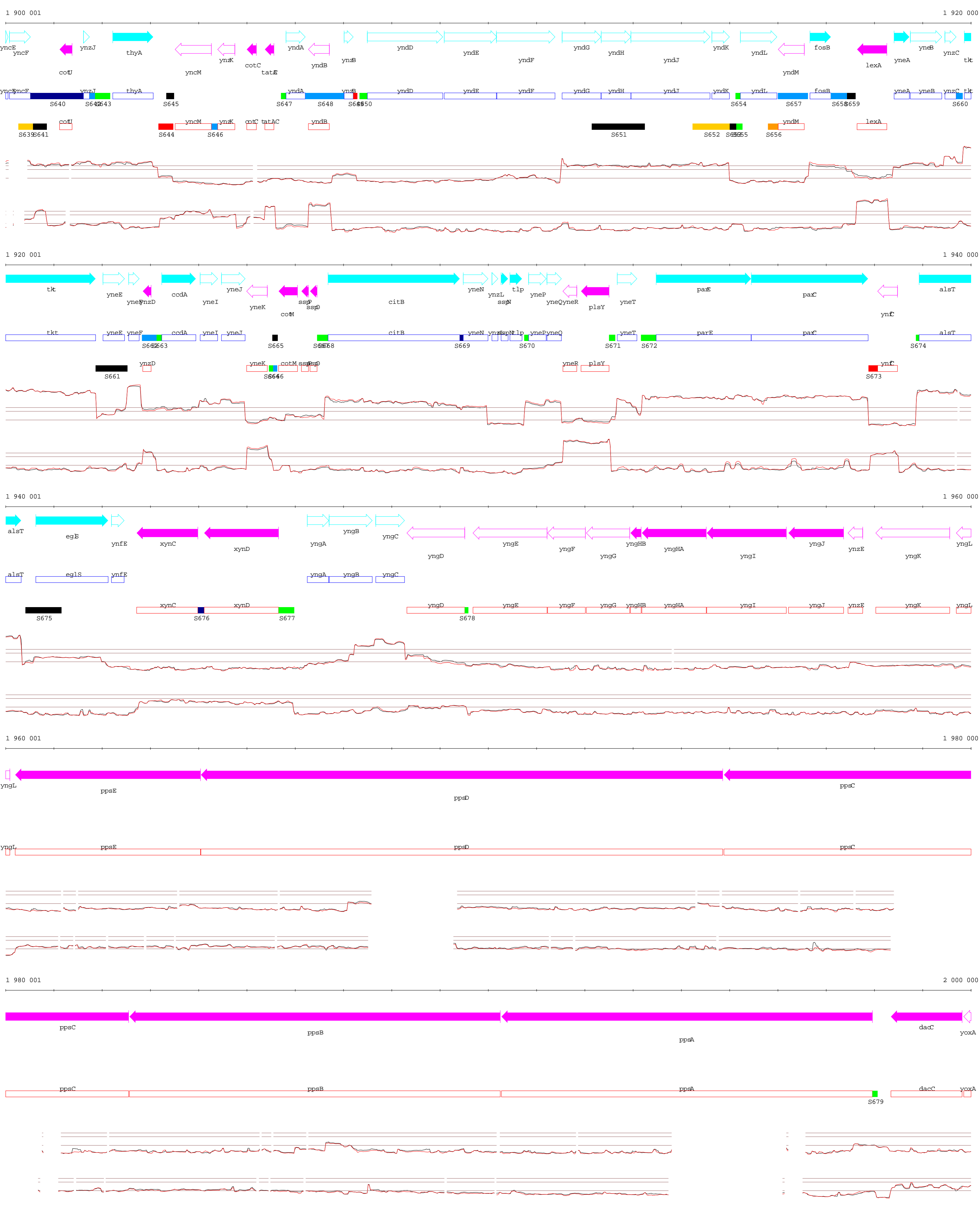

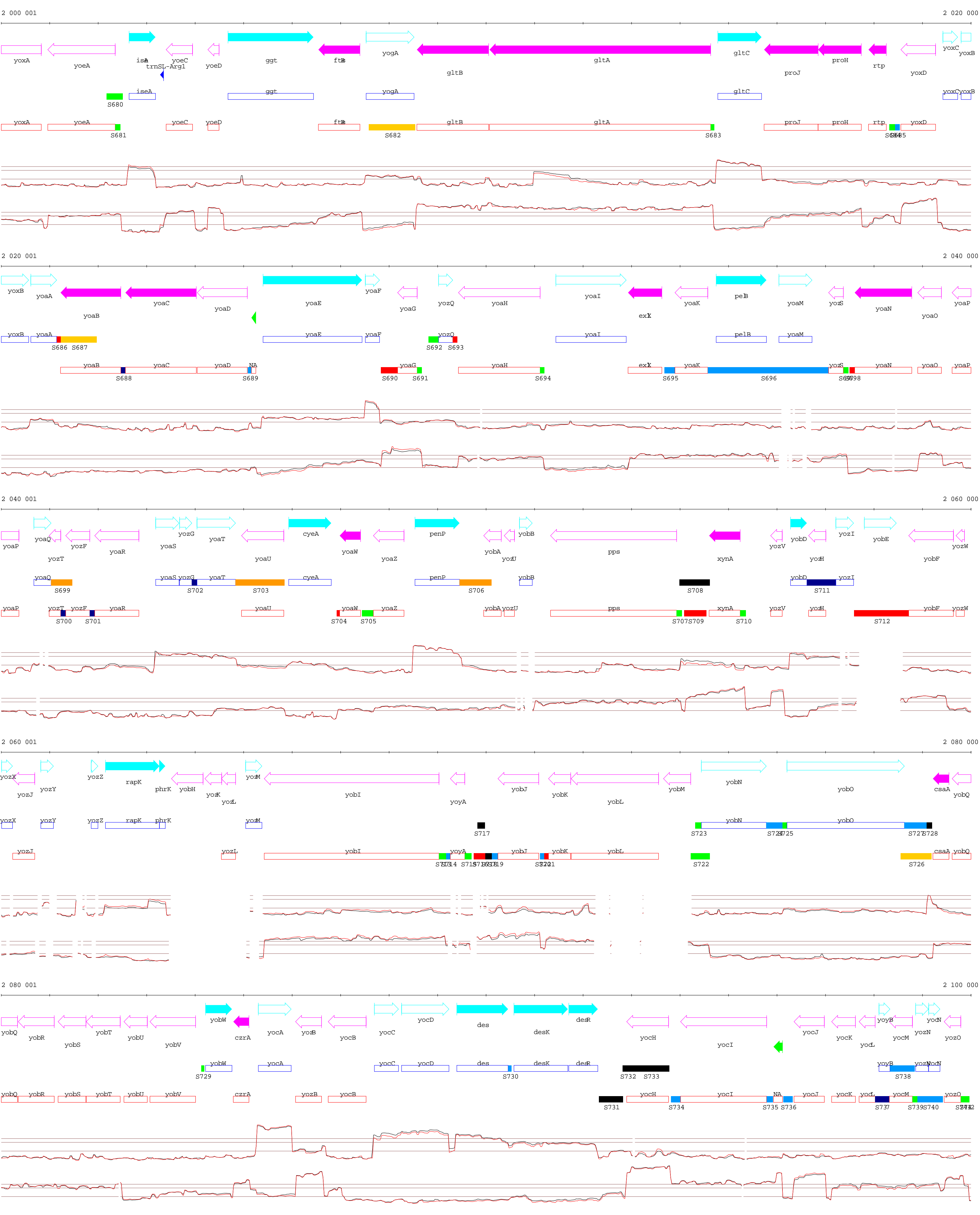

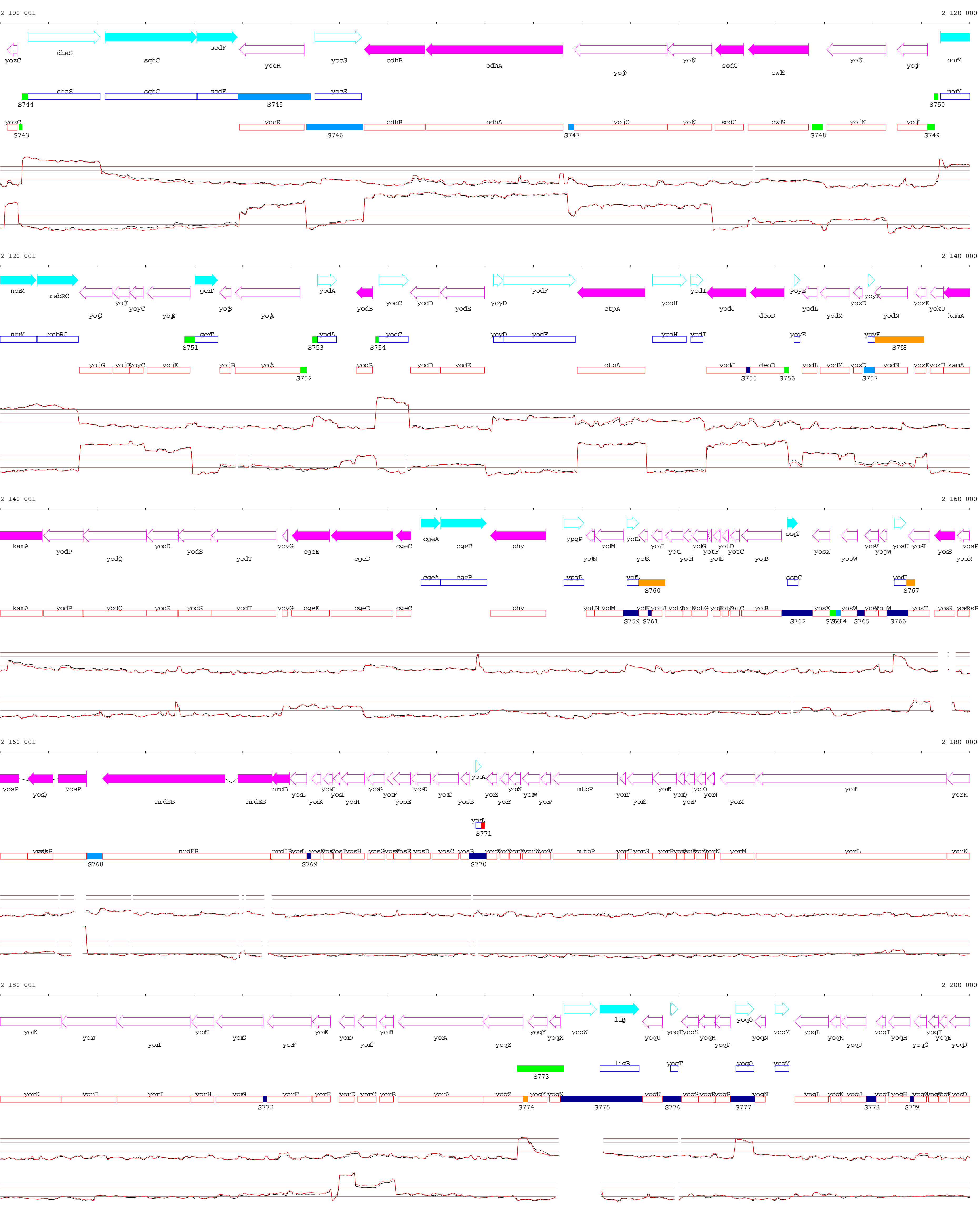

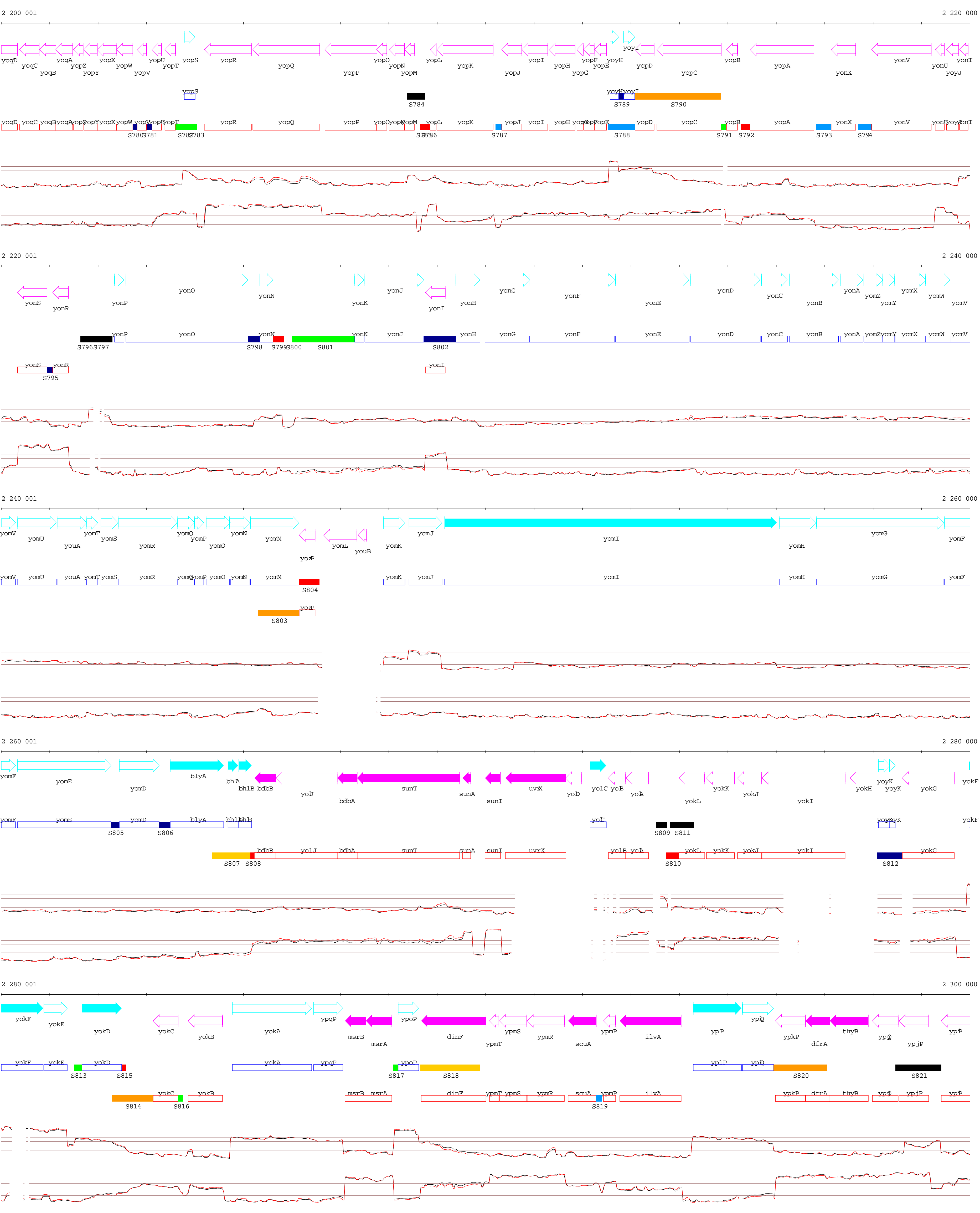

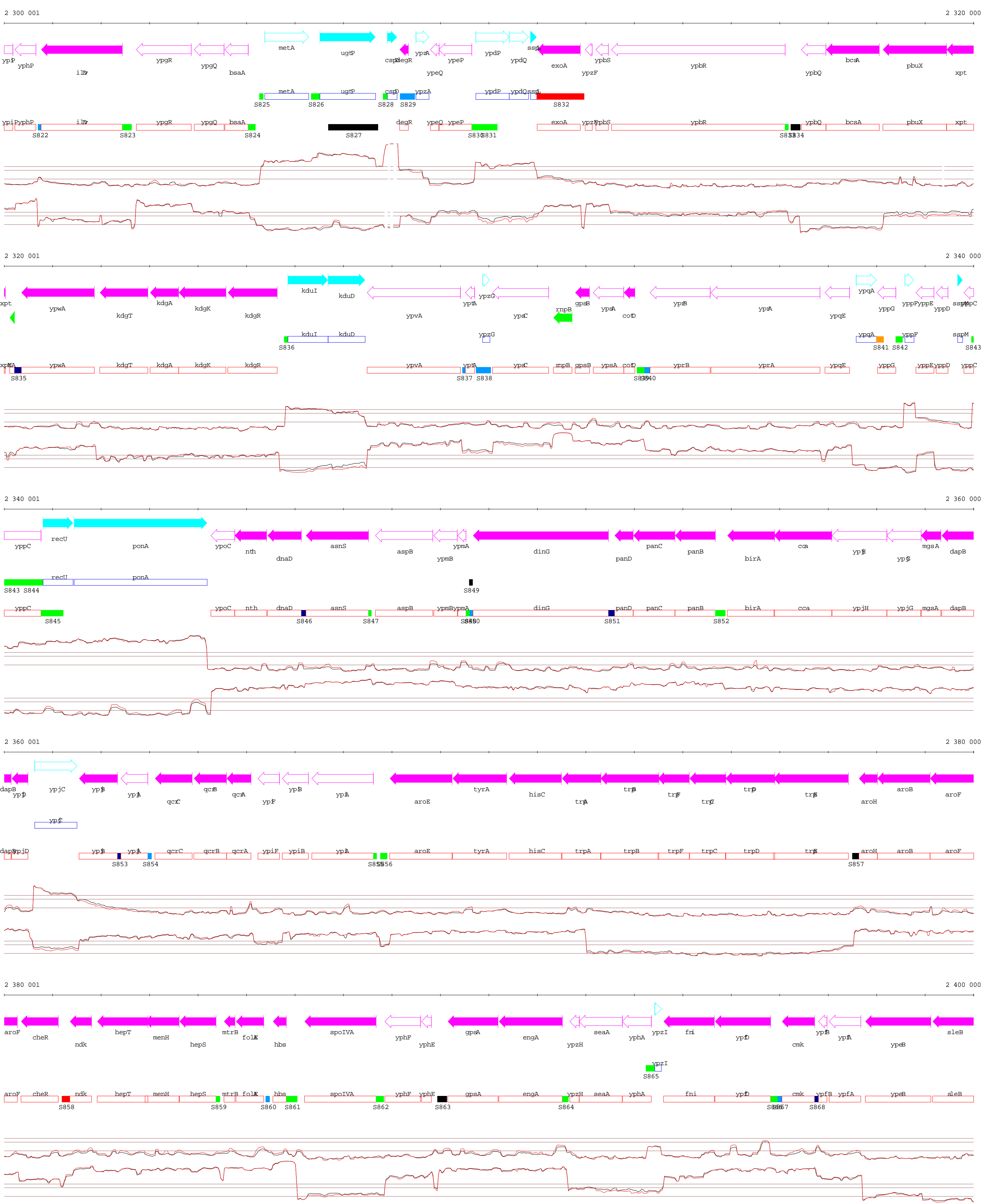

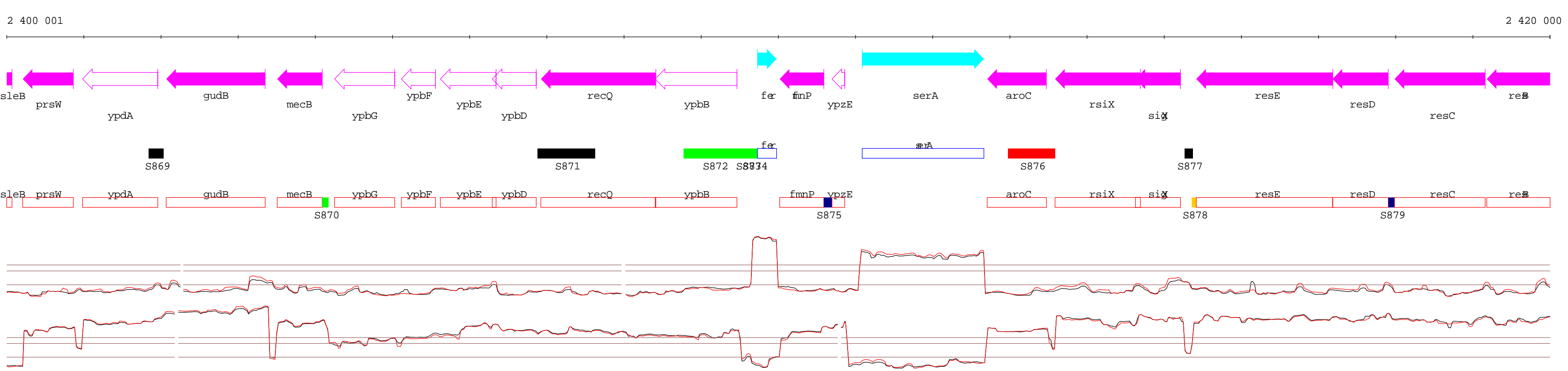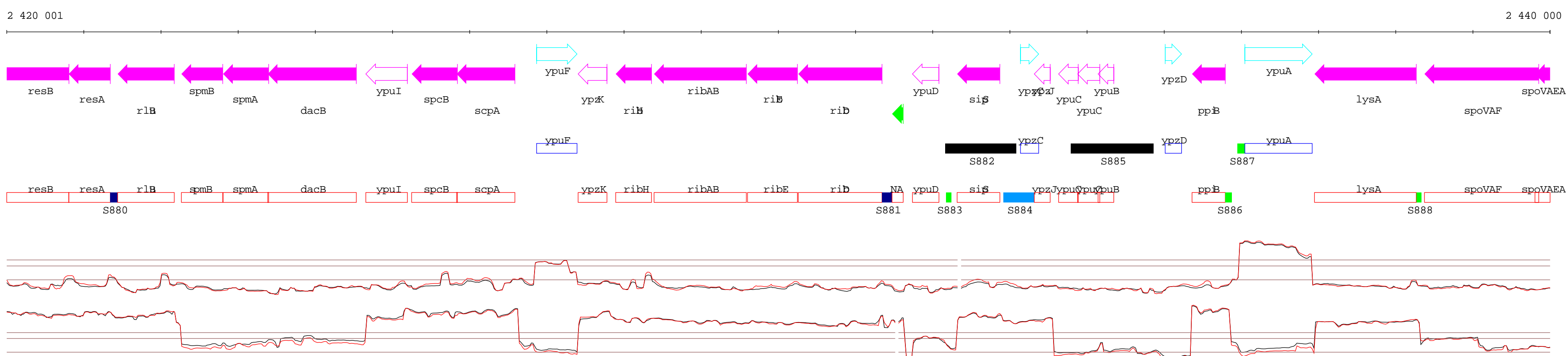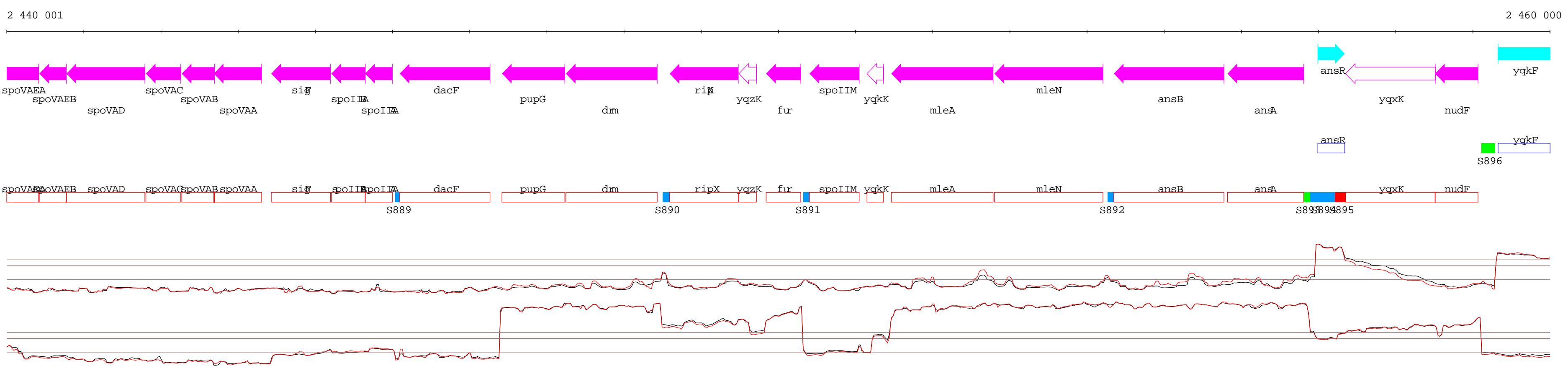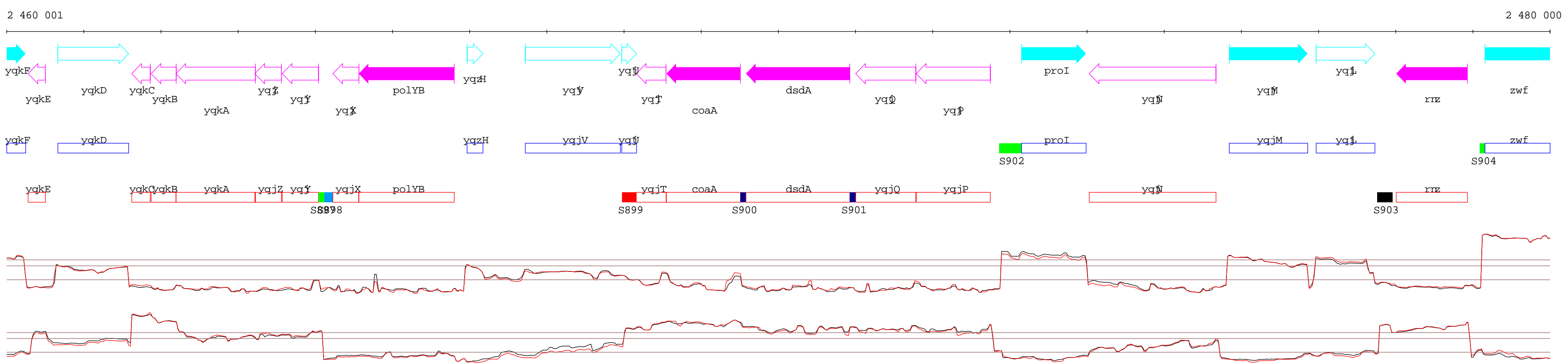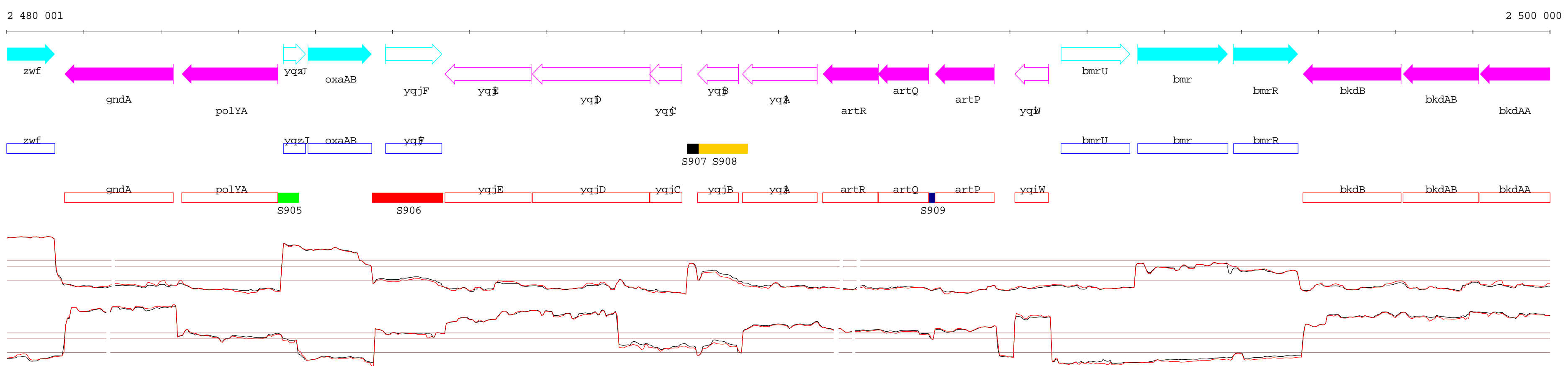

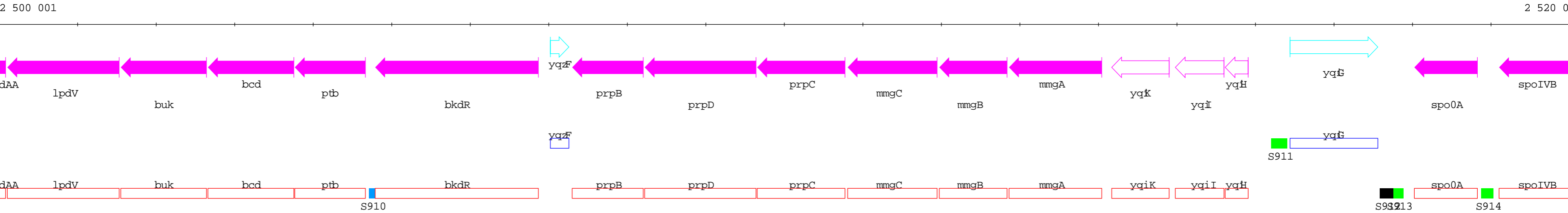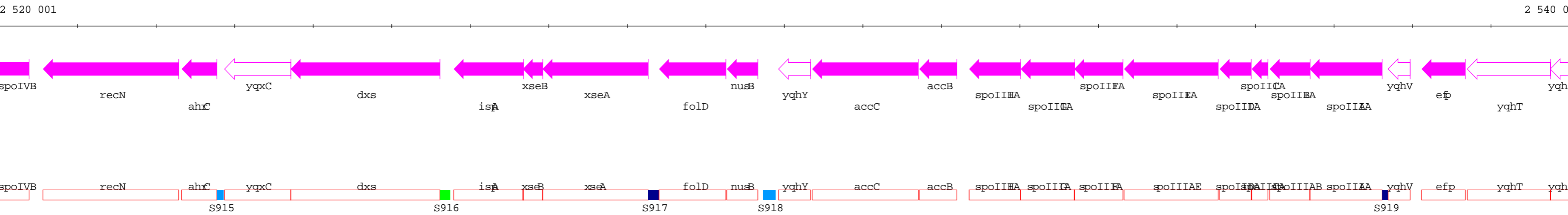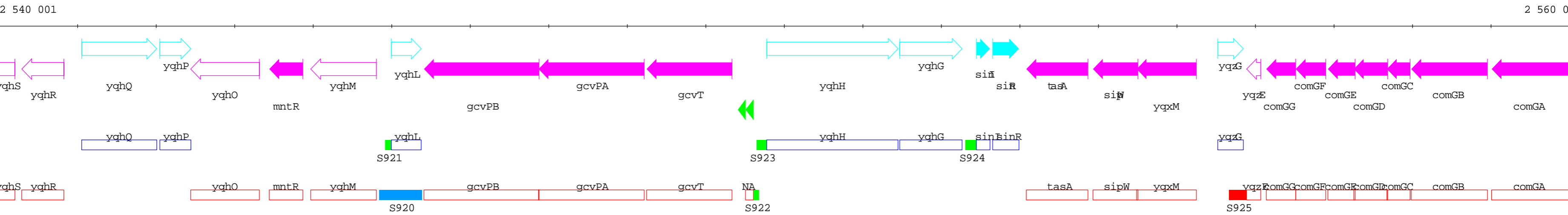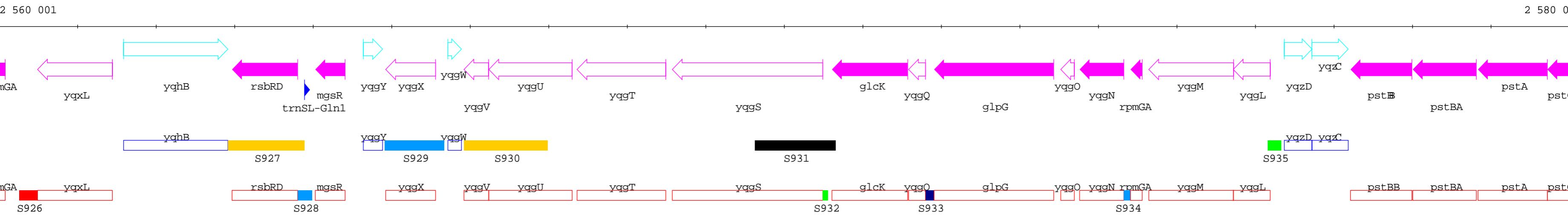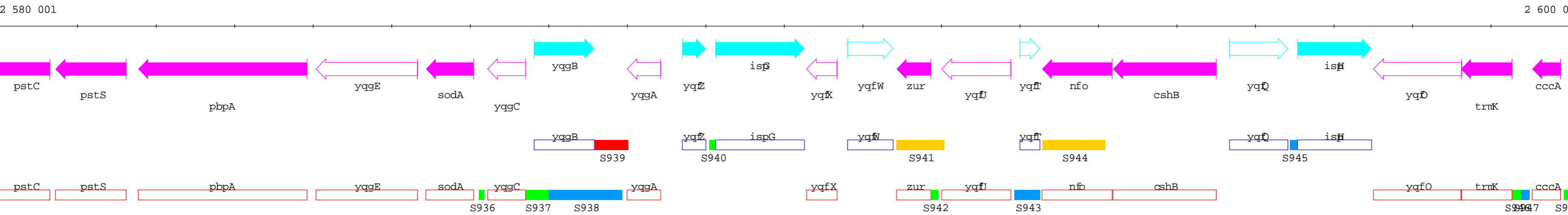

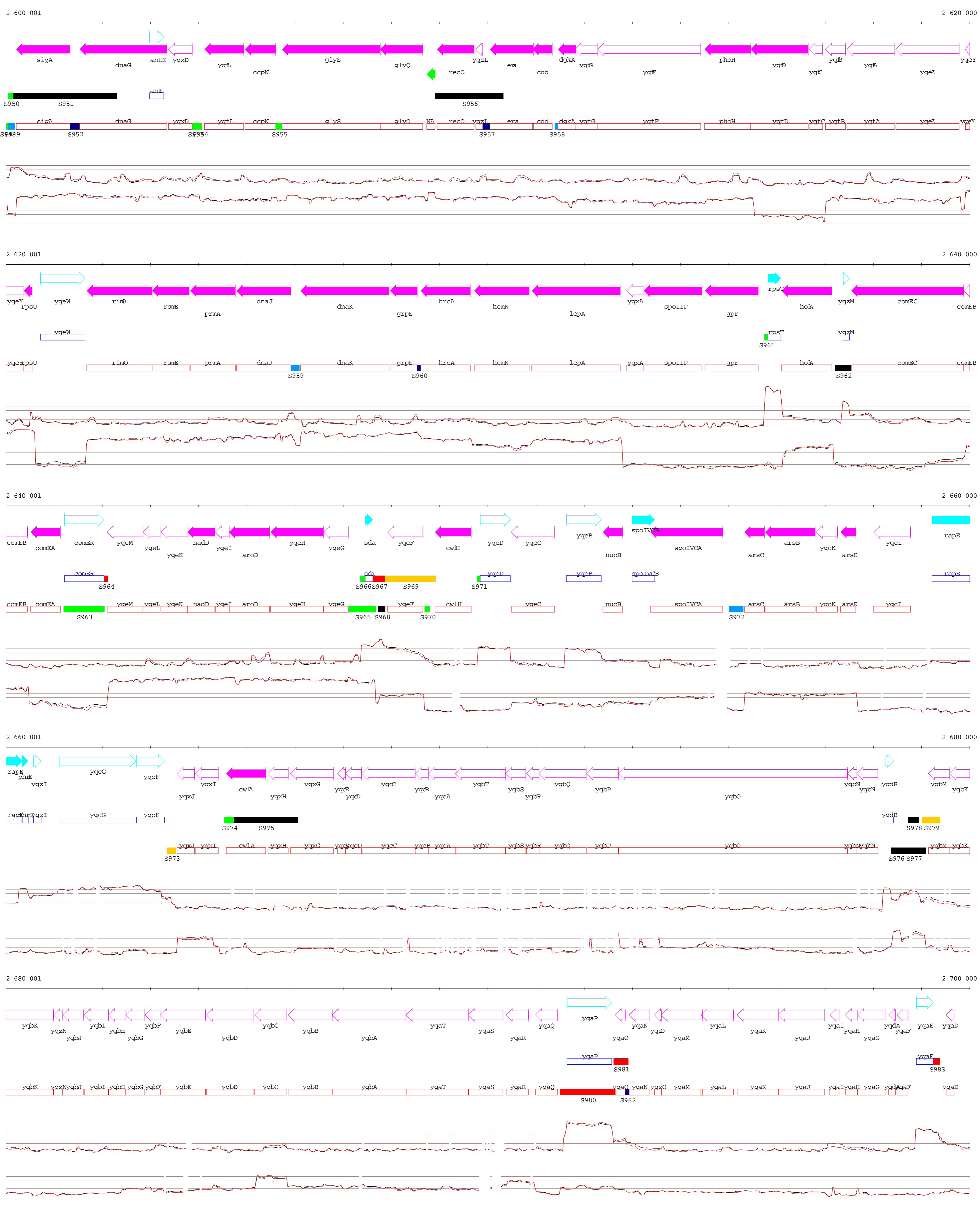

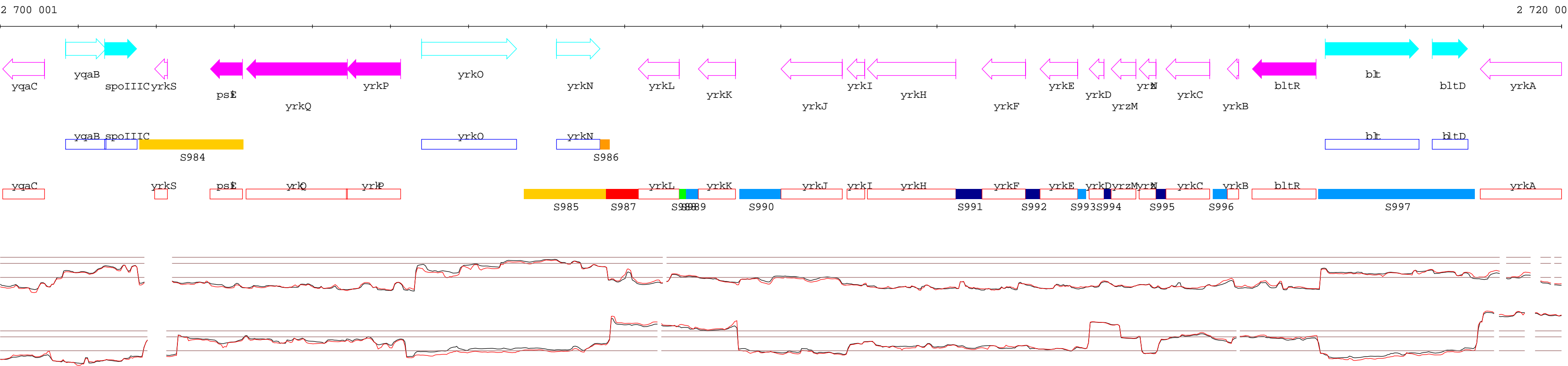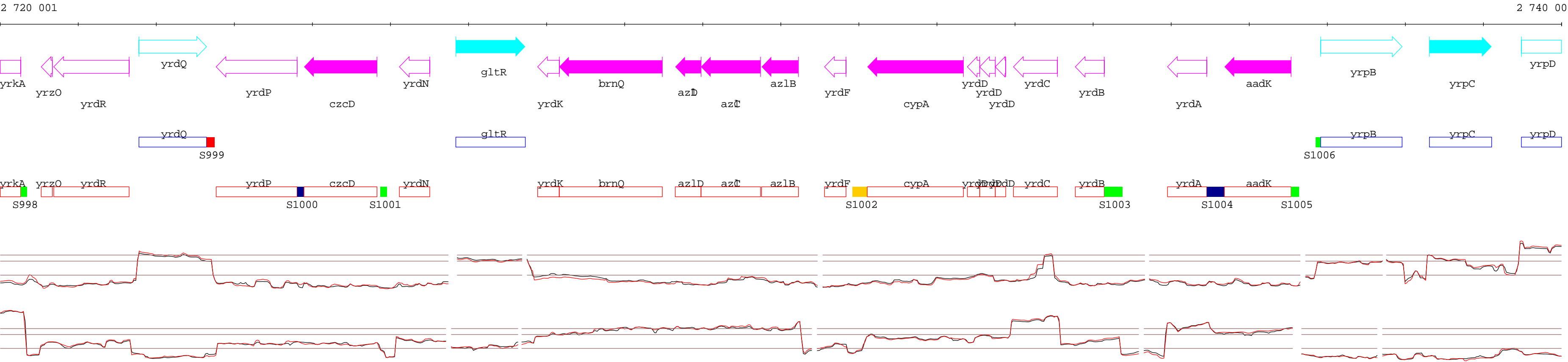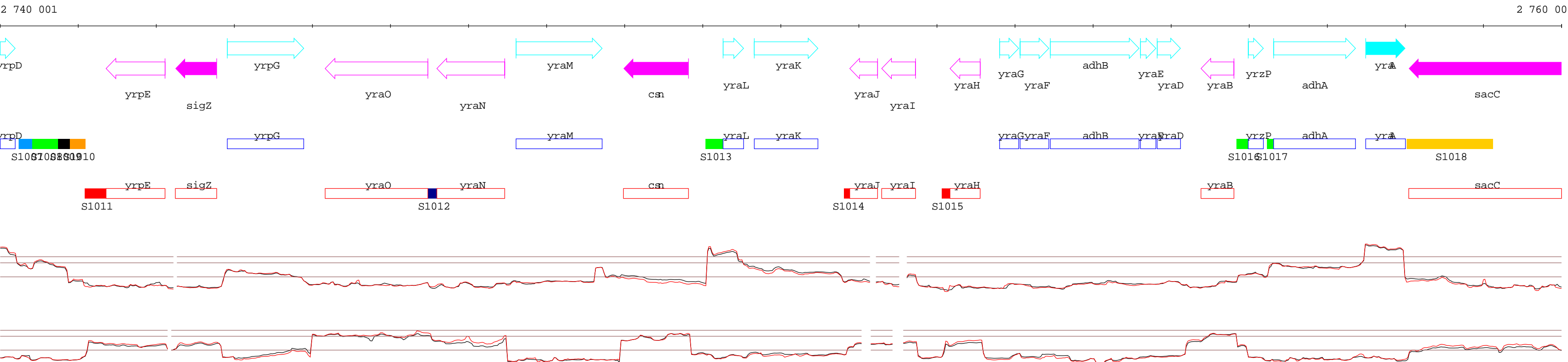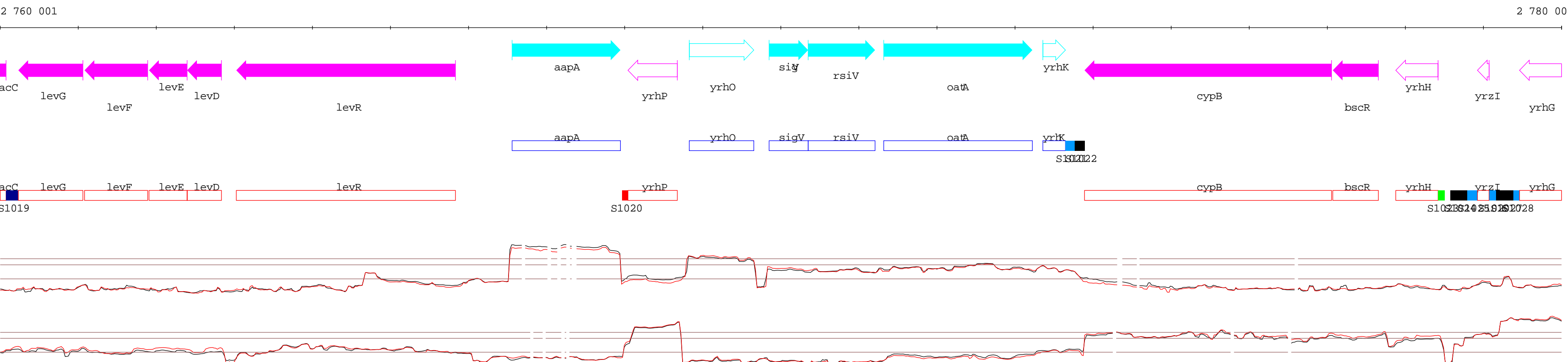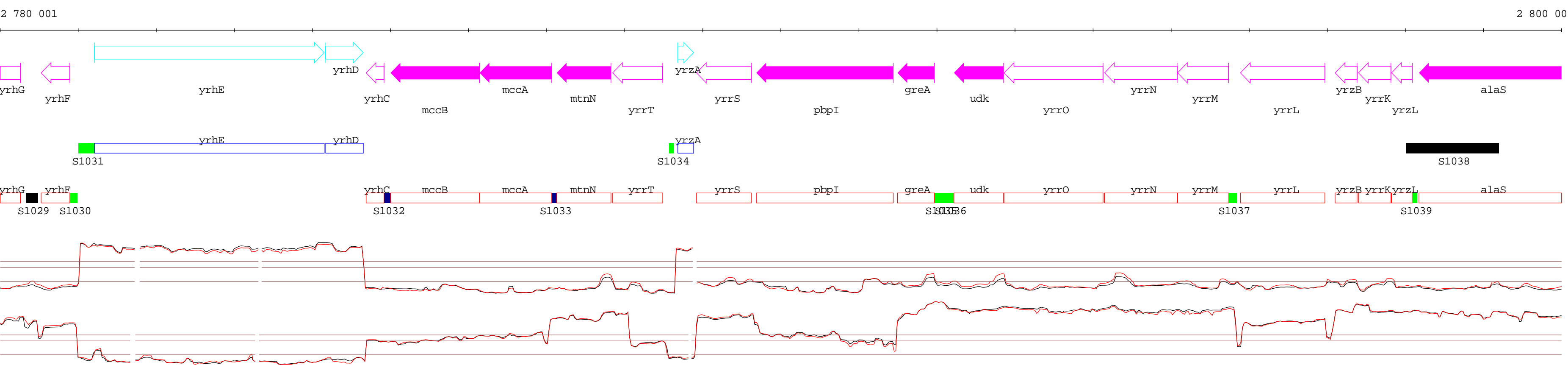

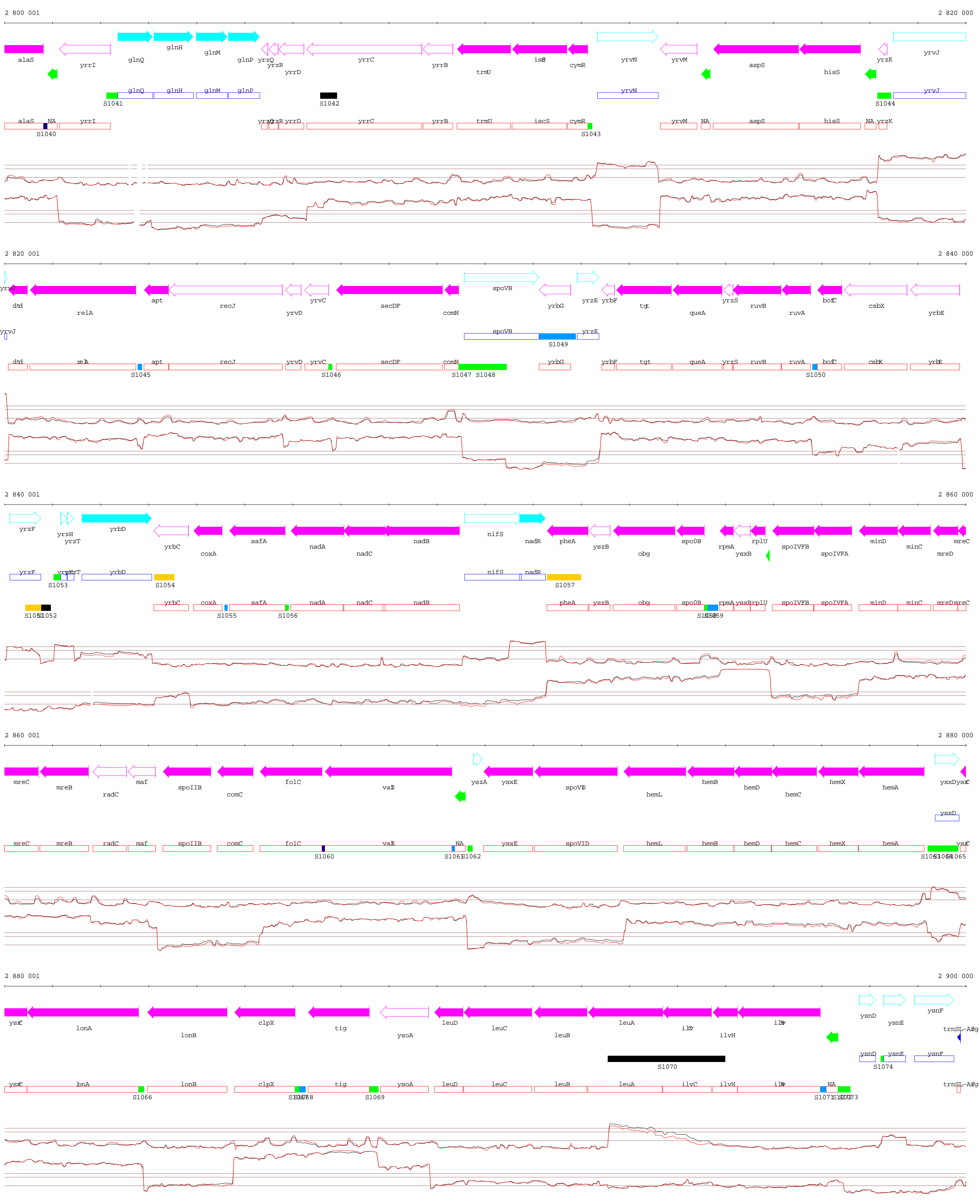

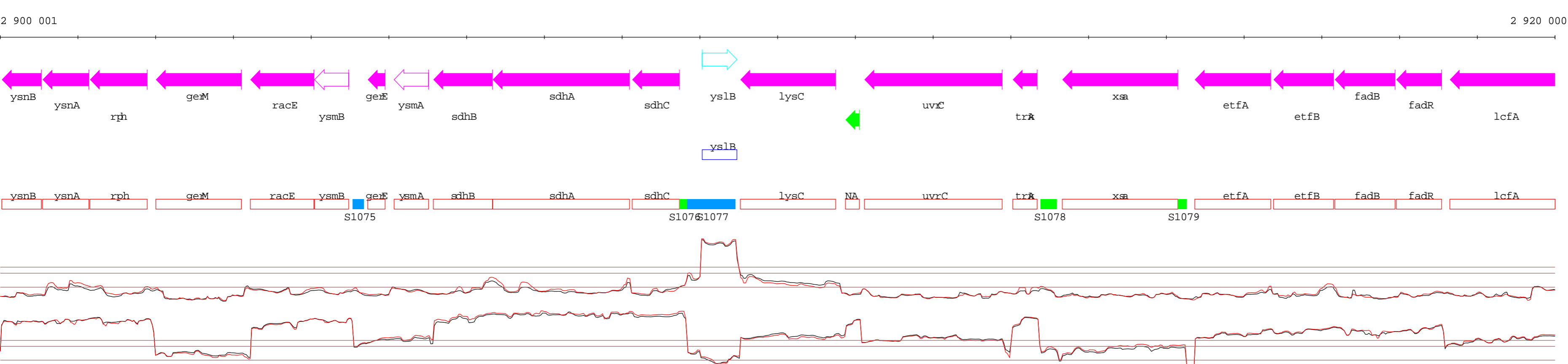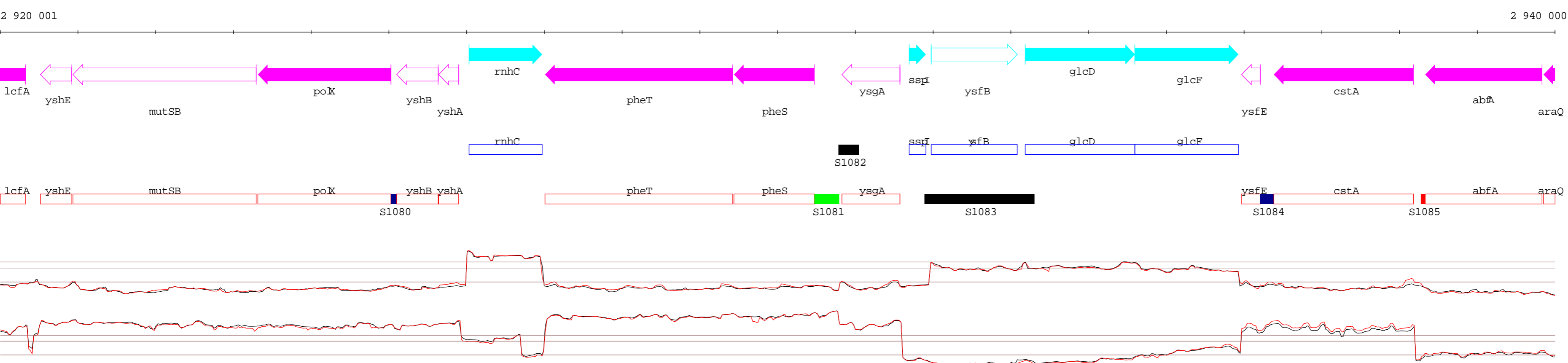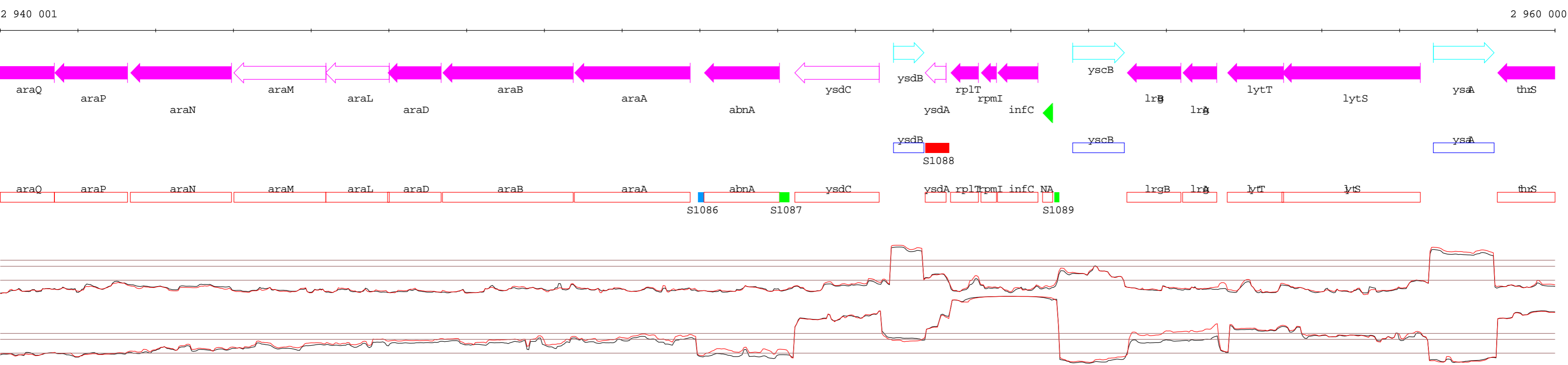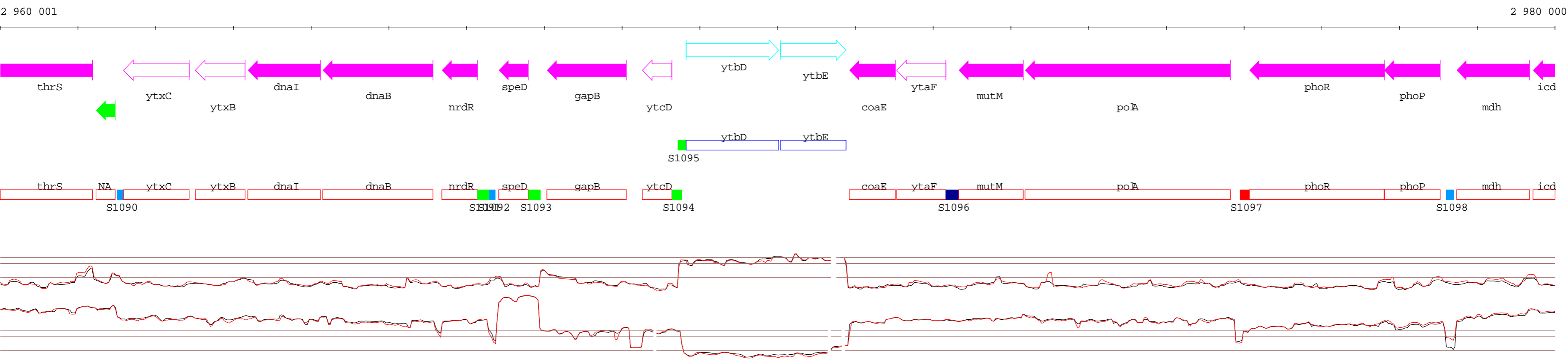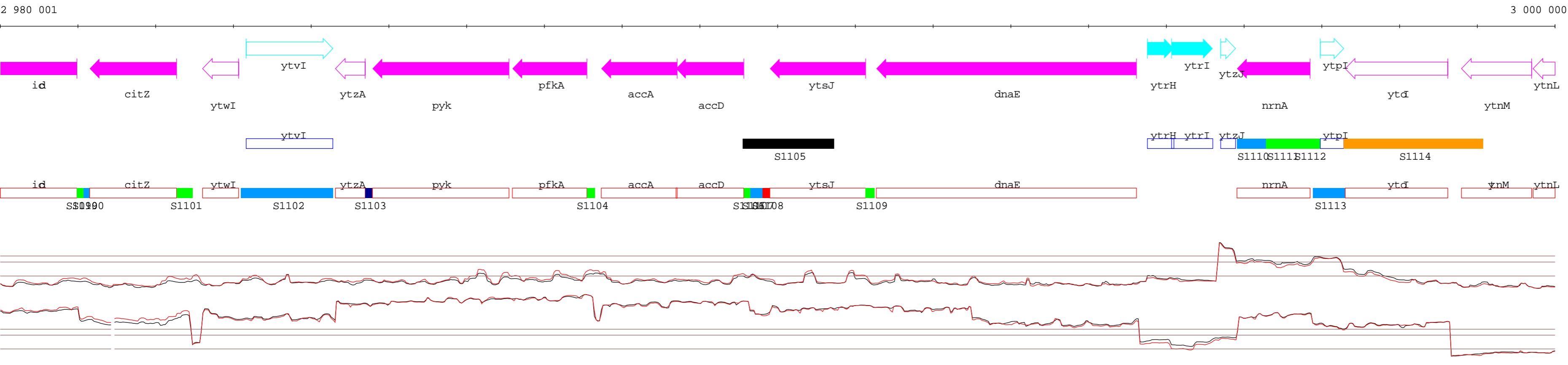

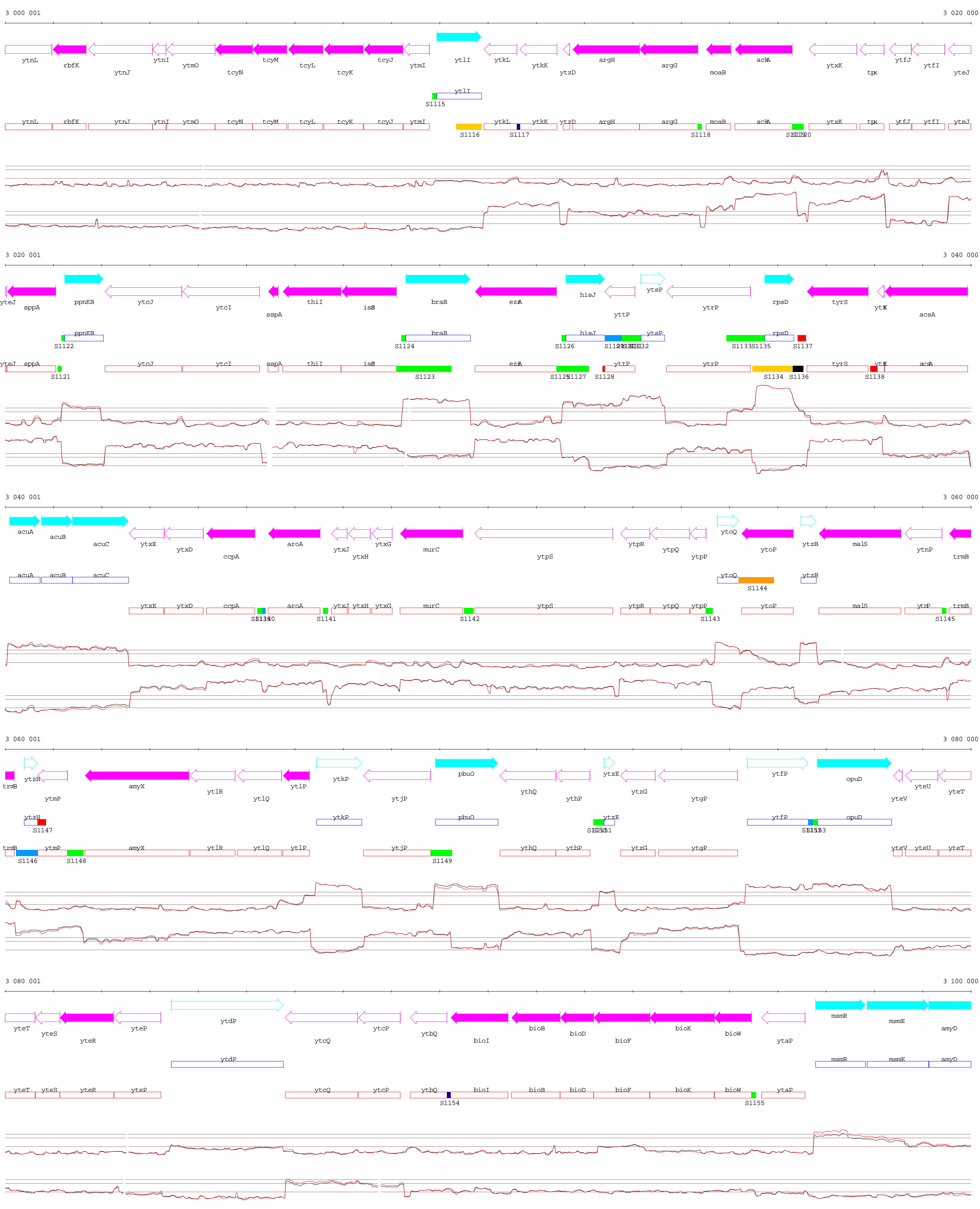

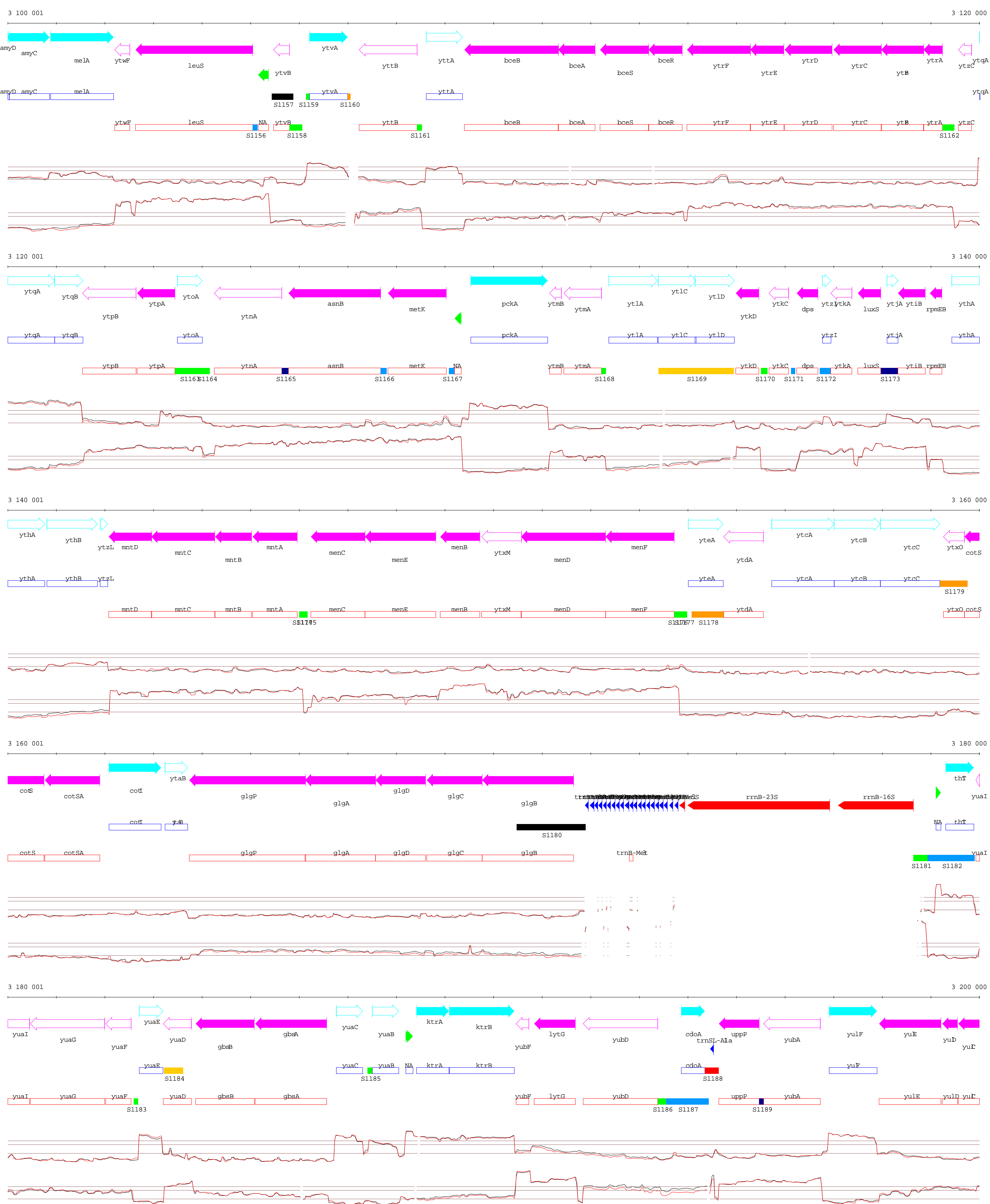

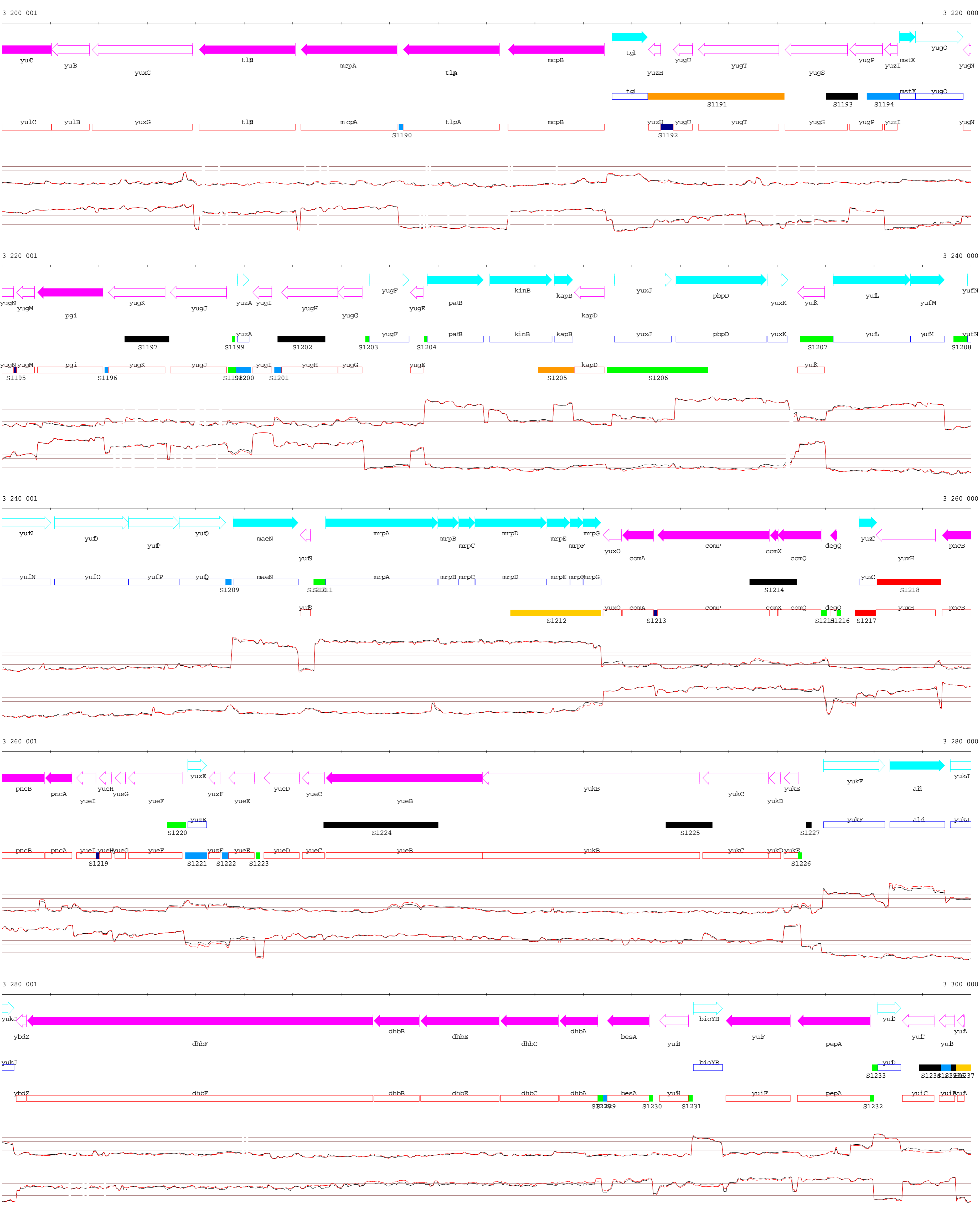

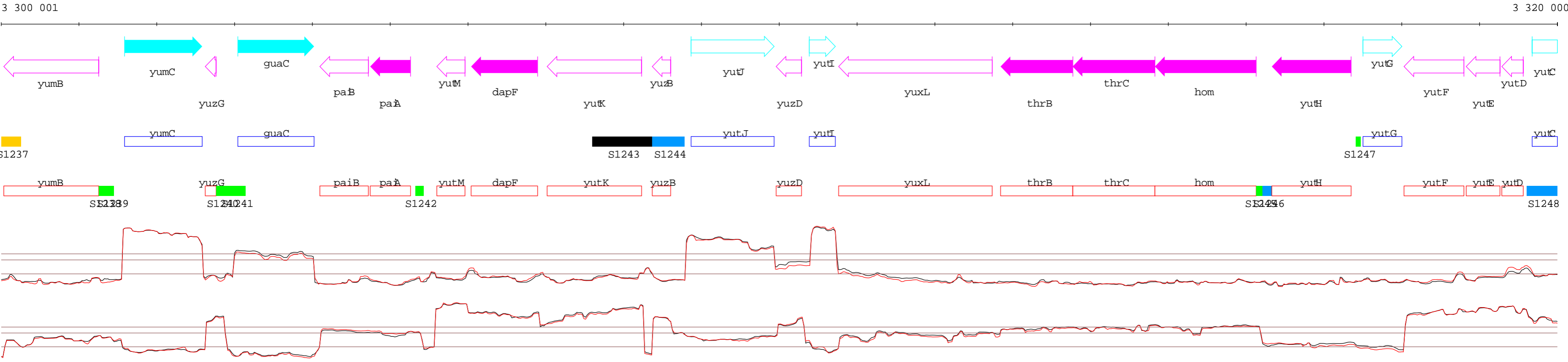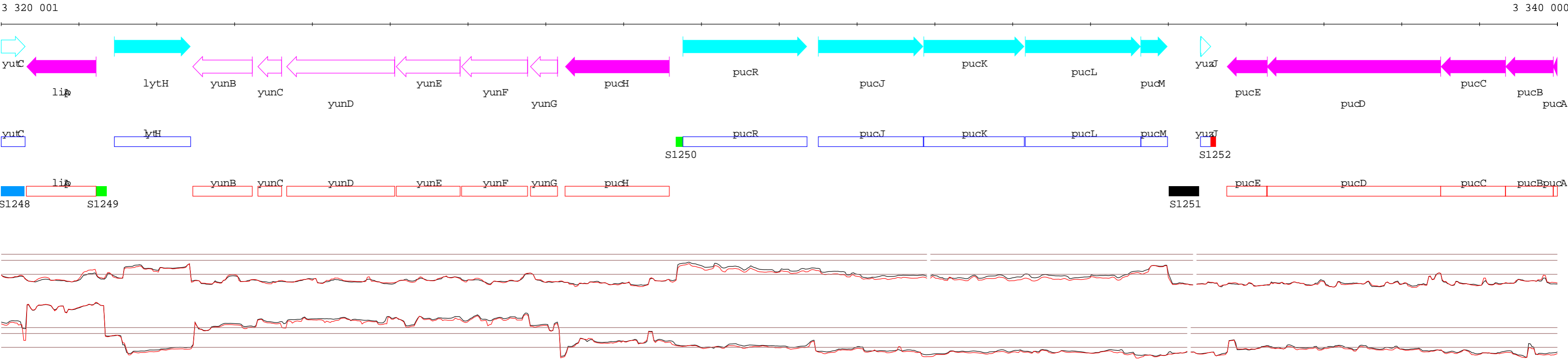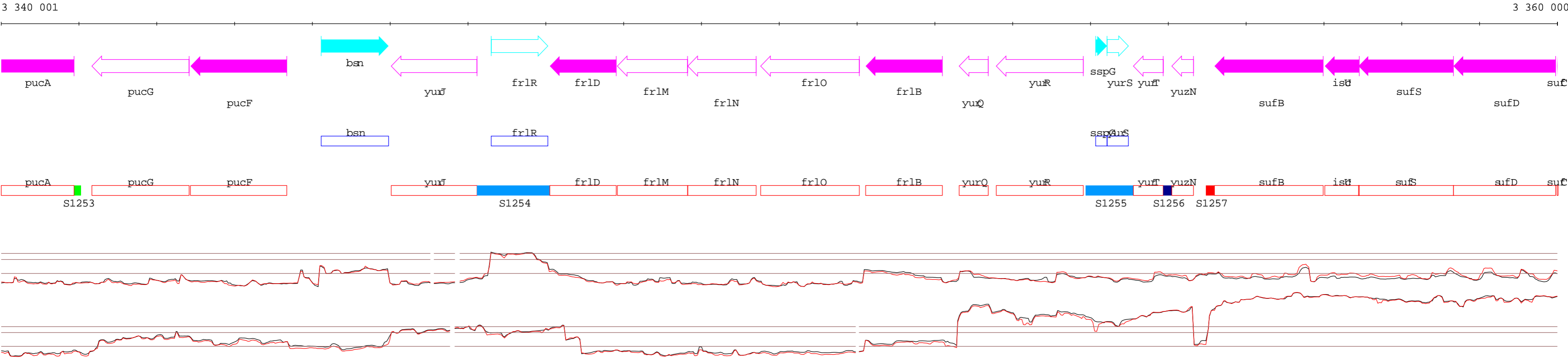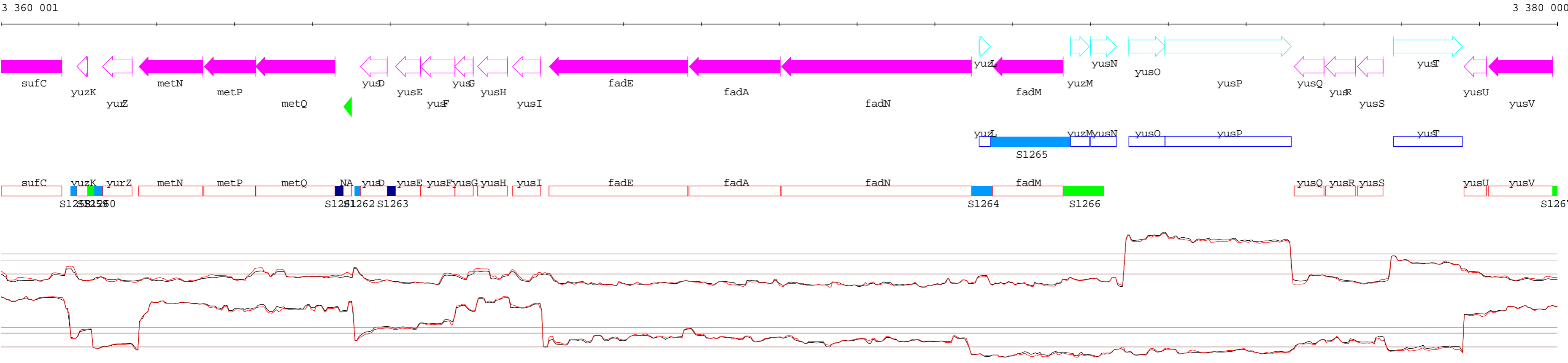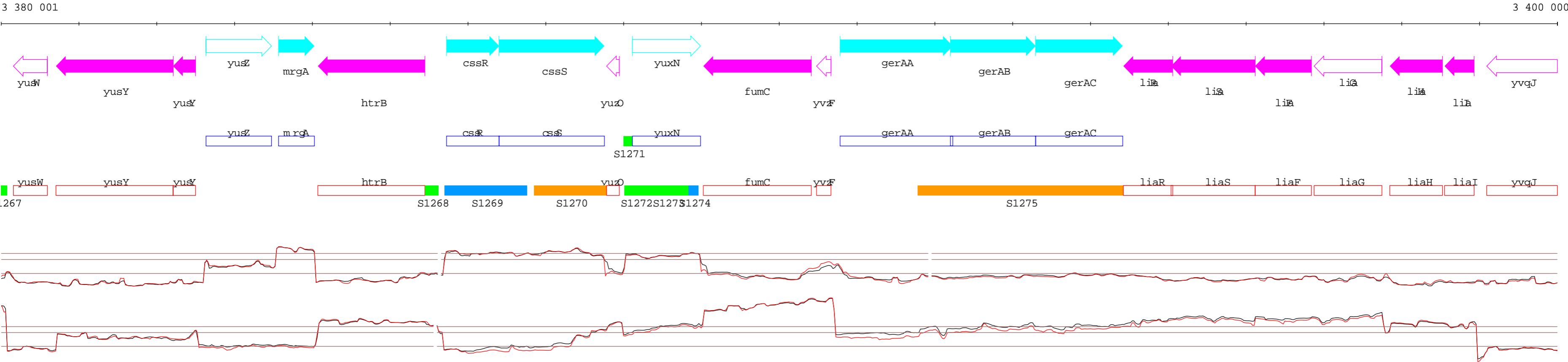

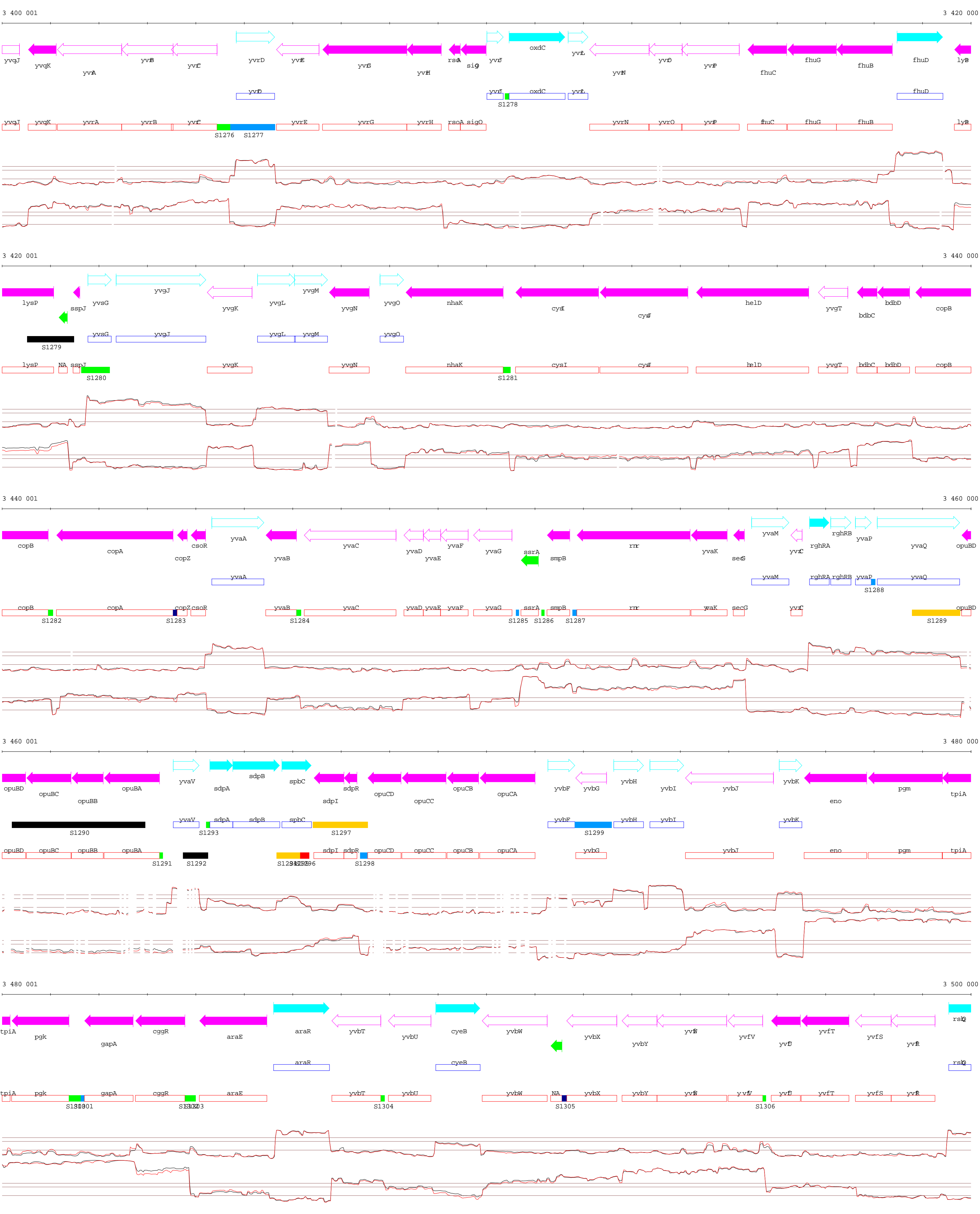

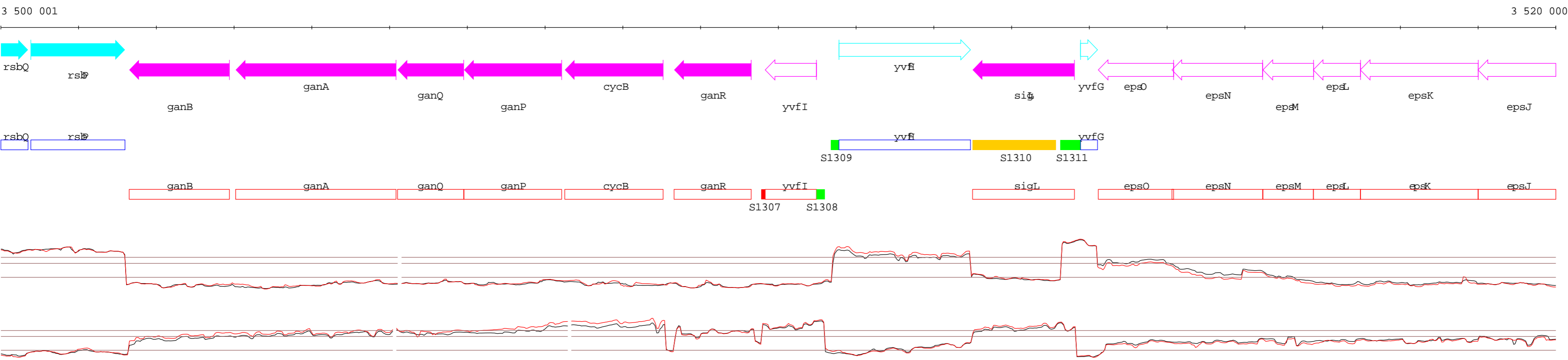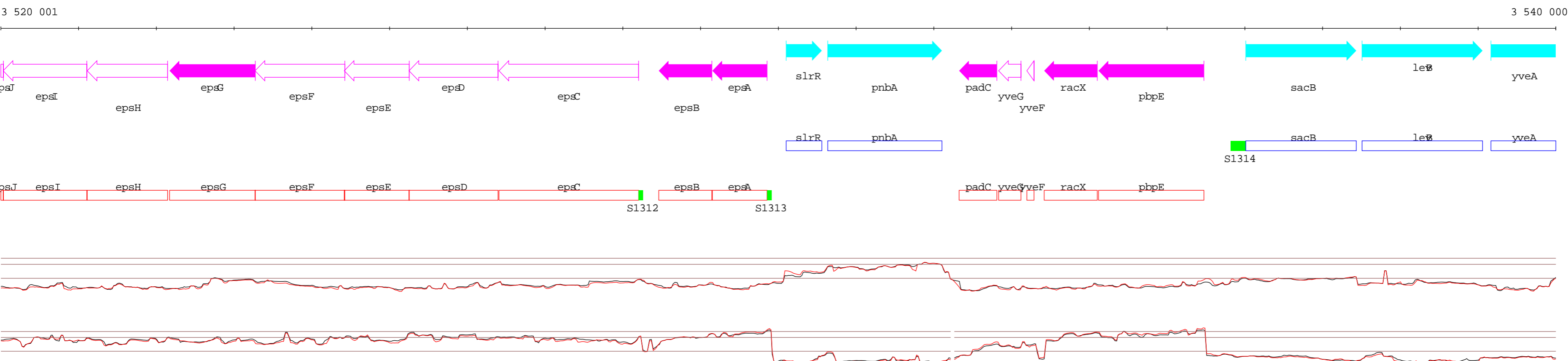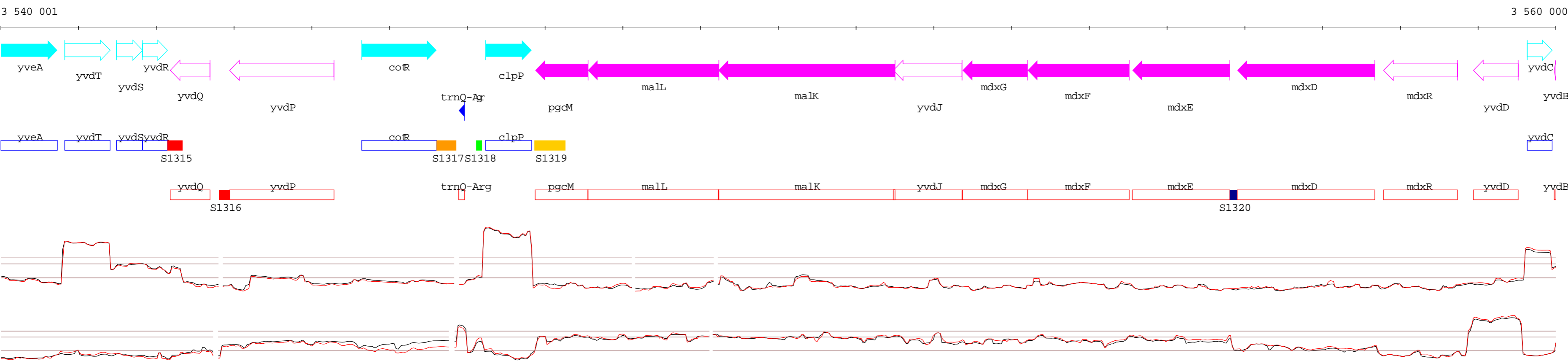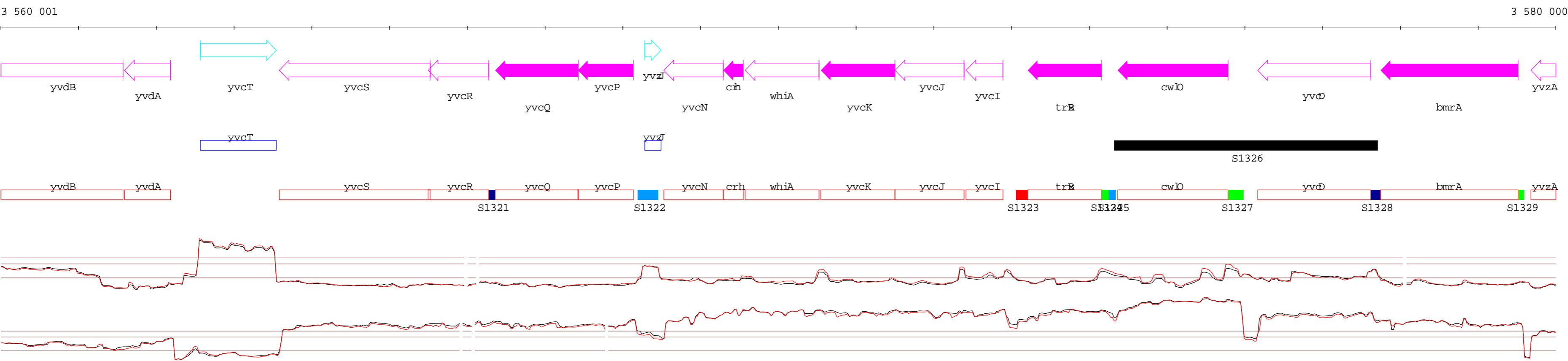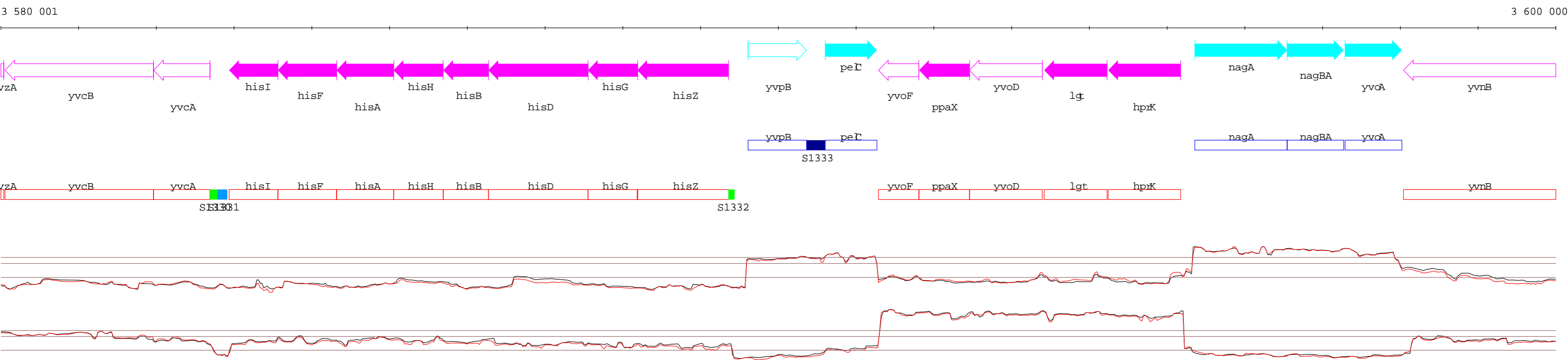

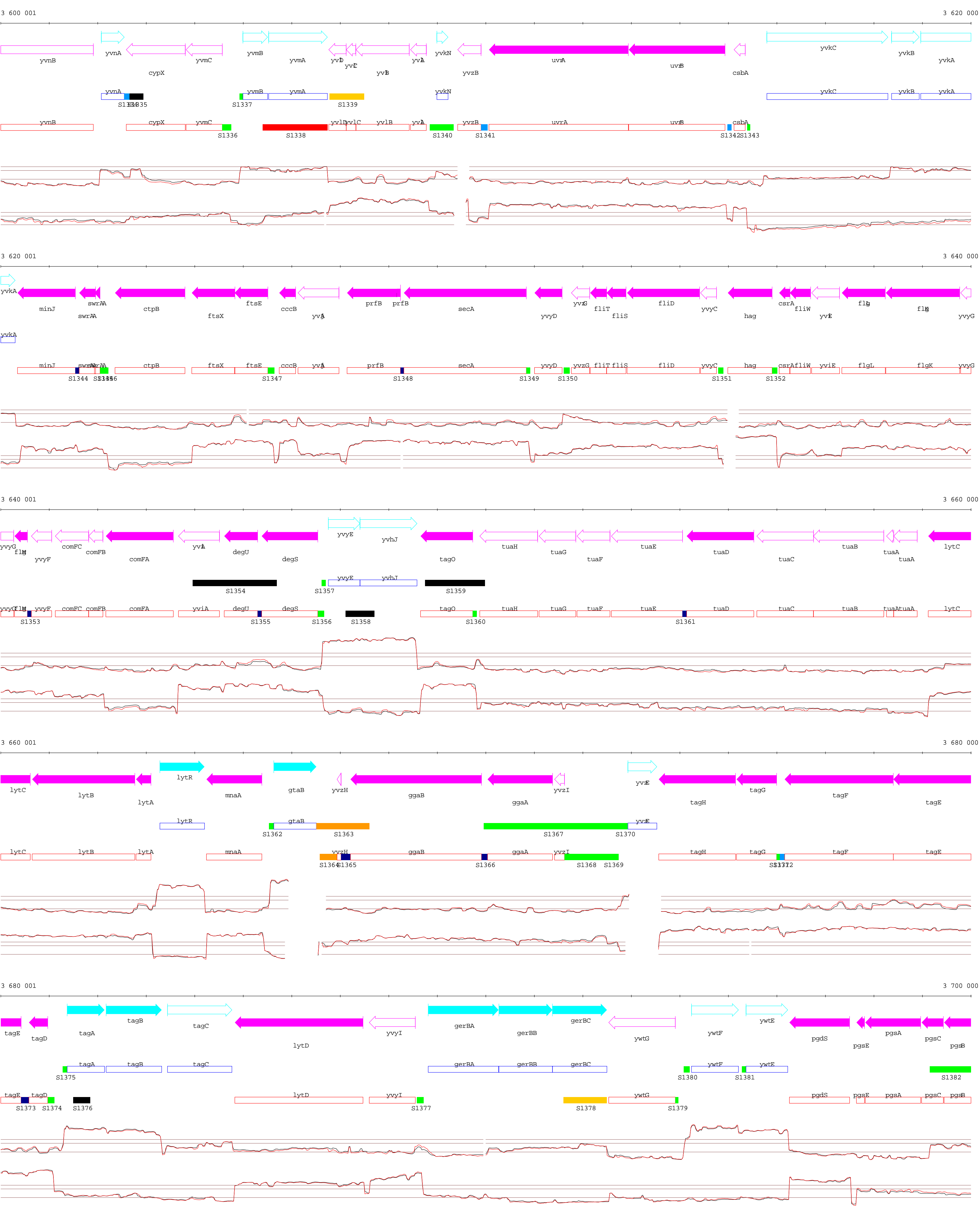

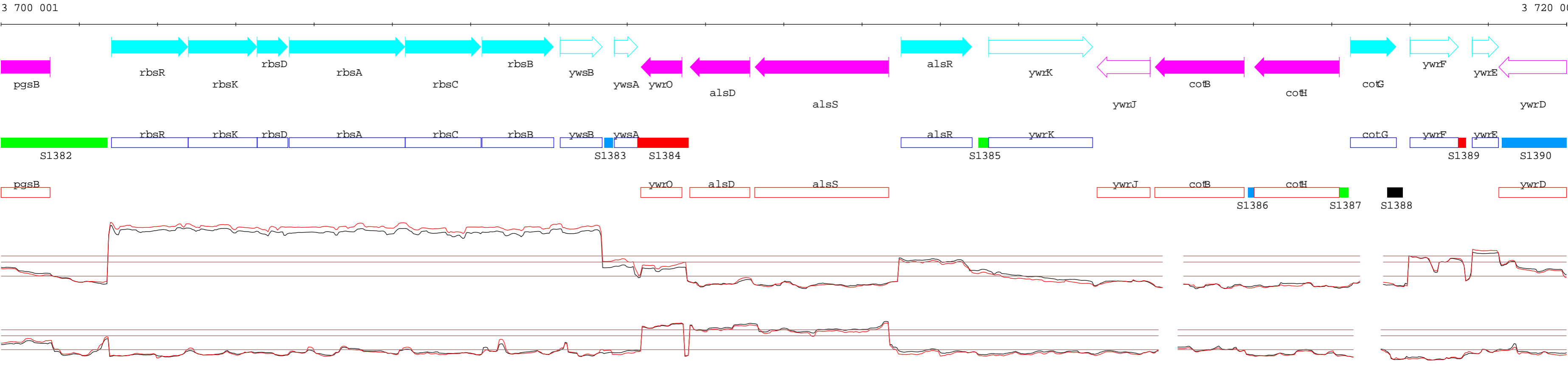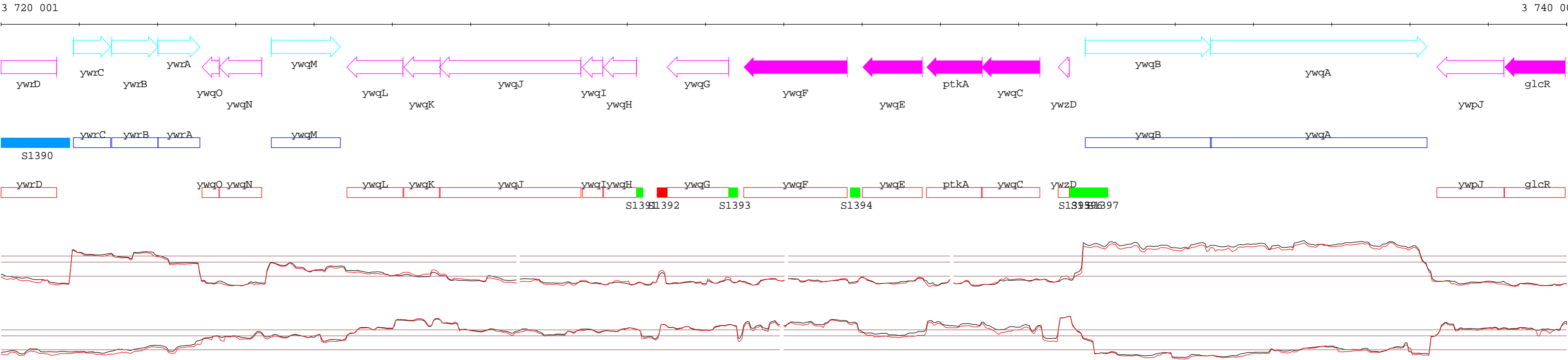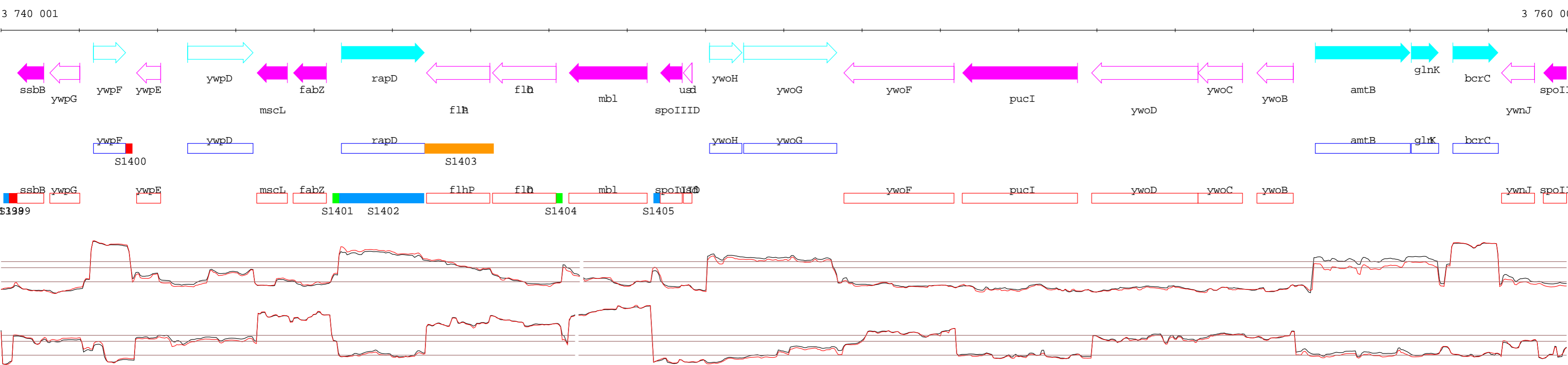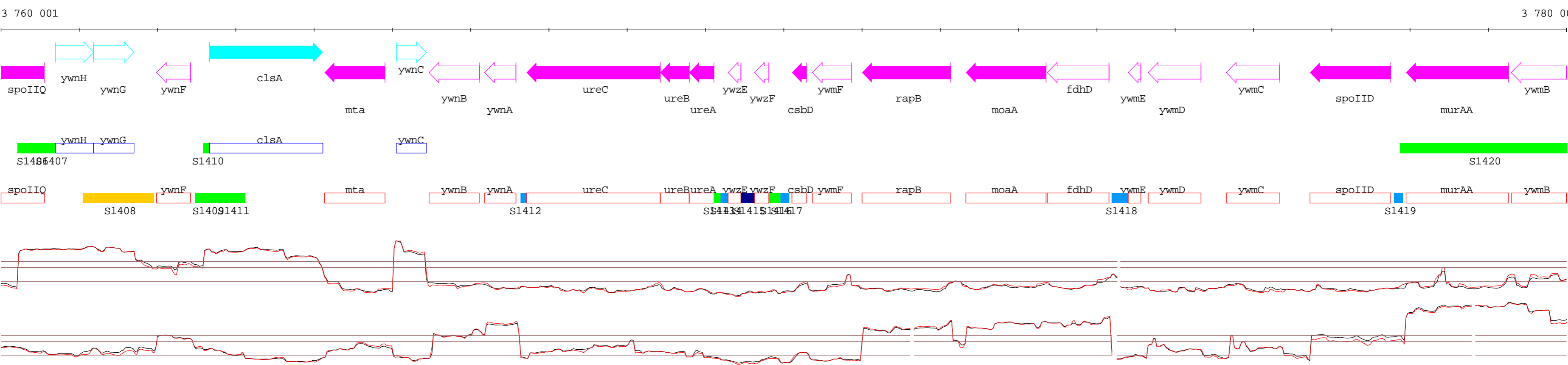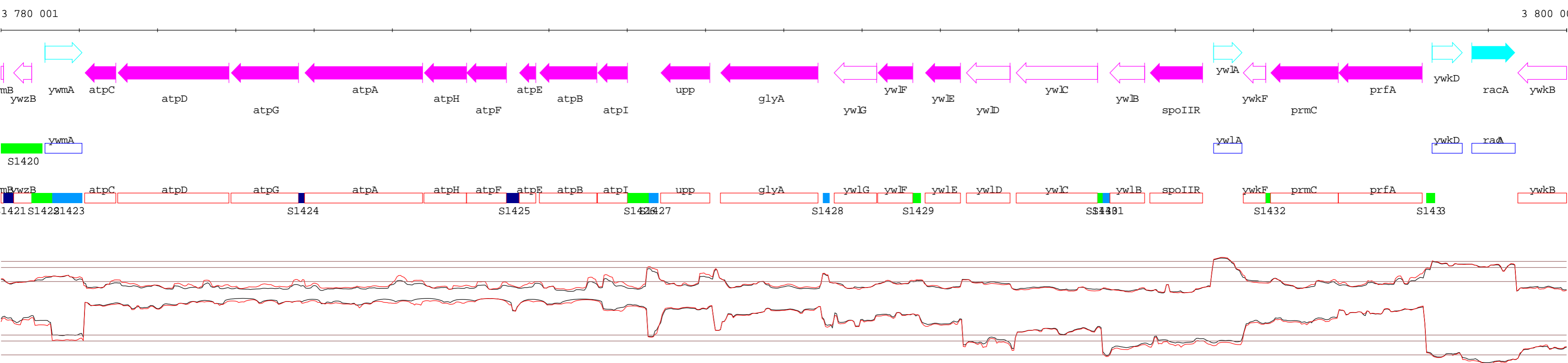

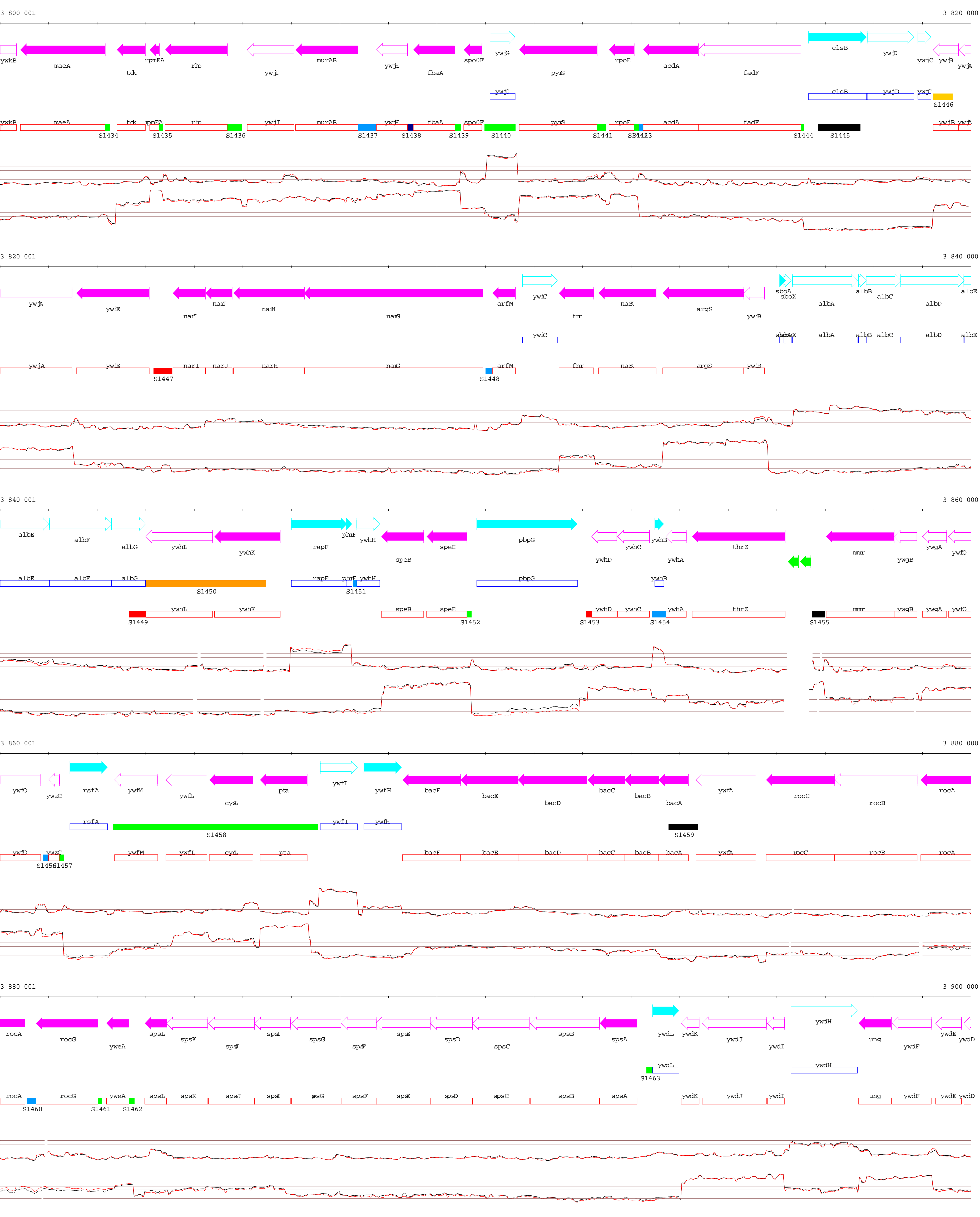

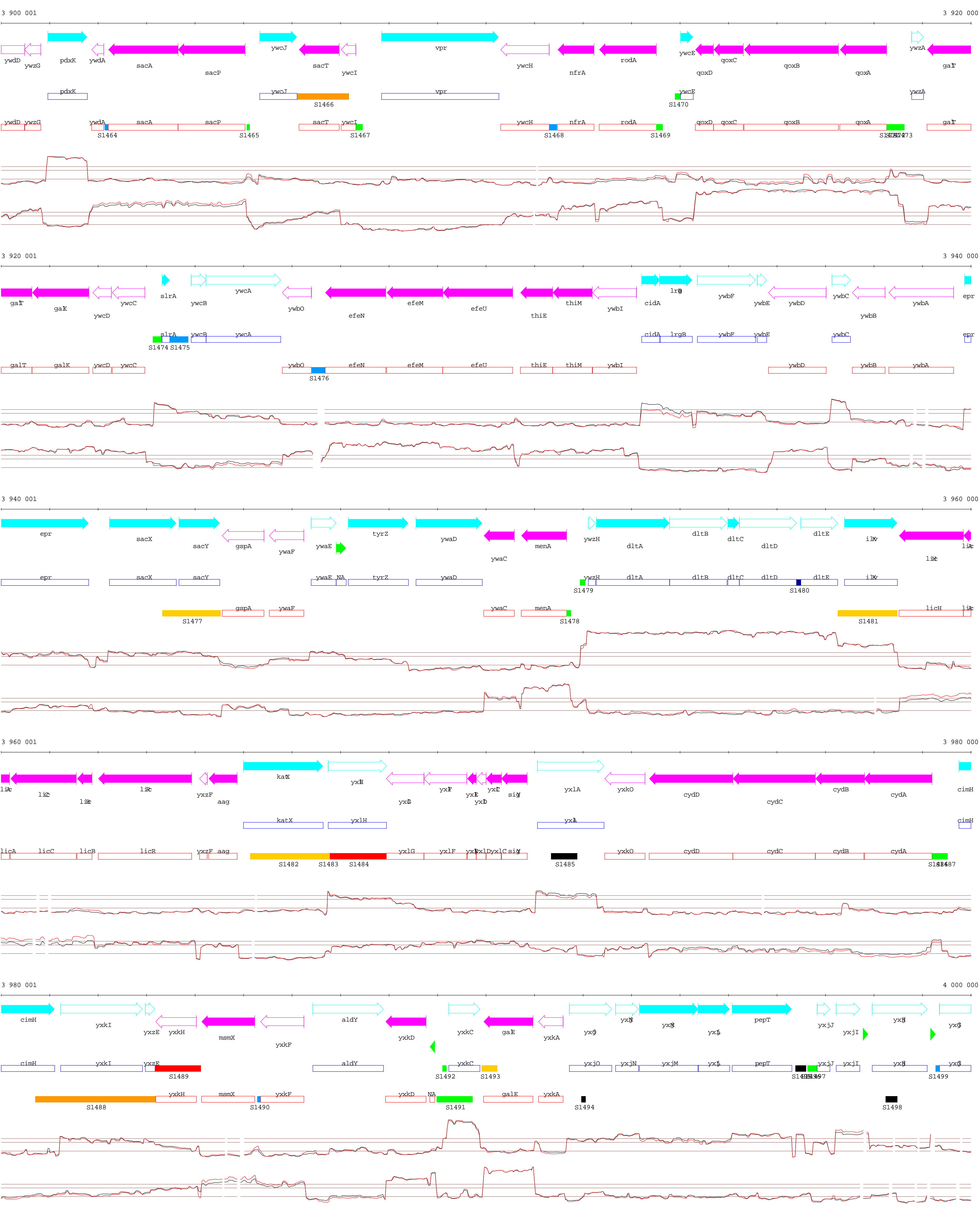

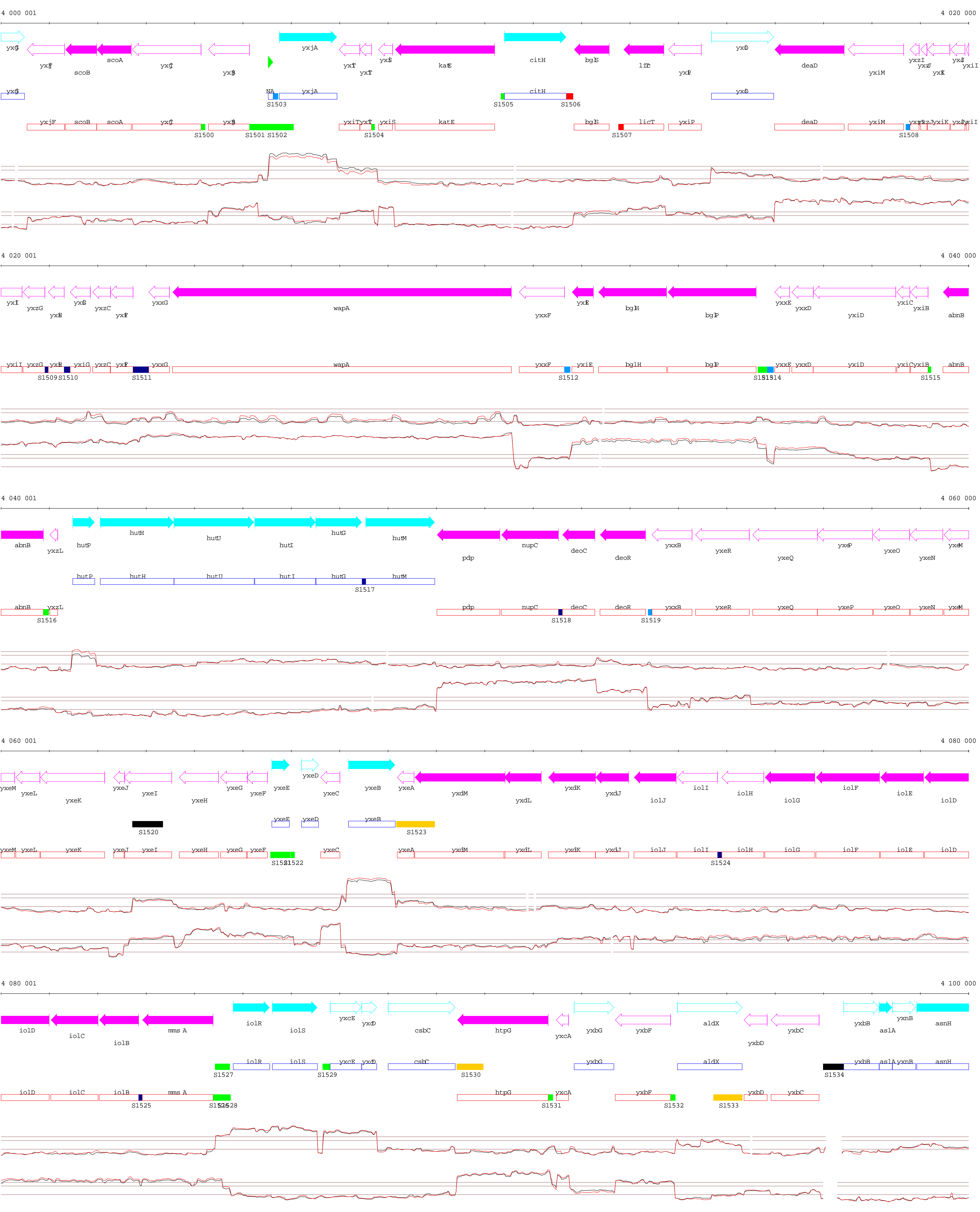

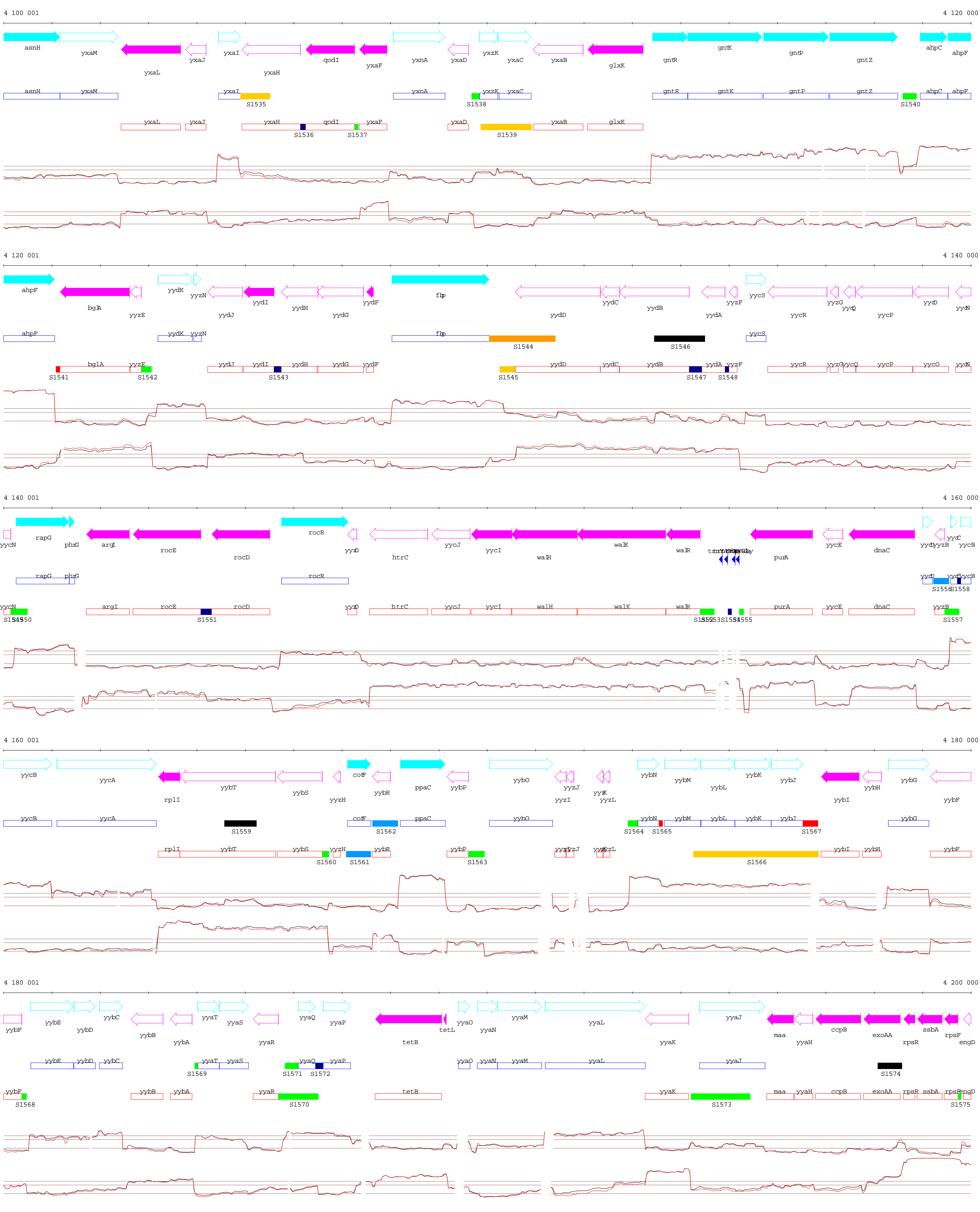

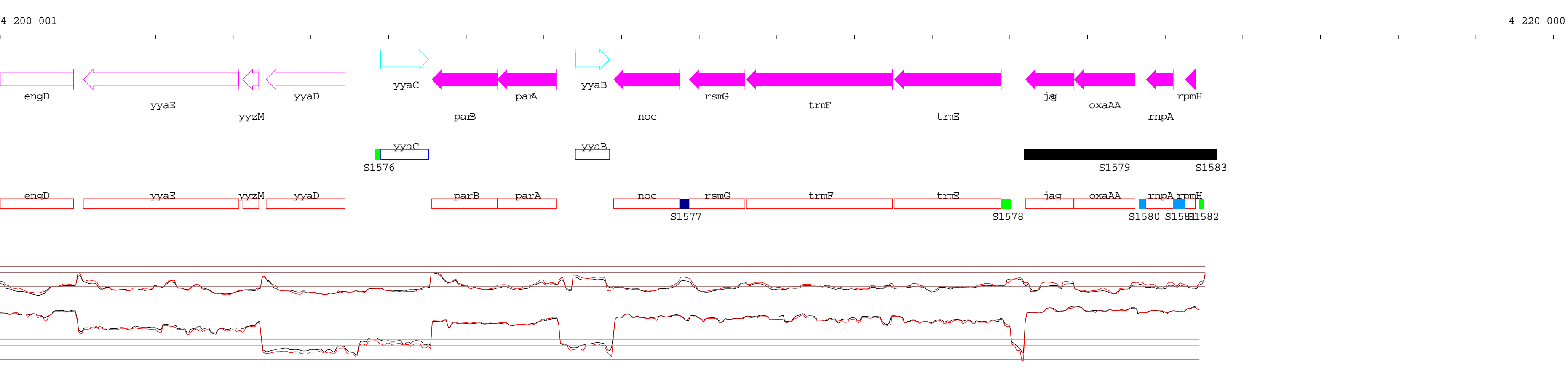

Supplement: S5 File — Genome-wide representation of transcriptome profiles of wild-type and Δhfq strains in exponential phase of growth (Figure legend in S4 File). (PDF) [file pone.0124977.s005.pdf]

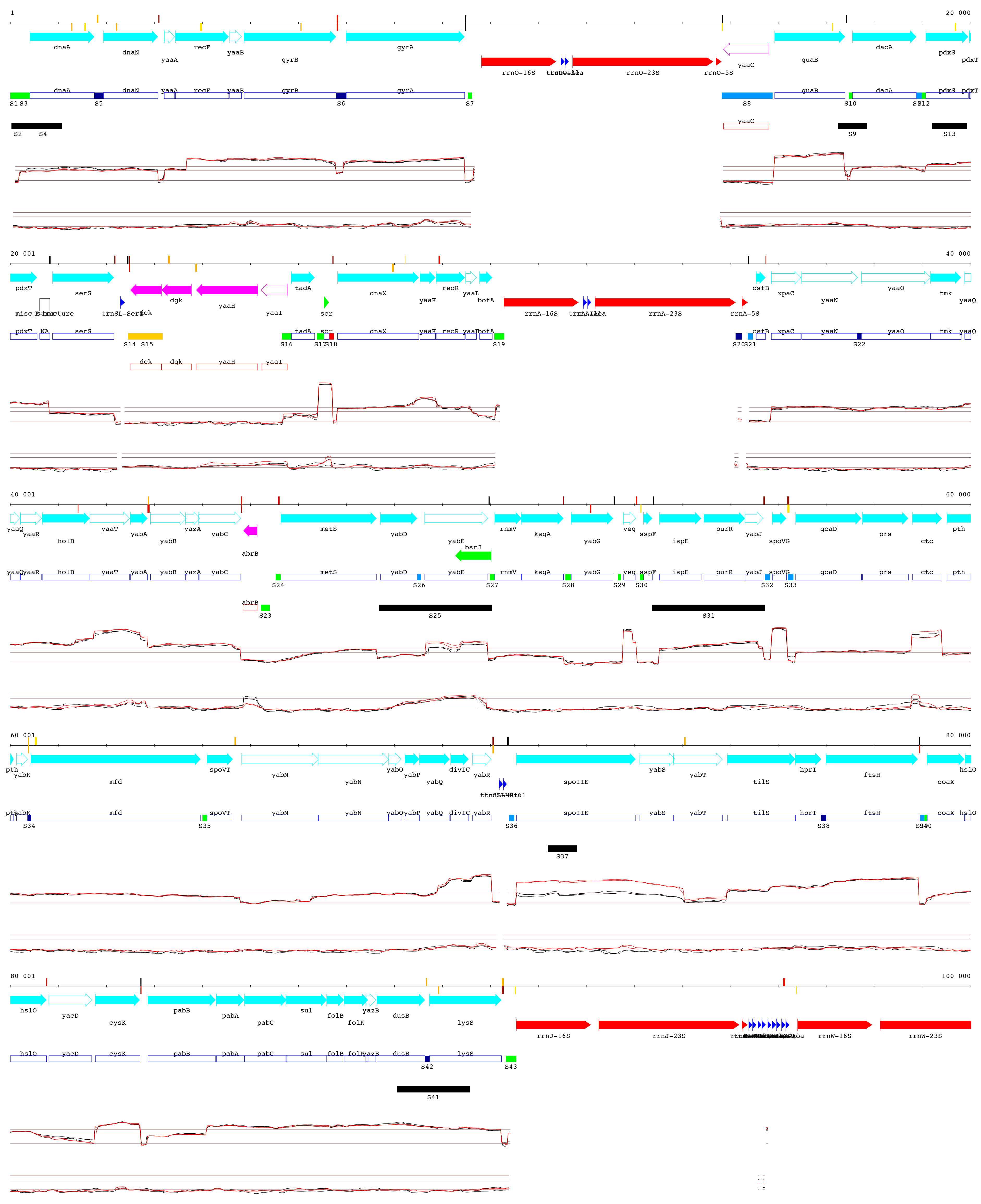

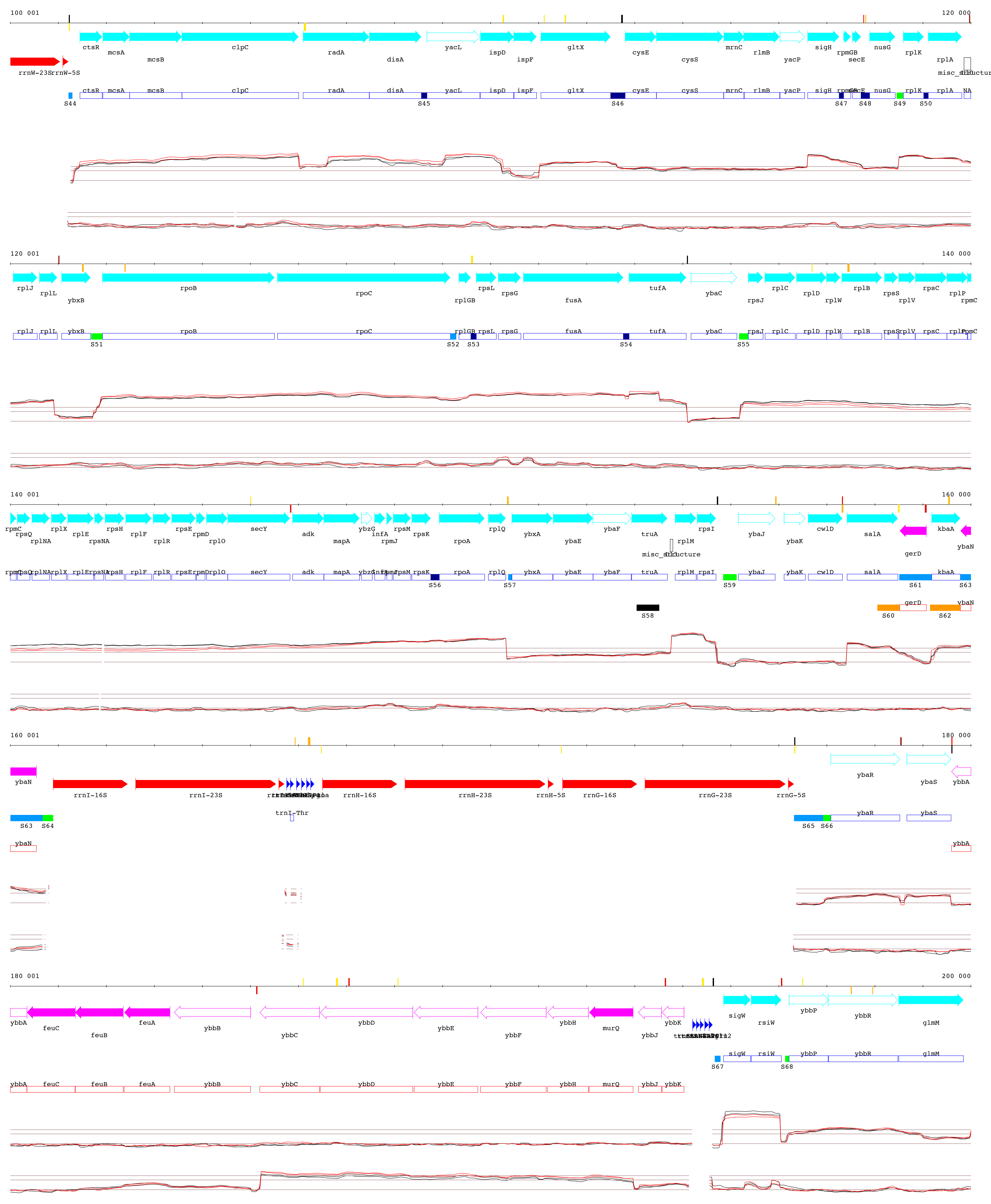

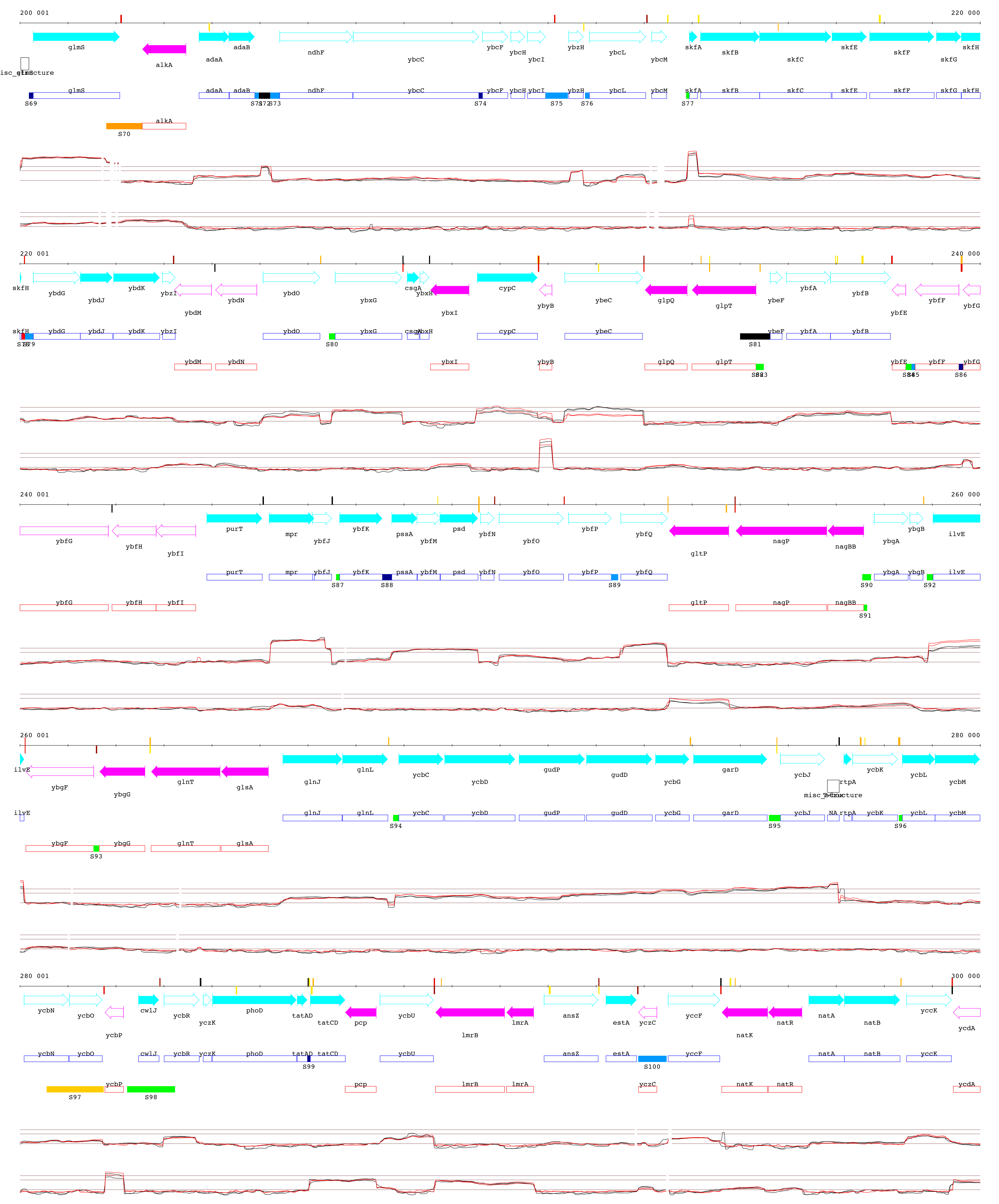

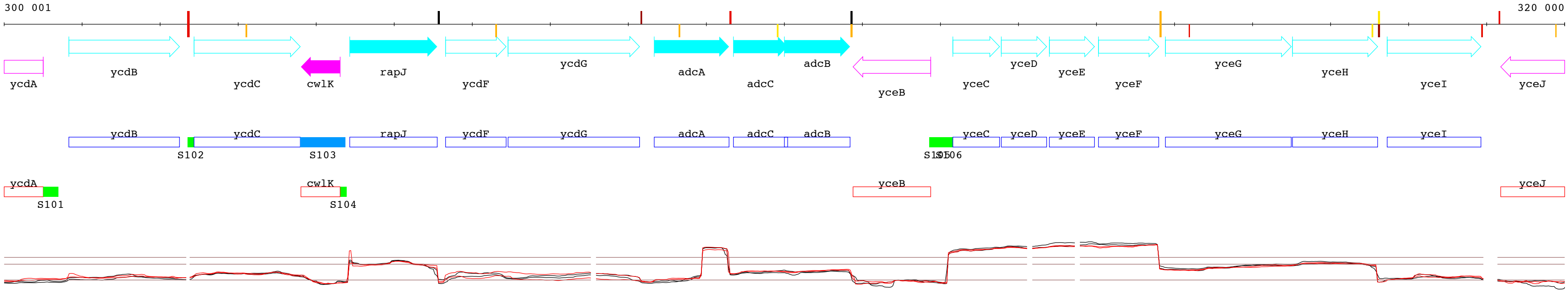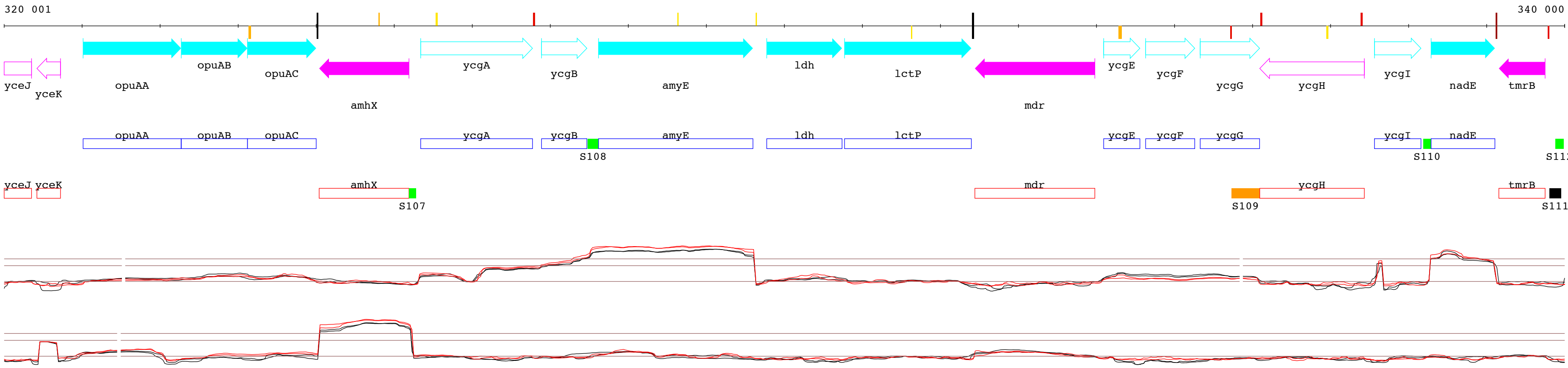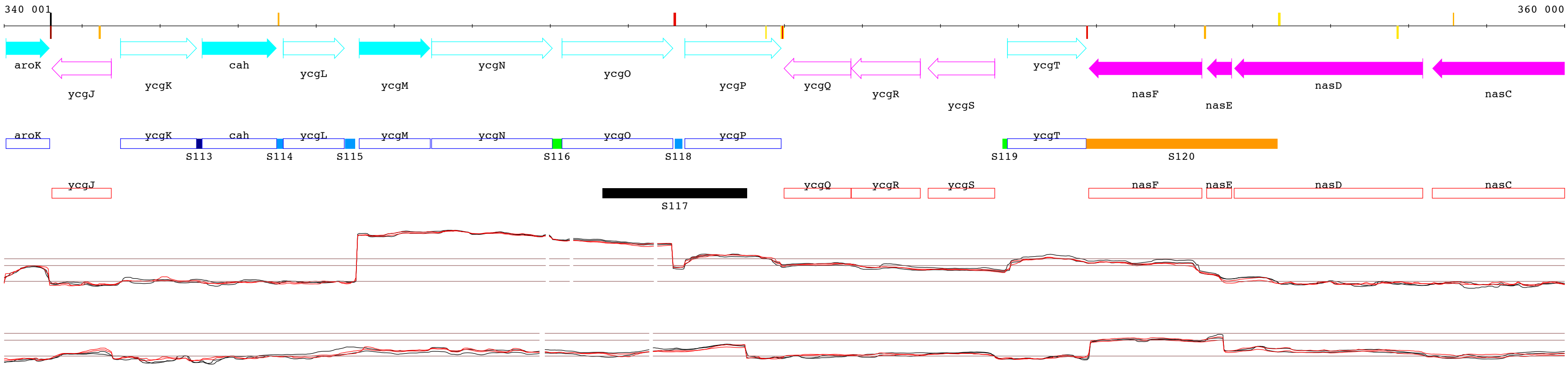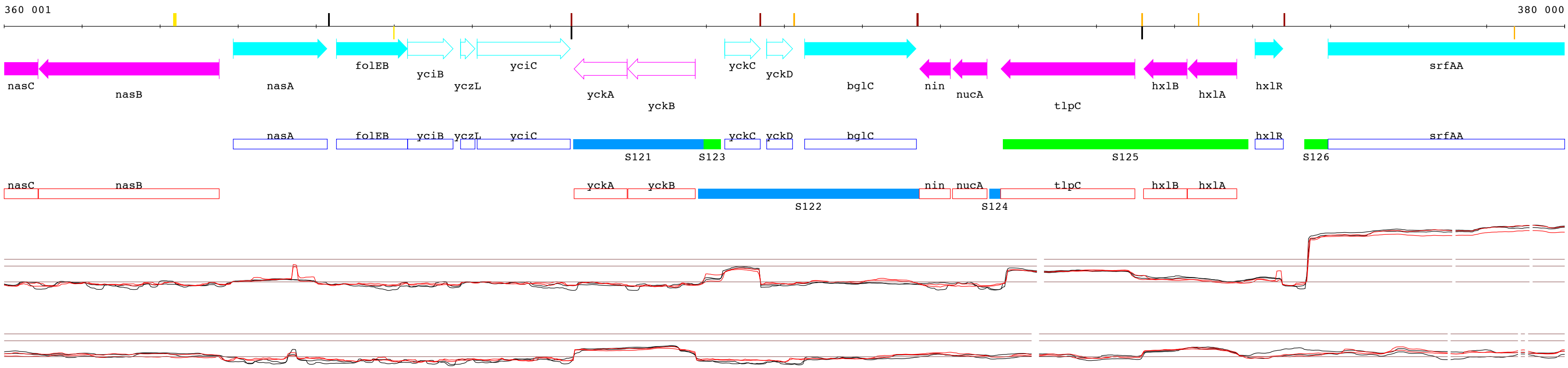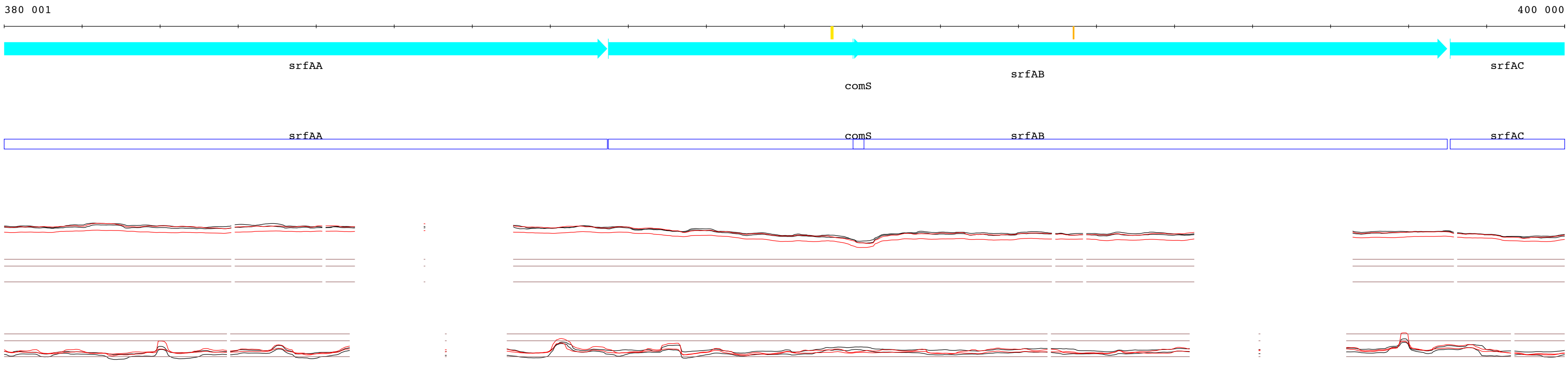



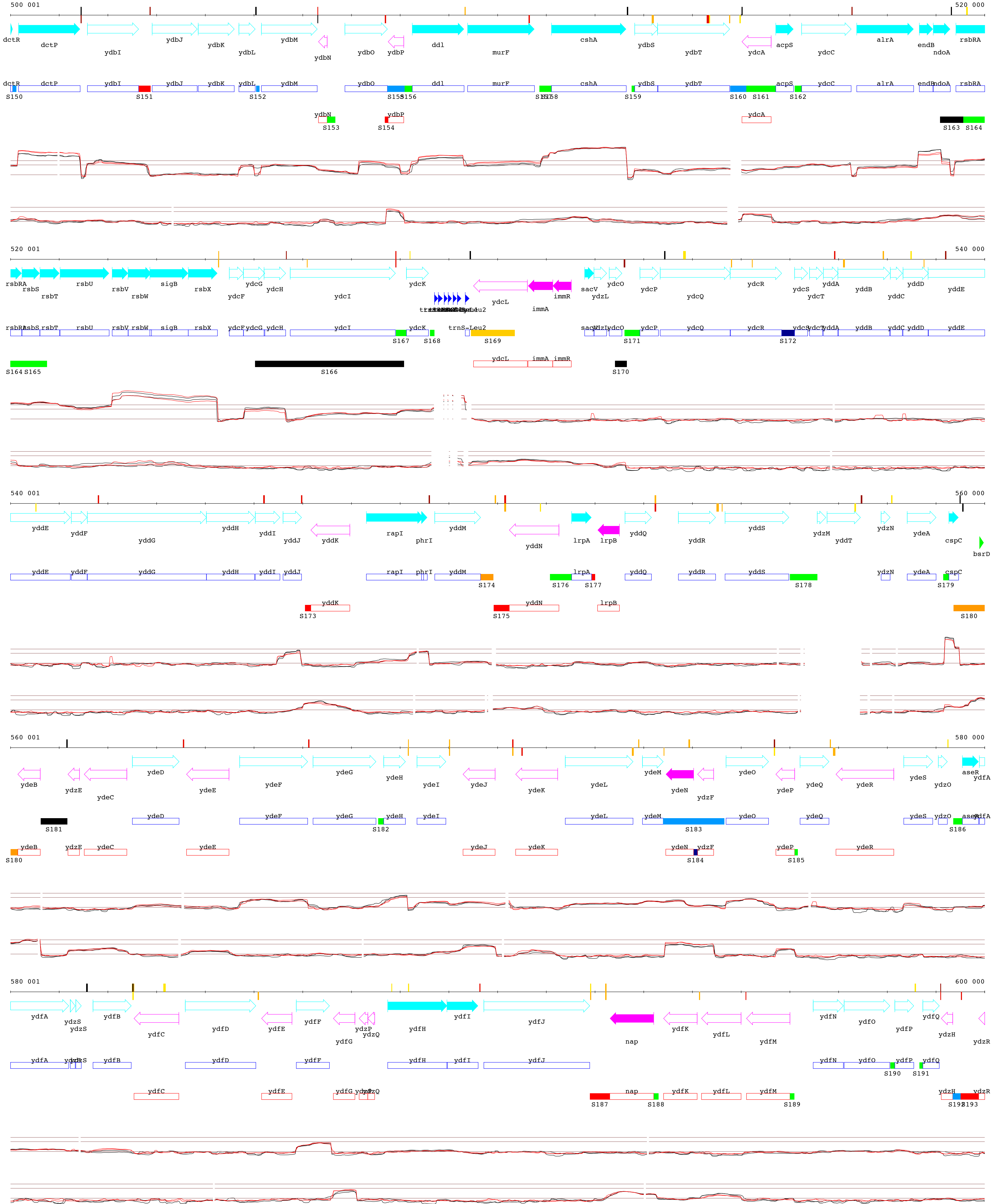





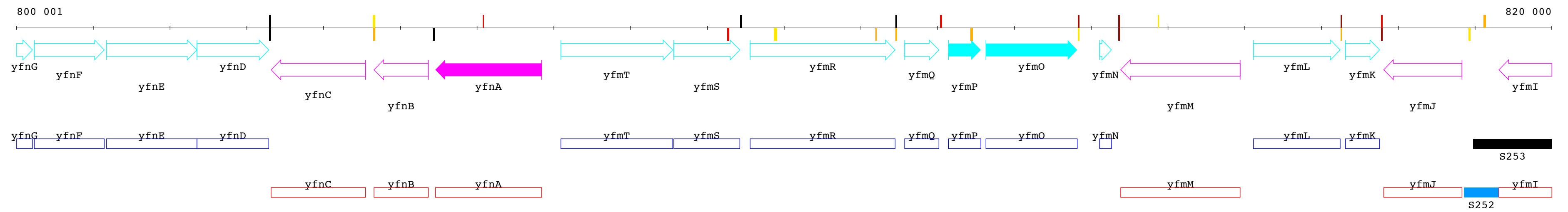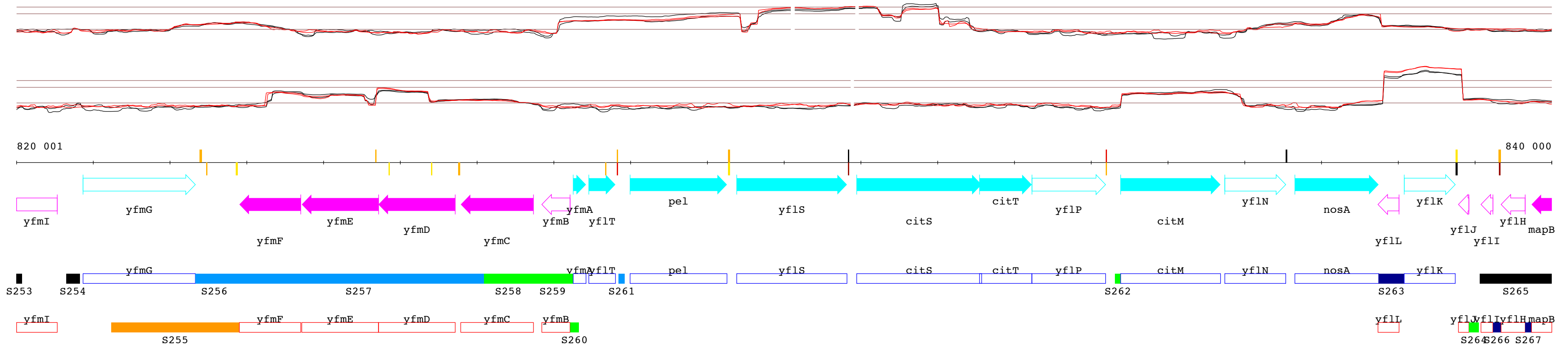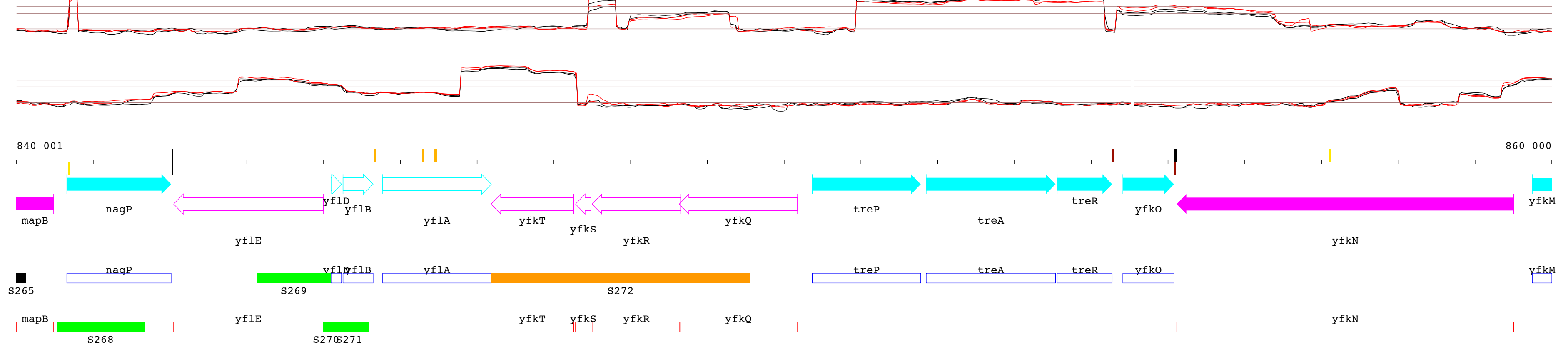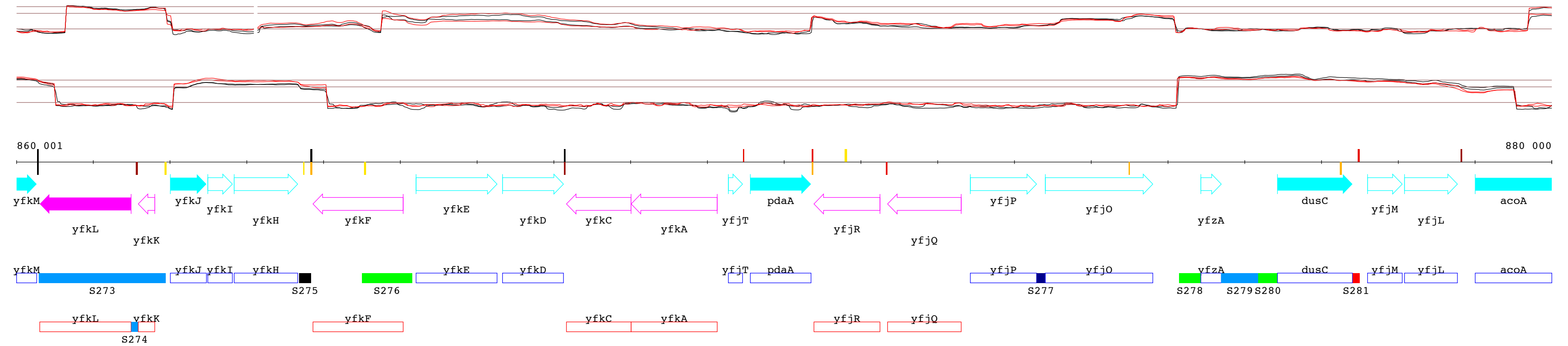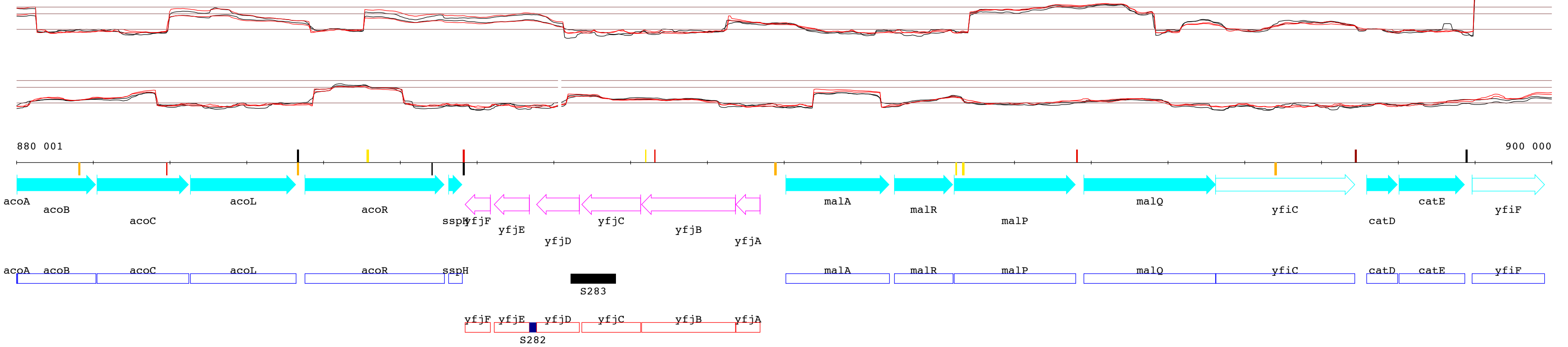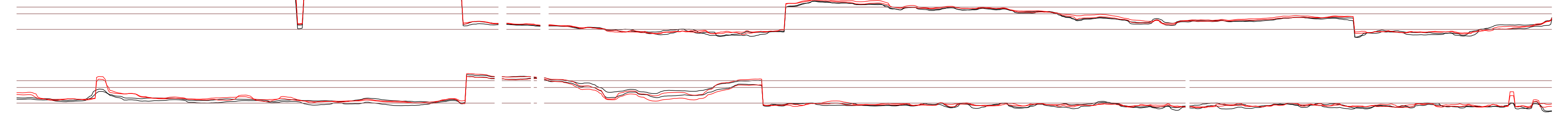

Supplement: S6 File — Genome-wide representation of transcriptome profiles of wild-type and Δhfq strains in stationary phase of growth (Figure legend in S4 File). (PDF) [file pone.0124977.s006.pdf]
