# Supplementary material for: Tracking the Elusive Function of Bacillus subtilis Hfq
Source: PLoS One. 2015 Apr 27;10(4):e0124977. doi: 10.1371/journal.pone.0124977 (PMC4410918; doi:10.1371/journal.pone.0124977)
Supplement: S7 File — (PDF) [file pone.0124977.s007.pdf]

BSB1 Δ*hfq*$_{Bs}$ survival in competition with *hfq*-expressing cells

in the absence of *yebD* or *ctaD*

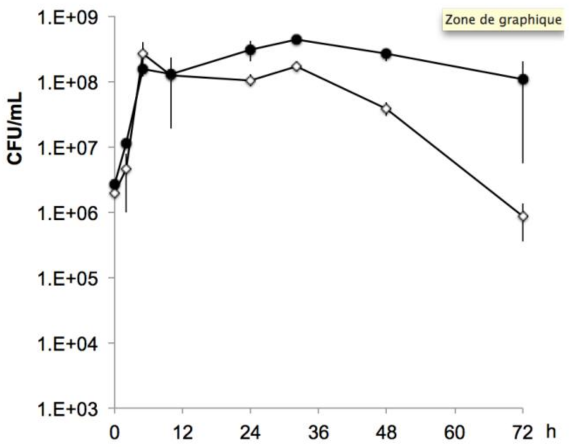

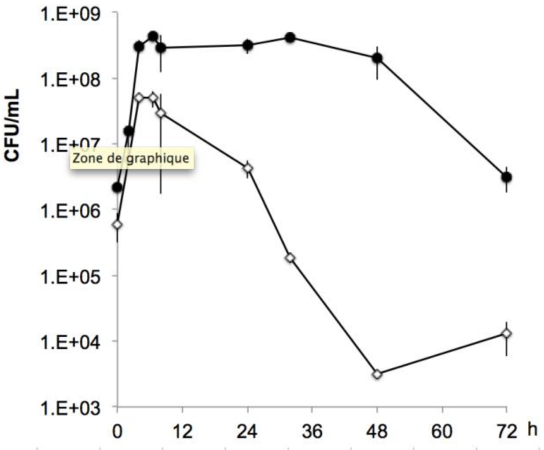

Co-cultures were performed with *hfq*$_{Bs}$-expressing strain and Δ*hfq*$_{Bs}$ mutant in LB medium and incubated at 37°C during 3 days. An antibiotic resistance gene (*spc* or *cat*) was inserted in the intergenic region *hfq-ymzE* (control, black) or in replacement of the *hfq*$_{Bs}$ coding sequence (mutant, white). These two constructions were introduced in two genetic backgrounds: i) a BSB1-derivative strain deleted of *yebD* (upper panel), or ii) a TF8A-derivative strain deleted from a large region including *ctaD* (see Table 1; lower panel). Each population was numbered on LB plates supplemented with spectinomycin or chloramphenicol. These experiments revealed that the decreased fitness observed in absence of *hfq*$_{Bs}$ remains in these two genetic backgrounds. We conclude that Hfq acts independently of the presence of *yebD* or *ctaD* (as well as of all genes of the deleted region namely: *pycA*, *ctaA* and *ctaBCDEFG* operon, see Table 1 for detailed genotypes).
