# Supplementary material for: Tracking the Elusive Function of Bacillus subtilis Hfq
Source: PLoS One. 2015 Apr 27;10(4):e0124977. doi: 10.1371/journal.pone.0124977 (PMC4410918; doi:10.1371/journal.pone.0124977)
Supplement: S8 File — (PDF) [file pone.0124977.s008.pdf]

ahrC expression in *hfq*$_{Bs}$-expressing strain and Δ*hfq*$_{Bs}$ mutant

The BSB1 strain was used to express a C-terminally SPA tagged AhrC (AhrC^{SPA}) by constructing a translational fusion of the *ahrC* coding sequence and the SPA tag sequence at the locus. In the resulting strain (Bas028) *ahrC-SPA* is expressed under the control of its own promoter. This fusion was transferred in *hfq* deleted strains (TR223 *hfq::cat* or TR232 *hfq::spec*), and the quantity of AhrC was measured by Western-blot in cells grown in LB rich medium.

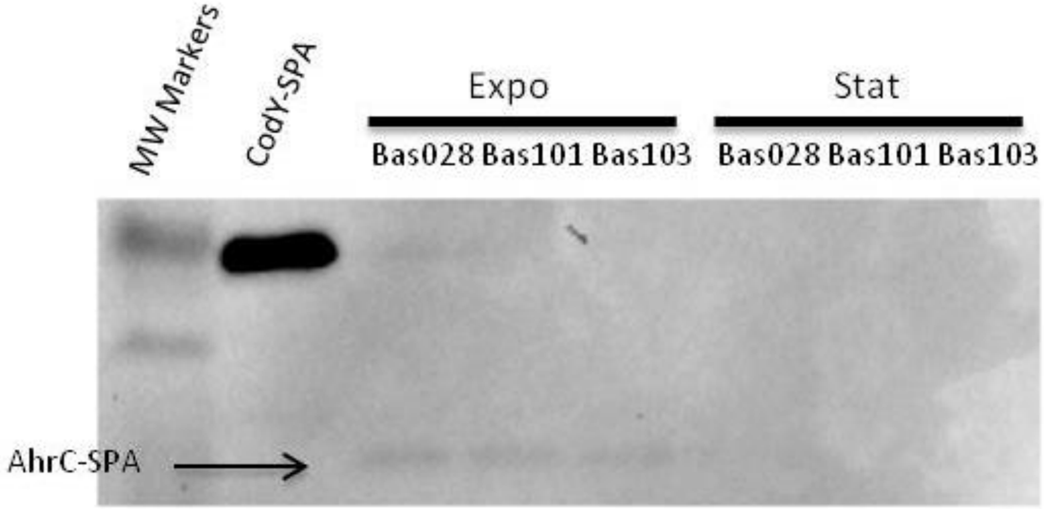

Protein samples extracted from cells carrying the *ahrC*-SPA translational fusion in *hfq*$_{Bs}$-expressing strain (Bas028) or Δ*hfq*$_{Bs}$ mutants (Bas101, Bas103) were adjusted quantitatively and separated on a 10% SDS-PAGE. As control, a sample from cells expressing CodY^{SPA} was also loaded on the gel. Faint bands of equal intensities corresponding to AhrC^{SPA} were detected in the exponential phase as indicated by the arrow in the figure. No bands corresponding to AhrC^{SPA} were visible for samples of the 3 strains taken in stationary phase of growth.
