# Supplementary material for: Tracking the Elusive Function of Bacillus subtilis Hfq
Source: PLoS One. 2015 Apr 27;10(4):e0124977. doi: 10.1371/journal.pone.0124977 (PMC4410918; doi:10.1371/journal.pone.0124977)
Supplement: S9 File — (PDF) [file pone.0124977.s009.pdf]

In silico analysis of the *hfqBs* gene conservation and synteny among the *Bacillus* genus

The conservation of *hfq*$_{Bs}$ and its genetic positioning on bacterial chromosomes was analyzed by the Archaeal and Bacterial Synteny Explorer web tool (http://archaea.u-psud.fr/absynte; ref [1]) using 49 completely sequenced genomes belonging to the *Bacillus* genus. Importantly, this analysis revealed a high degree of conservation as the *hfq*$_{Bs}$ gene was found in all the genomes. In addition, *hfq* orthologous genes were located upstream *miaA* which encodes for a tRNA isopentenylpyrophosphate transferase involved in tRNA modification,[2] a colocalisation which is conserved also among the *Proteobacteria* phylum.

References

1. Despalins A, Marsit S, Oberto J (2011) Absynte: a web tool to analyze the evolution of orthologous archaeal and bacterial gene clusters. Bioinformatics 27: 2905-2906.
2. Eisenberg SP, Yarus M, Soll L (1979) The effect of an *Escherichia coli* regulatory mutation on transfer RNA structure. J Mol Biol 135: 111-126.

Genomic contexts

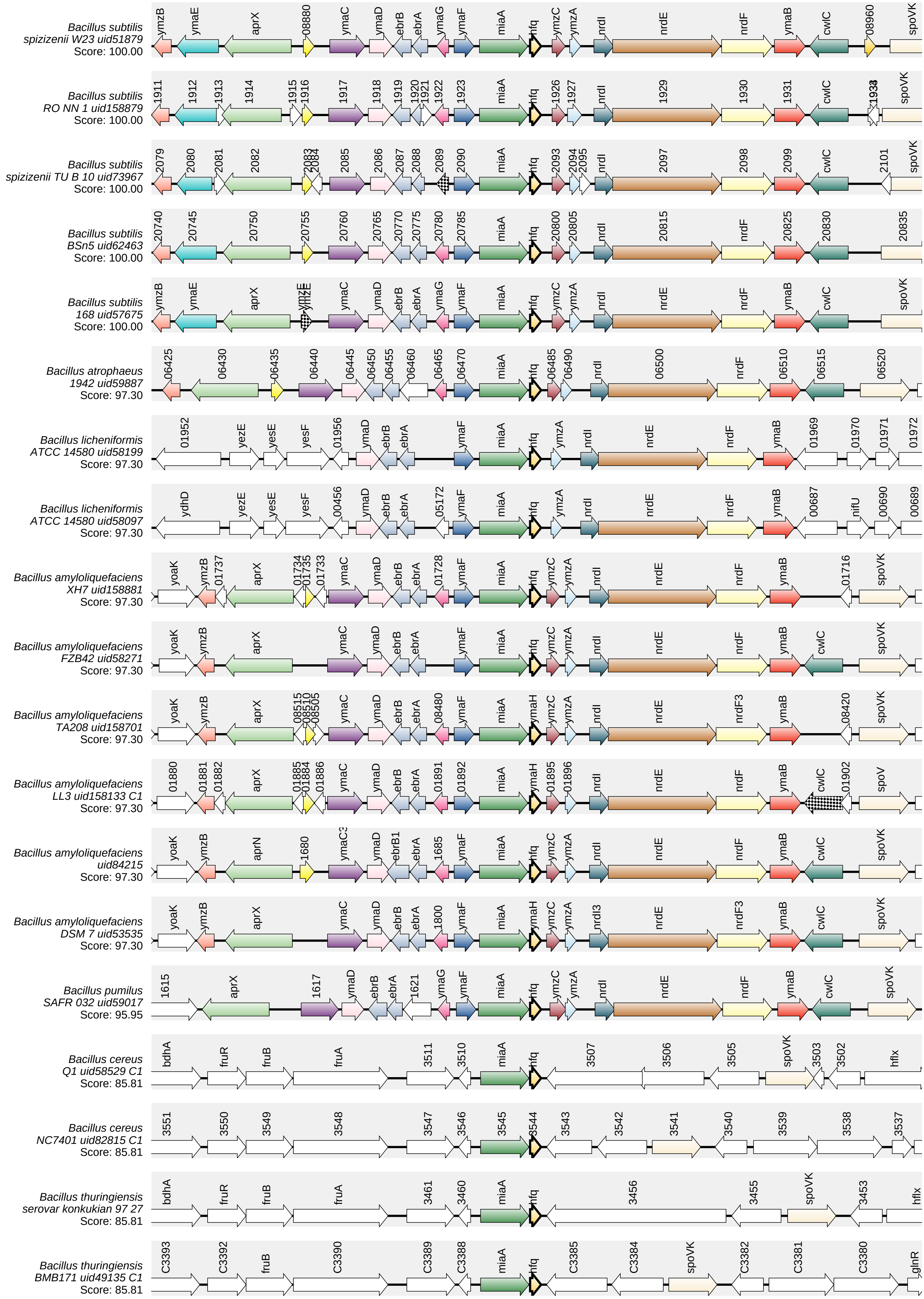

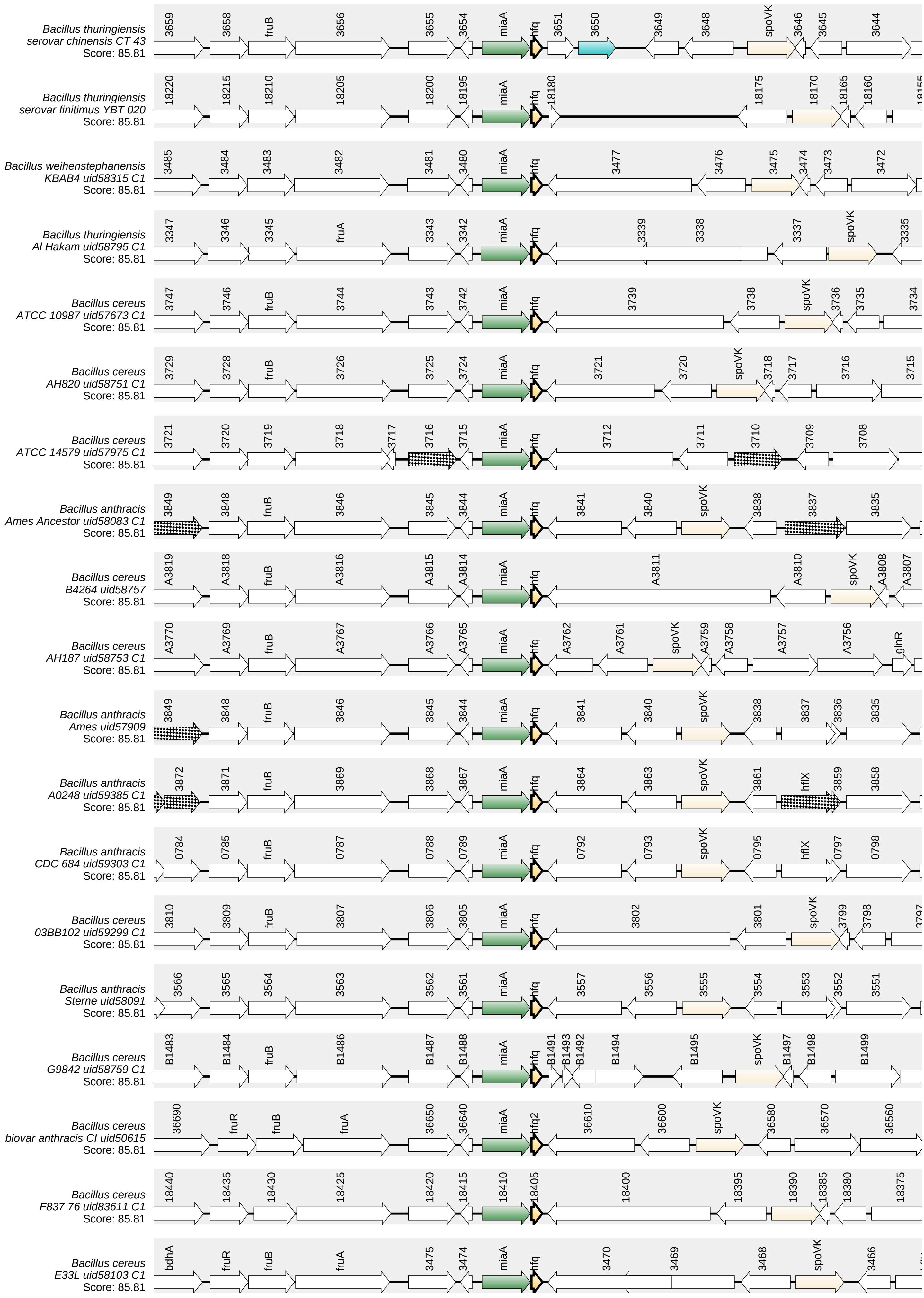
Bacillus thuringiensis serovar chinensis CT 43 Score: 85.81
3659 3658 fruB 3656 3655 3654 miaA hfq 3651 3650 3649 3648 spoVK 3646 3645 3644
Bacillus thuringiensis serovar finitimus YBT 020 Score: 85.81
18220 18215 18210 18205 18200 18195 miaA hfq 18180 18175 18170 18165 18160
Bacillus weihenstephanensis KBAB4 uid58315 C1 Score: 85.81
3485 3484 3483 3482 3481 3480 miaA hfq 3477 3476 3475 3474 3473 3472
Bacillus thuringiensis Al Hakam uid58795 C1 Score: 85.81
3347 3346 3345 fruA 3343 3342 miaA hfq 3339 3338 3337 spoVK 3335
Bacillus cereus ATCC 10987 uid57673 C1 Score: 85.81
3747 3746 fruB 3744 3743 3742 miaA hfq 3739 3738 spoVK 3736 3735 3734
Bacillus cereus AH820 uid58751 C1 Score: 85.81
3729 3728 fruB 3726 3725 3724 miaA hfq 3721 3720 spoVK 3718 3717 3716 3715
Bacillus cereus ATCC 14579 uid57975 C1 Score: 85.81
3721 3720 3719 3718 3717 3716 3715 miaA hfq 3712 3711 3710 3709 3708
Bacillus anthracis Ames Ancestor uid58083 C1 Score: 85.81
3849 3848 fruB 3846 3845 3844 miaA hfq 3841 3840 spoVK 3838 3837 3835
Bacillus cereus B4264 uid58757 Score: 85.81
A3819 A3818 fruB A3816 A3815 A3814 miaA hfq A3811 A3810 spoVK A3808 A3807
Bacillus cereus AH187 uid58753 C1 Score: 85.81
A3770 A3769 fruB A3767 A3766 A3765 miaA hfq A3762 A3761 spoVK A3759 A3758 A3757 A3756 glnR
Bacillus anthracis Ames uid57909 Score: 85.81
3849 3848 fruB 3846 3845 3844 miaA hfq 3841 3840 spoVK 3838 3837 3836 3835
Bacillus anthracis A0248 uid59385 C1 Score: 85.81
3872 3871 fruB 3869 3868 3867 miaA hfq 3864 3863 spoVK 3861 hflX 3859 3858
Bacillus anthracis CDC 684 uid59303 C1 Score: 85.81
0784 0785 fruB 0787 0788 0789 miaA hfq 0792 0793 spoVK 0795 hflX 0797 0798
Bacillus cereus 03BB102 uid59299 C1 Score: 85.81
3810 3809 fruB 3807 3806 3805 miaA hfq 3802 3801 spoVK 3799 3798
Bacillus anthracis Sterne uid58091 Score: 85.81
3566 3565 3564 3563 3562 3561 miaA hfq 3557 3556 3555 3554 3553 3552 3551
Bacillus cereus G9842 uid58759 C1 Score: 85.81
B1483 B1484 fruB B1486 B1487 B1488 miaA hfq B1491 B1493 B1492 B1494 B1495 spoVK B1497 B1498 B1499
Bacillus cereus biovar anthracis CI uid50615 Score: 85.81
36690 fruR fruB fruA 36650 36640 miaA hfq2 36610 36600 spoVK 36580 36570 36560
Bacillus cereus F837 76 uid83611 C1 Score: 85.81
18440 18435 18430 18425 18420 18415 18410 18405 18400 18395 18390 18385 18380 18375
Bacillus cereus E33L uid58103 C1 Score: 85.81
bdhA fruR fruB fruA 3475 3474 miaA hfq 3470 3469 3468 spoVK 3466

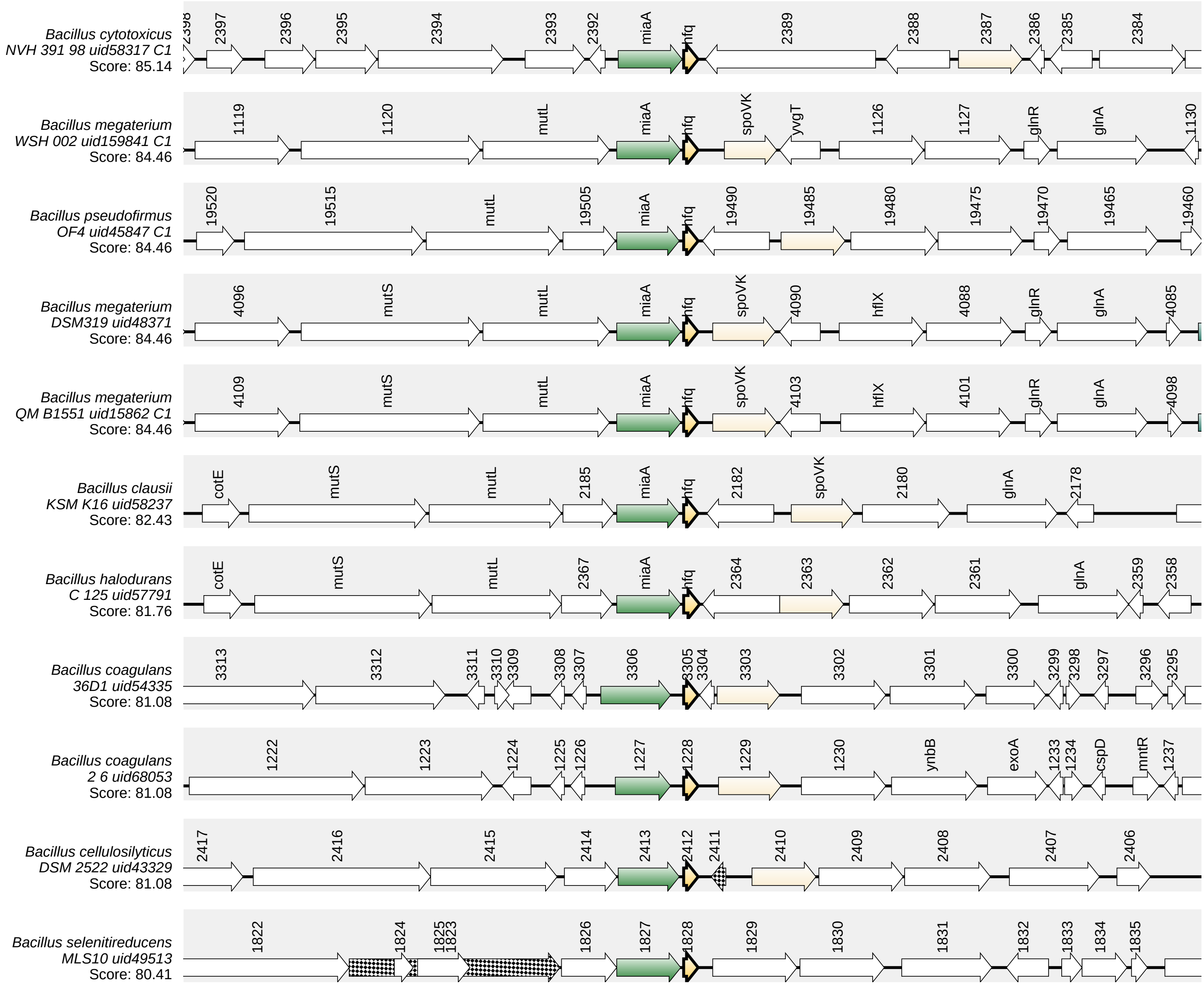

Bacillus cytotoxicus NVH 391 98 uid58317 C1 Score: 85.14
2398 2397 2396 2395 2394 2393 2392 miaA hfq 2389 2388 2387 2386 2385 2384
Bacillus megaterium WSH 002 uid159841 C1 Score: 84.46
1119 1120 mutL miaA hfq spoVK yvgT 1126 1127 glnR glnA 1130
Bacillus pseudofirmus OF4 uid45847 C1 Score: 84.46
19520 19515 mutL 19505 miaA hfq 19490 19485 19480 19475 19470 19465 19460
Bacillus megaterium DSM319 uid48371 Score: 84.46
4096 mutS mutL miaA hfq spoVK 4090 hflX 4088 glnR glnA 4085
Bacillus megaterium QM B1551 uid15862 C1 Score: 84.46
4109 mutS mutL miaA hfq spoVK 4103 hflX 4101 glnR glnA 4098
Bacillus clausii KSM K16 uid58237 Score: 82.43
cotE mutS mutL 2185 miaA hfq 2182 spoVK 2180 glnA 2178
Bacillus halodurans C 125 uid57791 Score: 81.76
cotE mutS mutL 2367 miaA hfq 2364 2363 2362 2361 glnA 2359 2358
Bacillus coagulans 36D1 uid54335 Score: 81.08
3313 3312 3311 3310 3309 3308 3307 3306 3305 3304 3303 3302 3301 3300 3299 3298 3297 3296 3295
Bacillus coagulans 2 6 uid68053 Score: 81.08
1222 1223 1224 1225 1226 1227 1228 1229 1230 ynbB exoA 1233 1234 cspD mntR 1237
Bacillus cellulosilyticus DSM 2522 uid43329 Score: 81.08
2417 2416 2415 2414 2413 2412 2411 2410 2409 2408 2407 2406
Bacillus selenitireducens MLS10 uid49513 Score: 80.41
1822 1824 1825 1823 1826 1827 1828 1829 1830 1831 1832 1833 1834 1835
