# Supplementary material for: Tracking the Elusive Function of Bacillus subtilis Hfq
Source: PLoS One. 2015 Apr 27;10(4):e0124977. doi: 10.1371/journal.pone.0124977 (PMC4410918; doi:10.1371/journal.pone.0124977)
Supplement: S2 Table — (PDF) [file pone.0124977.s011.pdf]

DNA oligonucleotides for PCRs

Primer[a]	*Sequence* (5'– 3')
1174(fw)	GGTTCAAAGTACAAATAAGCATATAAGGAAAAGAGAatgaaaccgattaatattcaggatc
1175(rv)	ATTATCCGACGCCCCCGACATGGATAAACAGCGCGTGAACctattcgagttcaagctggac
1147 (fw)	gcagatcccgaggcagcag
1148 (rv)	cctgcaggcatgcaagcttaatcggtttcatgtttcgtcctccttg
1149 (fw)	gggtaccgagctcgaattcgtccagcttgaactcgaatagatc
1150 (rv)	atttagtttagcggctggcatccg
1151 (fw)	aagcttgcatgcctgcag
1152 (rv)	gaattcgagctcggtaccc
1182 (fw)	gagtcagcgtgcaggcagattt
1183 (rv)	cctgcaggcatgcaagcttcctctcccttctaaatgtcacgcc
1184 (fw)	gggtaccgagctcgaattgtcgaaatctgcggggtggatac
1185 (rv)	agagaaaccggatcctgaatggc
1186 (fw)	caggcgataggctataaagagctg
1187 (rv)	cctgcaggcatgcaagcttggattcatgttaaaaatggccgcc
1188 (fw)	gggtaccgagctcgaattgtgtaagctgaccatgccaag
1154 (rv)	ccgccttcaaagcagtgataa
TRO80	gctatatgggctgtctcccgc
TRO81	gacgaaagggcctcgtgatacgcgtccatcatcctccttaaacataaagg
TRO82	ggaattgtgagcgctcacaattaagctgaggtgagtctgctgaaaagcgg
TRO83	ccagtttgtcacgccgtagcc
TRO84	gcgtatcacgaggccctttcgtc
TRO85	agcttaattgtgagcgctcacaattcc
TRO86	agtgggaaaaaggggaagctatca
TRO87	gacgaaagggcctcgtgatacgcctgattataacacattttccgtgaaatgc
TRO88	cagcccagtccagactattcggcgcccttgtcgttaaaaattcacaa
TRO89	cctgagaatctaacgccgaaagat
OD(fw)	ggaattggtaccaaaccgattaatattcaggatc
OD(rv)	ggaattccatggttcgagttcaagctggacg

[a] Primers are defined as "forward" (fw) or "reverse" (rv) depending on their orientation (5'-3') relative to the gene being modified. The portions of primers annealing to template DNA are in lower case. Underlined sequences correspond to the nucleotides overlapping with the antibiotic resistance gene sequence.
